# Identification of distinct maturation steps involved in human 40S ribosomal subunit biosynthesis

**DOI:** 10.1038/s41467-019-13990-w

**Published:** 2020-01-09

**Authors:** Blanca Nieto, Sonia G. Gaspar, Giulia Moriggi, Dimitri G. Pestov, Xosé R. Bustelo, Mercedes Dosil

**Affiliations:** 1 Centro de Investigación del Cáncer and Instituto de Biología Molecular y Celular del Cáncer, CSIC-University of Salamanca, Campus Unamuno, 37007 Salamanca, Spain; 2 Instituto de Biología Molecular y Celular del Cáncer, CSIC-University of Salamanca, Campus Unamuno, 37007 Salamanca, Spain; 3 Department of Cell Biology and Neuroscience, Rowan University School of Osteopathic Medicine, Stratford, NJ 08084 USA; 4 Centro de Investigación Biomédica en Red de Cáncer (CIBERONC), CSIC-University of Salamanca, Campus Unamuno, 37007 Salamanca, Spain; 5 Departamento de Bioquímica y Biología Molecular, University of Salamanca, Campus Unamuno, 37007 Salamanca, Spain

**Keywords:** RNA, Nucleolus, Ribosome

## Abstract

Technical problems intrinsic to the purification of preribosome intermediates have limited our understanding of ribosome biosynthesis in humans. Addressing this issue is important given the implication of this biological process in human disease. Here we report a preribosome purification and tagging strategy that overcomes some of the existing technical difficulties. Using these tools, we find that the pre-40S precursors go through two distinct maturation phases inside the nucleolus and follow a regulatory step that precedes late maturation in the cytoplasm. This regulatory step entails the intertwined actions of both PARN (a metazoan-specific ribonuclease) and RRP12 (a phylogenetically conserved 40S biogenesis factor that has acquired additional functional features in higher eukaryotes). Together, these results demonstrate the usefulness of this purification method for the dissection of ribosome biogenesis in human cells. They also identify distinct maturation stages and metazoan-specific regulatory mechanisms involved in the generation of the human 40S ribosomal subunit.

## Abstract (web summary)

Ribosome synthesis is a complex multi-step process. Here the authors present a method that allows the efficient isolation and characterization of the preribosomal complexes formed along the entire ribosome synthesis pathway in human cells.

## Introduction

Human ribosomes are composed of a 40S small and a 60S large subunit. The former one harbors the 18S ribosomal RNA (rRNA) and 33 proteins, whereas the latter one contains the 28S, 5.8S, and 5S rRNAs plus 47 proteins. The production of the two subunits begins in the nucleolus with the RNA polymerase I-mediated synthesis of a polycistronic 47S rRNA precursor (pre-rRNA) that contains the mature rRNAs separated by both internal (ITS) and external (ETS) RNA segments (for a scheme, see Supplementary Fig. 1a). This common precursor enters a stepwise process during which it is chemically modified, folded, endonucleolytically cleaved, and decorated by ribosomal proteins in an orderly manner. These complicated series of events require the participation of more than 200 *trans*-acting ribosome biogenesis factors (RBFs) that transiently associate with the pre-40S and pre-60S particles during their assembly and maturation in the nucleolus, nucleoplasm, and cytoplasm^1–5^.

The main features of the ribosome synthesis pathway are phylogenetically conserved. Due to this, it has long been assumed that most mechanistic and regulatory events have been conserved in humans and *Saccharomyces cerevisiae*, the organism in which ribosome synthesis has been mostly characterized up to now. Despite this, we now know that this biosynthetic pathway has to have an increased level of complexity in vertebrate species. For example, human cells display a more complex organization of the ribosomal DNA, extra pre-rRNA processing steps (Supplementary Fig. 1a), and mature ribosomes that are significantly larger than those found in yeast. It is also likely that the challenges associated with organism physiology in metazoans require new regulatory layers to fine-tune this process, as recently described for the circadian rhythm-mediated regulation of the 40S subunit synthesis in the mouse liver^6^. In line with this, recent high-throughput loss-of-function screenings have identified a significant number of human proteins with potential roles in ribosome synthesis that have no obvious yeast homologs^7,8^. There is also information suggesting that phylogenetically conserved RBFs might act differently in higher and lower eukaryotes. For example, CRM1 (also known as XPO1) and RRP12 are specifically required for the transport of preribosomes out of the nucleus in yeast, but, when depleted in human cells, cause alterations associated with defects in early pre-40S maturation steps rather than just in nuclear export^7,9^. Despite these insights, we are far from understanding how the ribosome biogenesis process is orchestrated and regulated in human cells. In addition to its fundamental interest from a biological point of view, the resolution of this problem has gained increased attention upon the discovery that defective ribosome production is associated with a set of rare human disorders called ribosomopathies^10–12^. The deregulation of the pathway in some cancer types, together with the finding of the p53-dependent nucleolar stress response, have also fueled the interest in the identification of drugs that could specifically target ribosome synthesis in cancer cells^13,14^. Unfortunately, the comprehensive dissection of the pathway in human cells has proven to be much more difficult than in yeast owing to a number of technical problems. One of them, which is related to the traditional difficulty of carrying out genetic modifications in the human genome, has been recently solved with the development of high-efficiency gene-edition techniques. However, other problems linked to the intrinsic properties of human ribosome synthesis still remain unsolved. One of them is the poor solubilization of early preribosomal particles with currently available extraction protocols, a problem probably caused by both the high viscosity of the internal nucleolar subcompartments and the presence of a thick layer of heterochromatin that surrounds the human nucleolus^15–17^. Another difficulty that contributes to the inefficient isolation of preribosomes in human cells is that, unlike the case of yeast, the ectopically expressed protein baits do not incorporate efficiently onto the preribosomal particles. Due to these problems, the vast majority of human preribosomal intermediates remain to be directly analyzed as yet. As a result, only a very limited number of late pre-40S particles have been characterized so far both at the compositional and structural level^18–20^. This is in contrast to the numerous preribosomal particles that have been successfully characterized in yeast.

To circumvent these problems, we have developed a preribosome sequential extraction (PSE) method that efficiently isolates and fractionates ribosome precursors at different stages of maturation from human cells. In addition, we have exploited the power of the CRISPR-Cas9 technology to tag endogenous RBFs to facilitate both their detection by microscopy and the pull-down of preribosomal particles from the extracts obtained with the PSE method. We have taken advantage of these new tools to gather hitherto unknown information about the maturation pathway of the small 40S ribosomal subunit.

## Results

### Isolation of preribosomal particles using the PSE method

**Fig. 1:**
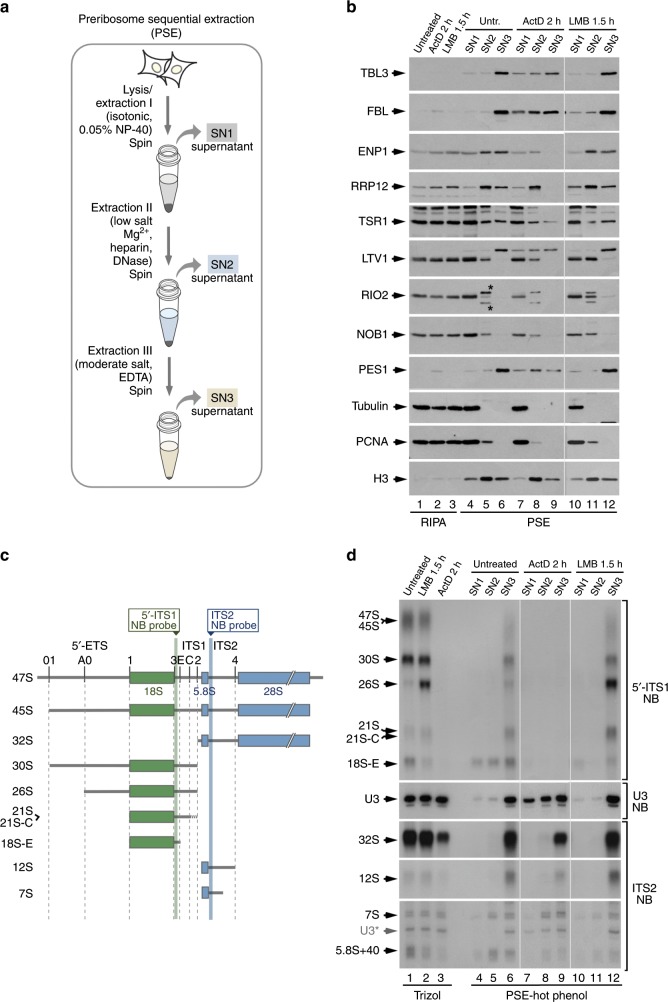
The PSE method allows sequential solubilization of preribosomal components. **a** Schematic overview of the PSE extraction method. The procedure involves differential extractions using varying concentrations of salt, Mg^2+^ and EDTA in three consecutive steps. The second step also includes chromatin removal with heparin and DNase I to facilitate the release of preribosomal particles from the nucleolus. The three extract fractions, referred to as SN1, SN2, and SN3, are collected and analyzed separately. **b** Western blot analyses showing the contents of several RBFs in extract preparations obtained either with a one-step RIPA lysis protocol (lanes 1–3) or with the PSE method (lanes 4–12) from HeLa cells untreated or treated with ActD or LMB. Control proteins include tubulin, proliferating cell nuclear antigen (PCNA), and histone H3. Non-specific detection of two proteins in the SN2 fraction with the RIO2 antibody is indicated by asterisks. Thin vertical white line separates a three-lane set that was run in a parallel gel in which control (untreated) samples and exposures were comparable. **c** Schematic diagram of the 47S primary pre-rRNA and major pre-rRNA intermediates detected with the 5′-ITS1 and the ITS2 probes used in Northern blots in this work. **d** Northern blot analyses showing the contents of different pre-rRNA species in extract preparations obtained as indicated in **b**. As control of the total content of pre-rRNA species, three samples of total RNA obtained with the Trizol method were analyzed in parallel (lanes 1–3). The detected RNA intermediates are indicated on the left and the hybridization probes on the right. The signal from the previous hybridization with the U3 probe, which is present in the bottom panels, is indicated. Thin vertical white lines separate three sets of lanes not adjacent in the original blot that were rearranged in the figure to improve clarity. NB, Northern blot. See also Supplementary Fig. 1.

We developed the PSE method to make possible the solubilization of different nucleolar compartments without compromising the integrity of preribosomes in human cells. This method involves the use of three consecutive extraction steps that utilize buffers with varying concentrations of salt and Mg^2+^. In addition, it incorporates incubations with both heparin and DNase I to remove the heterochromatin layer that surrounds the nucleolus (Fig. 1a, see details in Methods). Using Western blot analyses with antibodies to well-known RBFs of the 40S and 60S preribosome maturation ladder (for a scheme, see Supplementary Fig. 1a; for subcellular localization and other known characteristics of RBFs, see Supplementary Fig. 1b), we demonstrated that this method can efficiently extract both 40S (TBL3, FBL, ENP1, RRP12, TSR1, LTV1, RIO2, NOB1) and 60S (PES1) RBFs. By contrast, all these components are fully or partially resistant to solubilization when using RIPA buffer (Fig. 1b, compare lane 1 with lanes 4–6). Perhaps more importantly, the PSE method allows the sequential extraction of those proteins according to their specific hierarchical position in the ribosome maturation pathway. Thus, the mostly cytoplasmic (LTV1, RIO2, NOB1) and early nucleolar RBFs (TBL3, FBL, PES1) are preferentially detected in the supernatants obtained after the first (SN1) and the third (SN3) fractionation steps of the PSE method, respectively (Fig. 1b, lanes 4–6). By contrast, the nucleolar RBFs involved in more intermediate stages show distinct and more diverse extraction profiles as they can be detected in the supernatant (SN2) obtained after the second fractionation step (RRP12), the SN1 and SN2 extracts (TSR1), or the SN2 and SN3 fractions (ENP1) (Fig. 1b, lanes 4–6). This method can also extract cytoplasmic and nuclear proteins unrelated to ribosome synthesis in either the SN1 (tubulin, proliferating cell nuclear antigen) or the SN2-SN3 (histone H3) fractions (Fig. 1b). Using Northern blot analyses, we demonstrated that the PSE method can be also used to extract the pre-rRNAs associated with the late-intermediate (18S-E, 7S; Fig. 1c) and the early (U3 small nucleolar RNA (snoRNA), 30S, 26S, 21S, 32S, 12S; Fig. 1c) preribosomes in the expected SN1-SN2-SN3 and SN3 extracts, respectively (Fig. 1d, lanes 4–6). These data are consistent with the RBF fractionation pattern described above. The results indicate that the nucleoplasmic and cytoplasmic preribosomes are released at the SN1 step, the intermediate preribosomes present in more soluble or more accessible nucleolar regions are extracted in the SN2 (note that RRP12 is >80% nucleolar and is mostly concentrated in the SN2), and the early preribosomal complexes formed in inner nucleolar regions are solubilized in the SN3.

To further test the performance of the PSE method, we carried out extractions in cells that exhibit aberrant localization of preribosomal components due to either a block in pre-rRNA transcription (caused by treatment with actinomycin D (ActD)) or in nuclear export (caused by the leptomycin B (LMB)-mediated inhibition of CRM1). Nucleolar RBFs, such as TBL3, FBL, ENP1, RRP12, and PES1, accumulate in disrupted nucleolar substructures and are partially released to the nucleoplasm upon inhibition of pre-rRNA transcription (Supplementary Fig. 2a). Consistent with this, the use of our method demonstrated that the treatment with ActD leads to the redistribution of the foregoing proteins from the SN3-SN2 to the SN2-SN1 fractions (Fig. 1b, compare lanes 4–6 with lanes 7–9). By contrast, we could not detect the expected LMB-induced accumulation of nucleoplasmic ENP1-, RRP12-, and 18S-E-pre-rRNA-containing preribosomes in the SN1 fraction when using the PSE method (Fig. 1b, lanes 10–12; Fig. 1d, lanes 10–12). This is likely due to the instability and tendency to degradation of those complexes (see below). Finally, we also found that the PSE method is highly reproducible (Supplementary Fig. 2b) and applicable to different cell lines (Supplementary Fig. 2c).

**Fig. 2:**
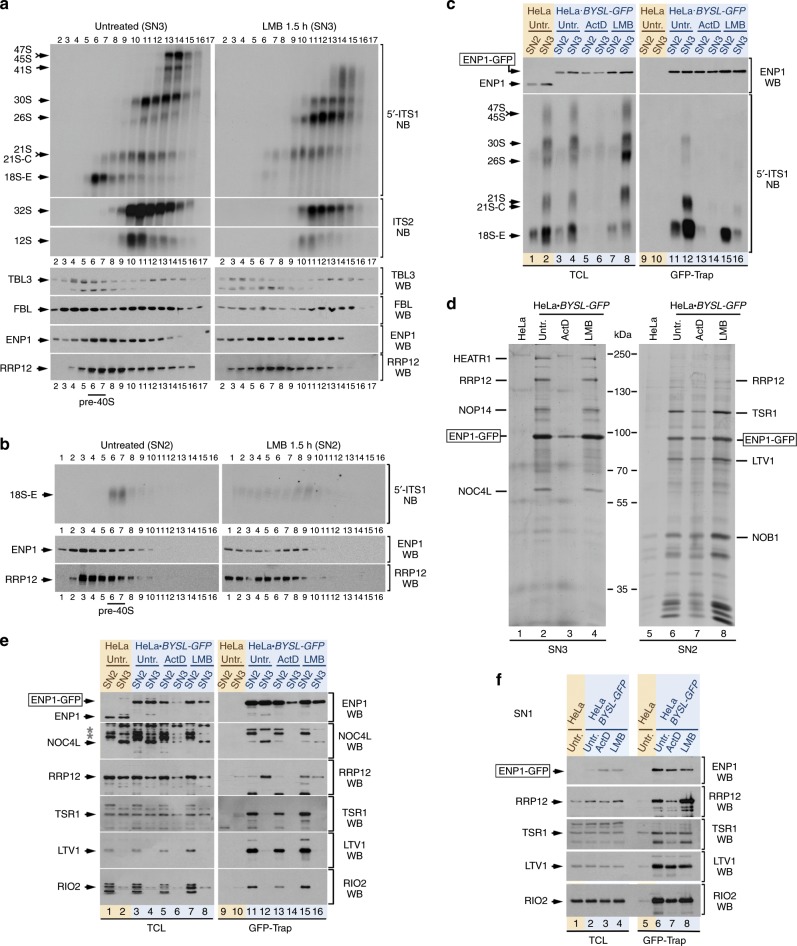
Identification of two separable pre-40S maturation stages. **a**, **b** Sedimentation profiles of the preribosomal complexes extracted in the SN3 (**a**) and SN2 (**b**) fractions of the PSE method from HeLa cells untreated or treated with LMB. The contents of pre-RNA species and RBFs in each fraction of the gradient were analyzed by Northern blot (top panels) and Western blot (bottom panels), respectively. **c** Co-purification of pre-rRNA species with ENP1-GFP extracted in the SN2 and SN3 fractions from cells untreated or treated with ActD or LMB for 2 and 1.5 h, respectively. GFP-Trap preparations from SN2 and SN3 fractions of HeLa cells and HeLa-derived (HeLa•*BYSL-GFP*) cells endogenously expressing ENP1-GFP were analyzed by Northern blot using the 5′-ITS1 probe (right bottom panel). A parallel Northern blot analyzed total RNAs prepared from the same samples used for the GFP-Trap purifications (left bottom panel). Western blot analyses revealed the ENP1-GFP content in the total fraction samples (left top panel) and GFP-Trap purification samples (right top panel). **d** Complexes formed by ENP1-GFP. GFP-Trap preparations obtained from SN3 (left panel) and SN2 (right panel) fractions of HeLa and HeLa•*BYSL-GFP* cells, untreated and treated with ActD or LMB, were resolved by SDS-PAGE. The gel was silver stained and major protein bands were sliced and identified by mass spectrometry. **e** Interactions of several RBFs with the ENP1-GFP extracted in the SN2 and SN3 fractions of the PSE method. GFP-Trap preparations were obtained as described in **c** and the amounts of bait (right top panel) and co-purifying RBFs (right second and underneath panels) were analyzed by Western blot. A parallel Western blot revealed the content of all proteins in the total fraction samples (left panels). **f** Interactions of RBFs with ENP1-GFP extracted in the SN1 fraction of the PSE method. Samples were prepared as indicated in **e**, but using the SN1 extract fractions instead of the SN2 and SN3 ones. WB: Western blot; NB: Northern blot; TCL: total cellular lysate fractions. Asterisks indicate bands from previous hybridizations of membranes with other antibodies. See also Supplementary Figs. 3, 4, and 5.

Importantly, the PSE method is also compatible with the analysis of the formation of major preribosome species present in the nucleolus using standard sucrose-gradient sedimentation analyses. Thus, we could show that the use of SN3-derived extracts in sucrose gradients allows the detection of the 47–45S pre-rRNAs and the generation of the 30S pre-rRNA within ≈90–100S preribosomes (Fig. 2a, upper left panel, fractions 13 and 14). We also observed that the 30S pre-RNA containing preribosomes undergo a maturation process, leading to progressively smaller (≈70S) complexes that are concurrent with the generation of the 21S pre-rRNA (Fig. 2a, upper left panel, fractions 14–11). Two components of early preribosomes, the RBFs TBL3 and FBL, are also detected in the very same high-molecular-weight fractions that contain the initial pre-rRNA species (Fig. 2a; left, fourth, and fifth panels from top, fractions 10–15). The maturation of 21S/21S-C-containing preribosomes (Fig. 2a, upper left panel, fractions 11–7) and the emergence of ≈40S complexes harboring the 18S-E pre-RNA (Fig. 2a, upper left panel, fractions 6–7) are also readily visualized. The RBFs ENP1 and RRP12 cosediment with the 21S/21S-C and 18S-E pre-rRNAs in the same fractions (Fig. 2a, left panels, sixth and seventh panels from top, respectively; fractions 6–9). The sedimentation patterns of all preribosomal components interrogated in these experiments do not change upon incubation of cells with LMB (Fig. 2a, right panels). This treatment, however, does induce an aberrant accumulation of the 26S pre-rRNA species (see below).

The analysis of SN2-derived extracts revealed the presence of 18S-E pre-rRNA in the ≈40S region of the sucrose gradient (Fig. 2b, top left panel, fractions 6 and 7). This indicates that these extracts also contain a pool of pre-40S particles. Both ENP1 and RRP12 are detected in the fractions that contain the 18S-E pre-rRNA, but, unlike in the case of SN3 extracts, a large proportion of these two RBFs is found in the upper fractions of the gradient (Fig. 2b, left panels, fractions 2–5). These data indicate that the preparation of pre-40S particles that are extracted in SN2 fraction include: (i) A minor pool of intact particles that contain 18S-E pre-rRNA. (ii) A major pool of particles that undergo structural disruption during the extraction procedure, giving rise to small-size subparticles. Taken together, our results indicate that this new method can efficiently extract and separate in distinct fractions the preribosomes associated with the early nucleolar (SN3 fraction), the intermediate nucleolar (SN2 fraction), and the nucleoplasmic-to-cytoplasmic (SN1 fraction) maturation steps.

### Identification of two distinct pre-40S maturation stages

The identification of two pools of 18S-E-containing particles with different solubilization properties led us to further investigate the potential application of the PSE method in the dissection of the steps involved in the production of the 40S ribosomal subunit. Since our previous experiments indicated that ENP1 is bound to all the nucleolar particles produced downstream of the pre-rRNA cleavages at sites 1 and 2 (Fig. 2a, b), we decided to use this protein as a bait to identify RBFs associated with the pre-40S pools obtained in the SN3 and SN2 fractions of the PSE method. To avoid problems previously observed with the use of ectopically expressed proteins, we decided to utilize the CRISPR-Cas9 technology to insert a green fluorescent protein (GFP)-encoding complementary DNA (cDNA) into the last exon of the *ENP1* (*BYSL*) gene in HeLa cells (Supplementary Fig. 3a). This strategy allowed us to obtain both heterozygous and homozygous cell derivatives expressing an ENP1-GFP chimera from the endogenous locus. Although relatively more enriched in the SN1 fraction than the untagged counterpart, we found that a large proportion of the ENP1-GFP is recovered in the SN2 and SN3 fractions when using the PSE method (Supplementary Fig. 4a). This extraction pattern indicates that this protein is efficiently incorporated onto the nucleolar preribosomal particles. Consistent with this, we found that ENP1-GFP is mainly localized in the nucleolus (Supplementary Fig. 4e). It is also fully functional, as inferred from the normal pre-rRNA processing profile exhibited by the tagged cells when compared to controls (Supplementary Fig. 4i, see also figure legend for further details on the functionality of the ENP1-GFP fusion protein). Using GFP-Trap to purify ENP1-GFP from a homozygous cell clone (Supplementary Fig. 4), we found that this bait can pull down the 18S-E pre-rRNA and, to a lesser extent, the 21S/21S-C pre-rRNA species from the SN3-derived extracts (Fig. 2c, lane 12). The levels of those interactions, however, are much lower in the case of ENP1-GFP purified from the SN2 fractions (Fig. 2c, compare lanes 11 and 12). This result, which is consistent with the gradient sedimentation data previously shown in Fig. 2a, b, further indicates that the pre-40S complexes extracted in the SN2 fraction include a large pool of ENP1-containing subparticles. Using mass spectrometry analyses, we found that the ENP1-GFP present in the SN3 extract is mostly associated with four nucleolar RBFs: HEATR1, RRP12, NOP14, and NOC4L (Fig. 2d, lanes 2 and 4). By contrast, the ENP1-GFP purified from SN2 extracts is found predominantly bound to late-maturation RBFs that are believed to be recruited to pre-40S particles right before exiting from the nucleolus to the nucleoplasm (TSR1, LTV1, NOB1) (Fig. 2d, lanes 6 and 8). We corroborated the different spectra of ENP1-GFP binding proteins in those two fractions using GFP-Trap purifications, followed by Western blot analyses (Fig. 2e, lanes 11 and 12). These experiments revealed that RIO2, a late-maturation factor that was not detected as a main interacting protein of ENP1-GFP in silver-stained gels (Fig. 2d), does interact with ENP1 in a minor pool of complexes specifically present in the SN2 supernatant of the PSE method (Fig. 2e). We could not assess the possible presence of PNO1, another known late-maturation RBF, in the ENP1-GFP-containing complexes using Western blot analyses due to the lack of good antibodies for this protein. As expected for interactions taking place within preribosomal complexes, we found that the associations of the RBFs with ENP1-GFP are decreased in ActD-treated cells (Fig. 2d, lanes 3 and 7; Fig. 2e, lanes 13 and 14). By contrast, they are maintained in the case of LMB-treated cells (Fig. 2d, lanes 4 and 8; Fig. 2e, lanes 15 and 16). Furthermore, we demonstrated using gradient fractionation analyses that the ENP1-GFP complexes containing HEATR1, RRP12, and NOC4L cosediment with the 18S-E pre-rRNA in ≈40S particles (Supplementary Fig. 5). By contrast, the interaction of ENP1-GFP with LTV1 detected in the SN2 fraction lacks both RRP12 and intact 18S-E pre-rRNA species (Supplementary Fig. 5). These results further indicate that ENP1-GFP behaves as the normal ENP1 (compare sedimentation profiles of ENP1 in Fig. 2a, b with those of ENP1-GFP in Supplementary Fig. 5). They also confirm that the complexes extracted in the SN3 supernatant are ≈40S preribosomes, whereas those present in the SN2 supernatant include a large proportion of <40S subparticles. Collectively, our data reveal that there are two distinctive and biochemically separable pools of ~40S precursors: (i) An earlier set of intermediates (referred to hereafter as pre40S-No1), which includes the initial complexes formed upon generation of the 18S-E pre-rRNA, contains ENP1, HEATR1, RRP12, NOP14, NOC4L, and intact 18S-E pre-rRNA species. However, it lacks cytoplasmic-maturation RBFs. (ii) A later set of intermediates (referred to as pre40S-No2), which includes complexes harboring ENP1 and cytoplasmic-maturation RBFs. These intermediate particles are less stable than the early ones and tend to generate ENP1- and RRP12-containing subparticles during the extraction procedure. To our knowledge, these two distinct pre-40S maturation stages have not been separated before in human or in yeast cells.

Finally, we observed that the ENP1-GFP recovered in the SN1 fraction establishes complexes with RRP12, TSR1, LTV1, and RIO2 (Fig. 2f). This is consistent with the presence in this fraction of pre-40S particles from both the nucleoplasm (referred to hereafter as pre40S-Nuc) and the cytoplasm (designated from now on as pre40S-Cyt) that must be undergoing the sequential release of RRP12 and late RBFs (ENP1, PNO1, TSR1, LTV1, RIO2, and NOB1) along the maturation process.

### CRM1 is not required for early maturation of 40S subunits

The foregoing results opened up the door to the identification of RBFs involved in either the formation or transport of pre-40S particles at intermediate (No1, No2, and Nuc) stages of maturation. To this end, we first decided to clarify the role of the CRM1 exportin. This protein is required for the transport of both the 40S and the 60S preribosomes out of the nucleus in yeast and humans^21,22^. However, the analysis of CRM1 was of interest because previously published data posited a human cell-specific role in the maturation of pre-40S particles. This idea stems from the observation that cells with blocked CRM1 activity exhibit, in addition to the expected defects from a block in export (e.g., the accumulation of 18S-E pre-rRNA and late-maturation RBFs in the nucleoplasm), the abnormal accumulation of the nucleolar 26S pre-rRNA species^9^. We observed that, upon inhibition of CRM1 with LMB, the pre40S-No1 particles that are pulled down by endogenous ENP1-GFP lose their preferential enrichment in the SN3 extracts and become heterogeneously distributed between the SN3 and the SN2 fractions (Fig. 2e, compare the interaction of ENP1-GFP with NOC4L and RRP12 in lanes 11–12 and 15–16). Despite this, these early precursors still keep a normal size of ≈40S (Fig. 2a, compare fractions 6 to 9 in bottom left and right panels) and protein composition (Fig. 2d, left panel, compare lanes 2 and 4) according to sucrose-gradient and mass spectrometry analyses, respectively. The inhibition of CRM1 does not alter either the composition of the more mature pre40S-No2 complexes (Fig. 2d, right panel, compare lanes 6 and 8; Fig. 2e). These results indicate that the pharmacological inhibition of CRM1 does not elicit any apparent intranucleolar pre-40S maturation defect in human cells.

**Fig. 3:**
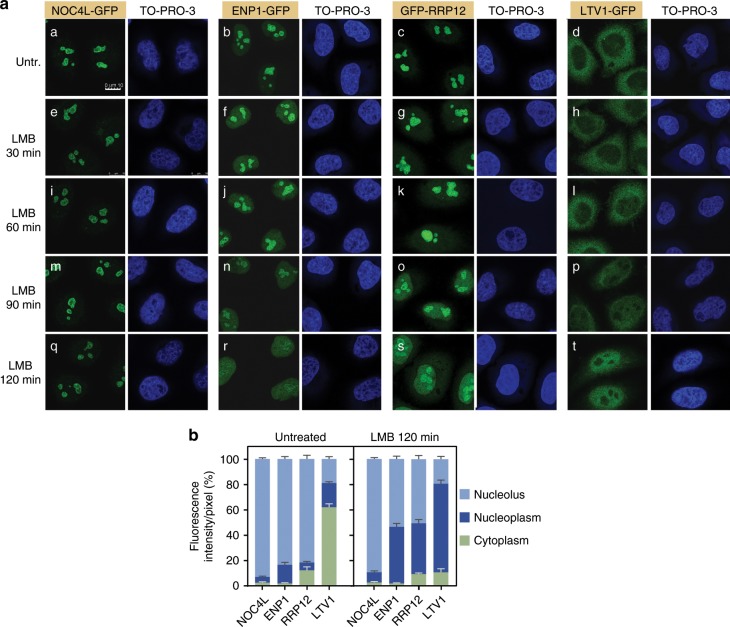
CRM1 is required for nuclear export, not for intranucleolar maturation, of pre-40S particles. **a** Nucleoplasmic accumulation of ENP1, RRP12, and LTV1, and their concomitant loss from the nucleolus (ENP1, RRP12) or cytoplasm (LTV1), caused by CRM1 inhibition. Microscopy analysis of HeLa-derived cells lines that endogenously express a fusion version of the indicated RBF after treatment with LMB for the indicated times. Green signal, GFP. Blue signal, DNA stain (TO-PRO-3). **b** Quantification of the experiment in **a** (untreated and *t* = 120 min). Relative ratios of average fluorescence intensities in the nucleolus, nucleoplasm, and cytoplasm were calculated for individual cells. Data are the mean ± s.d. from 30 cells of each condition in experimental duplicates. See also Supplementary Figs. 3 and 4. Source data are provided as a Source Data file.

To corroborate and further extend these results, we analyzed the effect of the inhibition of CRM1 on the subcellular localization of a variety of pre-40S RBFs. To facilitate these studies, we gene edited the parental HeLa cell line using the CRISPR-Cas9 methodology to incorporate GFP tags at either the C terminus (NOC4L, LTV1) or the N terminus (RRP12) of RBFs present in the No1 (NOC4L), No1/No2/Nuc (RRP12), and No2/Nuc/Cyt (LTV1) particle maturation stages (Supplementary Fig. 3). In addition, we included the previously described ENP1-GFP-expressing HeLa cell line in these analyses (Supplementary Fig. 4a, e, i). We confirmed that the GFP-tagged versions of all those proteins are fully functional in vivo (Supplementary Fig. 4, see also figure legend for details on the functionality of the GFP fusions). Using these tools, we found that the progressive accumulation of intermediate and late RBFs in the nucleoplasm of LMB-treated HeLa cells takes place in parallel to their loss from either the nucleolus (ENP1, RRP12) or the cytoplasm (LTV1) (Fig. 3a, b). By contrast, LMB does not elicit any detectable effect in the nucleolar localization of the early factor NOC4L (Fig. 3a, b). These results further confirm that the inhibition of CRM1 does not block the intranucleolar maturation of the preribosomal particles. They also unveil the progressive sequestration of intermediate nucleolar RBFs (ENP1, RRP12) in the pre40S-Nuc particles, probably as the result of defects in the export from the nucleoplasm to the cytosol. Together, our findings indicate that human CRM1, similar to its yeast homolog, is primarily involved in the export of pre40S-Nuc particles out of the nucleus. They also suggest that the observed accumulation of 26S pre-rRNA in CRM1-inhibited cells is probably a secondary effect caused by the accumulation of pre40S-Nuc particles in the nucleoplasm.

### PNO1 is required for the formation of pre40S-No1 complexes

**Fig. 4:**
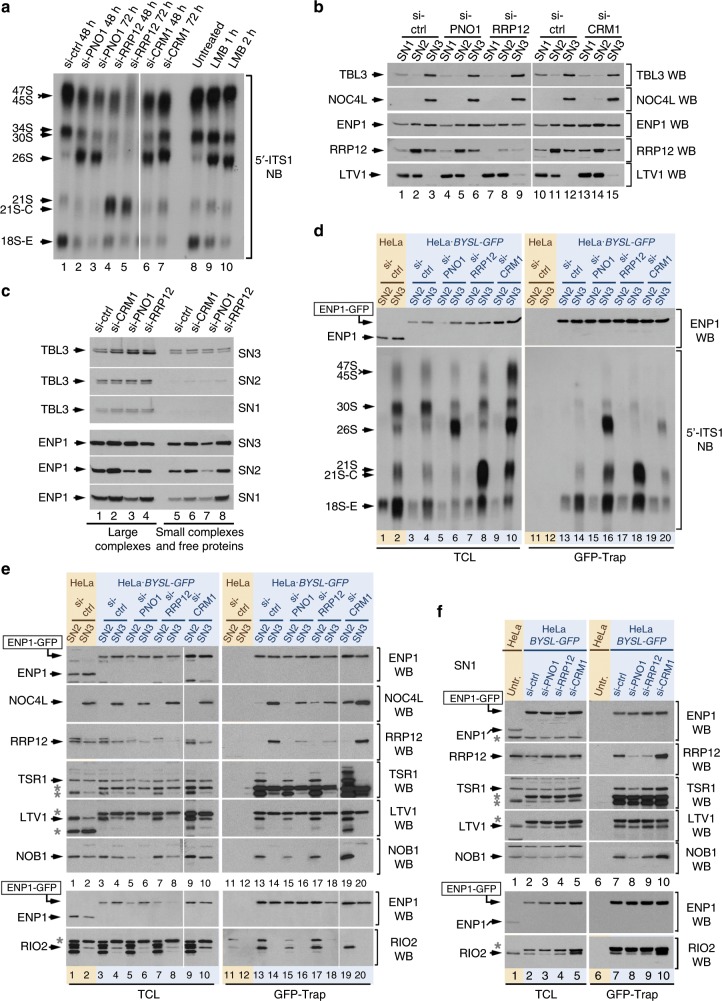
PNO1 is required for the formation of pre-40S particles and RRP12 for their maturation outside the nucleolus. **a** Relative contents of pre-rRNA processing species in HeLa cells transfected with the indicated siRNAs that were harvested at the indicated times after transfection. The 5′-ITS1 probe was used for Northern blot analysis of total RNAs prepared with the Trizol method. Thin vertical white line separates two sets of lanes not adjacent in the original blots that were rearranged in the figure to improve clarity. **b** Relative contents of several RBFs in the SN1, SN2, and SN3 fractions obtained with the PSE method from HeLa cells transfected with the indicated siRNAs and harvested 48 h after transfection. **c** Relative content of TBL3 (three top panels) and ENP1 (three bottom panels) in SN1, SN2, and SN3 fractions from cells subjected to the same experimental conditions as in **b** that were further fractionated to separate high (lanes 1–4) and low (lanes 5–8) molecular weight complexes by ultracentrifugation. **d** Co-purification of pre-rRNA species with ENP1-GFP extracted in the SN2 and SN3 fractions from HeLa and HeLa•*BYSL-GFP* cells transfected with the indicated siRNAs and harvested 48 h after transfection. Samples were processed and analyzed as indicated in Fig. 2c. **e**, **f** Interactions of several RBFs with ENP-GFP extracted in the SN2 and SN3 fractions (e), and SN1 fraction (f), from HeLa and HeLa•*BYSL-GFP* cells transfected with the indicated siRNAs and harvested 48 h after transfection. Samples were processed and analyzed as indicated in Fig. 2e, f. Thin vertical white lines in **e** separate a two-lane set that was run in a parallel gel in which control siRNA (si-ctrl) samples and exposures were comparable. The six upper panels in **e** are from one experiment. The two bottom panels are from a separate experiment that was carried out because it was not possible to obtain all antibody signals from one single blot. Asterisks in **e**, **f** indicate bands from previous hybridizations of the membranes with other antibodies. See also Supplementary Fig. 6.

**Fig. 5:**
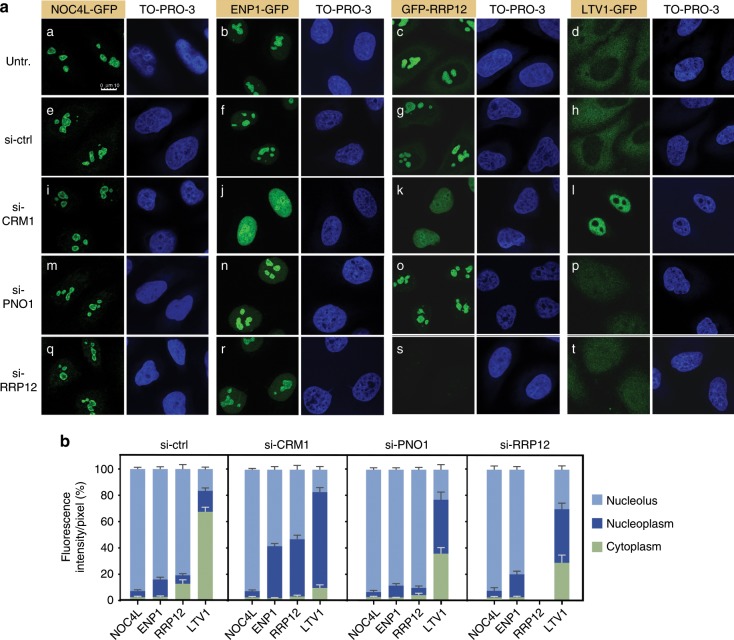
RRP12 is required for the generation of export-competent pre-40S precursors in the nucleoplasm. **a** Subcellular localization of NOC4L-GFP, ENP1-GFP, GFP-RRP12, and LTV1-GFP in CRM1-, PNO1- and RRP12-depleted cells. si-ctrl, siRNA control without target. **b** Quantification of the experiment shown in **a**. Relative ratios of average fluorescence intensities in the nucleolus, nucleoplasm and cytoplasm were calculated for individual cells. Data are the mean ± s.d. from 30 cells of each condition in experimental duplicates. Source data are provided as a Source Data file.

We next studied the involvement of PNO1 in the pre-40S maturation stages identified in this work. PNO1 is a KH-domain RBF that participates in two consecutive steps of the 40S synthesis pathway in yeast: (i) The disassembly of the 90S scaffold that precedes the emergence of pre-40S particles in the nucleolus^23^. (ii) The pre-rRNA cleavage by endonuclease NOB1 at site D (equivalent to site 3 of the 18S-E pre-rRNA in human cells) that takes place in the cytosol^24,25^. Despite this, the role of PNO1 in humans remains obscure because the small interfering RNA (siRNA)-mediated depletion of this protein yields a 26S pre-rRNA accumulation phenotype that, similarly to the case of CRM1, cannot be easily interpreted from a mechanistic point of view. To clarify the role of this protein, we monitored the production of pre-40S intermediates in HeLa cells that had been transfected for 48 h with *PNO1*-specific siRNAs. This time-point was selected because: (i) The knockdown cells display the aberrant accumulation of the 26S pre-rRNA that is typically observed in PNO1-depleted cells (Fig. 4a, lane 2). (ii) The knockdown cells still contain enough levels of preribosome intermediates to be detected in our assays (see below). For comparison purposes, we included in these experiments *CRM1*-knockdown cells that, in agreement with the results obtained with the LMB treatments shown in Fig. 2, display an accumulation of the 26S pre-rRNA species similar to that found in the case of PNO1-depleted cells (Fig. 4a, lanes 6 and 7; see also Supplementary Fig. 6a for verification of knockdown conditions). Despite this apparent similarity in phenotypes, we found that the effects of the PNO1 and CRM1 depletion are in fact different. Thus, Western blots of PSE extracts analyzed either directly (Fig. 4b, Supplementary Fig. 2d) or after further separation into large and small complexes by ultracentrifugation (Fig. 4c) revealed that the *PNO1* knockdown causes a decrease in the contents of ENP1 in SN2 and SN1 which is consistent with defects in the production of pre40S-No2 particles. The monitoring of the pre-rRNAs and RBFs bound to ENP1-GFP in the SN3 fraction also revealed that the *PNO1*-knockdown cells can generate complexes containing the 18S-E pre-rRNA (Fig. 4d, compare lanes 14 and 16). However, such complexes are either unstable or lack NOC4L and RRP12 (Fig. 4e, compare lanes 14 and 16). We could not determine the size of those complexes using sucrose gradients due to the reduced amount of pre-40S particles recovered from the SN3 extracts in siRNA-treated cells (Supplementary Fig. 6b, see low amount of 18S-E pre-rRNA in the siRNA control gradient). Consistent with a block in the generation of pre-40S particles in the absence of this protein, we did find that the interaction of ENP1-GFP with TSR1, LTV1, NOB1, and RIO2 in the SN2 extracts is abrogated in PNO1-depleted cells (Fig. 4e, compare lanes 13 and 15). This indicates that PNO1 is required for the formation of pre40S-No1 particles within the nucleolus rather than being involved in a CRM1-like exporting role. We also observed that the depletion of PNO1 leads to the aberrant accumulation of LTV1-GFP (in the nucleoplasm) and of both ENP1-GFP and GFP-RRP12 (in the nucleolus and a few small foci in the nucleoplasm) in specific subcellular compartments (Fig. 5a, panels n–p; Fig. 5b). These findings further indicate that the defect in pre-40S particle formation is associated with an unproductive docking of LTV1 and the aberrant release of both ENP1 and RRP12 from particles that are being formed in the nucleolus. Altogether, our results show that PNO1 is essential for the emergence of pre-40S particles.

### RRP12 is essential at a step that precedes pre-40S export

We followed a similar experimental avenue to analyze the role of RRP12 in 40S subunit biogenesis. This protein is required for nuclear export of 40S preribosomes in yeast^26,27^. However, its role in humans has not been characterized as of yet. Interestingly, it is observed that the knockdown of *RRP12* in HeLa cells causes an accumulation of the 21S/21S-C pre-rRNA species^7^ (Fig. 4a, lanes 4 and 5). This suggests that the RRP12 depletion leads to a defect in the maturation rather than in the nuclear export of pre-40S particles. However, this phenotype could also reflect the generation of pleiotropic or secondary defects in the knockdown cells. Further analyses of *RRP12*-knockdown cells using the PSE method revealed a slight loss of ENP1 from the SN3 fraction and a marked accumulation of free ENP1 in the SN1 fraction (Fig. 4b, c). As in the case of the *PNO1*-knockdown cells, the loss of RRP12 does not block the generation of 18S-E-containing complexes (Fig. 4d, lanes 14, 16 and 18). However, unlike what happens with PNO1-depleted cells, the association of ENP1 with NOC4L and late RBFs is not affected by the loss of RRP12 (Fig. 4e, compare lanes 13 and 14 with 17 and 18). The only change that we could observe in the RRP12-depleted cells is that the NOC4L-containing complexes become redistributed between the SN2 and SN3 fractions. This defect is similar to that observed in the case of LMB-treated and CRM1-depleted cells (see above Fig. 2e; Fig. 4e compare lanes 17 and 18 with 19 and 20). These results indicate that RRP12 is required for the downstream maturation rather than for the formation of pre40S-No1 and No2 particles.

We reasoned that if the protein were playing a CRM1-like function in nuclear export, its depletion had to promote the aberrant sequestration of pre-40S complexes in the nucleoplasm. Against this hypothesis, we could only detect minor accumulations of ENP1-GFP and LTV1-GFP in the nucleoplasm of *RRP12-*knockdown cells (Fig. 5a, compare panels f and h with panels r and t; Fig. 5b). As expected, such accumulations do occur in the case of CRM1-depleted cells (Fig. 5a, panels j and l). Further analyses confirmed that the RRP12-depleted cells contain lower levels of pre-40S complexes outside the nucleolus than CRM1-depleted cells, as inferred from the much lower amounts of NOB1 and RIO2 that are bound to the endogenous ENP1-GFP bait in the SN1 extracts (Fig. 4f, lanes 9 and 10). Yet, the detection of a fraction of pre-40S complexes in the SN1 fraction indicates that the pre40S-No2 complexes are being released in the nucleoplasm, but are either unstable or subjected to degradation in the absence RRP12 (Fig. 4f, compare lanes 7, 9, and 10). The anomalous pool of free ENP1 in the SN1 (Fig. 4c, lane 8) and the partial presence of LTV1 in the nucleoplasm (Fig. 5) are also consistent with instability of the particles that lack RRP12 and the transient accumulation of subparticle remnants in the nucleoplasm. Taken together, these results indicate that RRP12 is essential for either the formation or the stability of the pre40S-Nuc particles that have to be exported out of the nucleus. They also suggest that this function is not phylogenetically conserved given that the analogous pre40S-Nuc particles present in yeast are not degraded and tend to accumulate stably in the nucleoplasm in the absence of RRP12^27^.

### The actions of PARN and RRP12 are interrelated

**Fig. 6:**
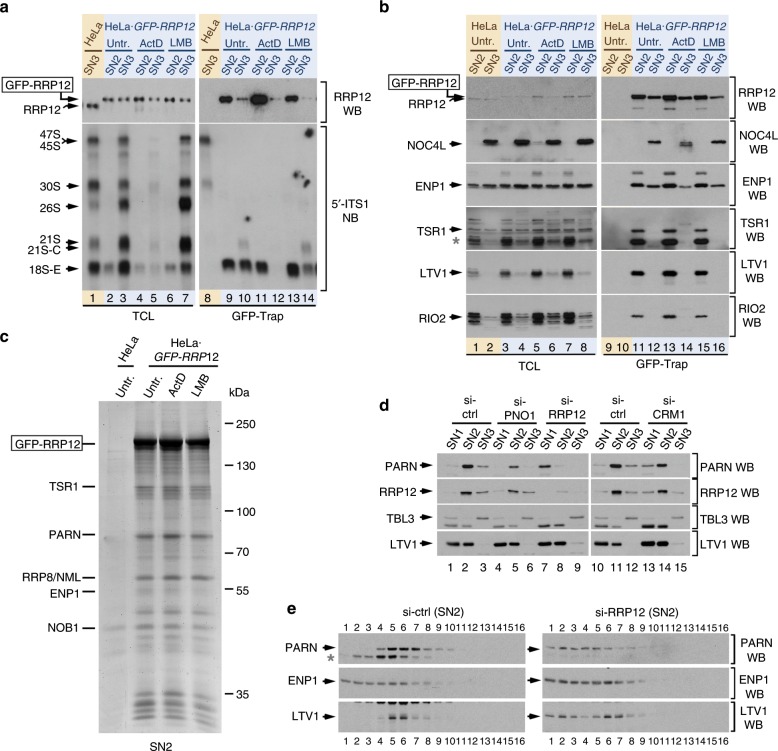
Pre-40S particles incorporate the PARN ribonuclease in the nucleolus. **a** Co-purification of pre-rRNA species with GFP-RRP12 extracted in the SN2 and SN3 steps of the PSE method from cells untreated or treated with ActD or LMB for 2 and 1.5 h, respectively. GFP-Trap preparations from SN2 and SN3 fractions of HeLa cells and HeLa-derived (HeLa•*GFP-RRP12*) cells endogenously expressing GFP-RRP12 were analyzed by Northern blot using the 5′-ITS1 probe (right bottom panel). A parallel Northern blot analyzed total RNAs prepared from the same samples used for GFP-Trap purifications (TCL, left bottom panel). Western blot analyses revealed the GFP-RRP12 content present in the total fraction samples (left top panel) and GFP-Trap purifications (right top panel). **b** Interactions of several RBFs with GFP-RRP12 extracted in the SN2 or SN3 fractions from HeLa and HeLa•*GFP-RRP12* cells untreated or treated with ActD or LMB. GFP-Trap preparations were obtained as described in **a** and the amounts of bait (right top panel) and co-purifying RBFs (right panels underneath top panel) analyzed by Western blot. A parallel Western blot analyzed the contents of all proteins in the total fraction samples (left panels). The asterisk indicates a band from previous hybridization of membranes with another antibody. **c** Proteins associated to GFP-RRP12 extracted in the SN2 step of the PSE method. GFP-Trap preparations were obtained from the SN2 fractions of HeLa and HeLa•*GFP-RRP12* cells untreated or treated with ActD or LMB. Major co-purifying bands were sliced from the gel and identified by mass spectrometry. **d** Relative content of PARN, RRP12, TBL3, and LTV1 in the SN1, SN2, and SN3 fractions obtained with the PSE method from HeLa cells transfected with the indicated siRNAs and harvested 48 h after transfection. **e** Sedimentation properties of PARN-containing complexes present in the SN2 fraction from HeLa cells after depletion of RRP12. Cells were transfected with the control or RRP12 siRNAs and collected for sucrose-gradient sedimentation analyses 48 h after transfection. The contents of PARN, ENP1, and LTV1 in each fraction of the gradients were analyzed by Western blot. Asterisk indicates a non-specific band recognized by the PARN antibody.

To characterize in more detail the pre-40S particles that contain RRP12, we next used the PSE fractionation method to analyze the RNAs and proteins that bind to endogenously expressed GFP-RRP12. In the case of the SN3 fraction, we found that GFP-RRP12 interacts with the 21S pre-rRNA (Fig. 6a, bottom panel, lane 10), the 18S-E pre-rRNA (Fig. 6a, bottom panel, lane 10), NOC4L (Fig. 6b, second panel from top, lane 12) and ENP1 (Fig. 6b, third panel from top, lane 12). By contrast, GFP-RRP12 is not bound to the late-maturation TSR1, LTV1, and RIO2 RBFs (Fig. 6b, lane 12, fourth to sixth panels from the top). In the case of the SN2 fractions, GFP-RRP12 associates with the 18S pre-rRNA, ENP1, and the late-maturation RBFs (Fig. 6a, lane 9; Fig. 6b, lane 11). These results demonstrate that the GFP-RRP12 bait can be used to pull down the pre40S-No1 and pre40S-No2 complexes present in the SN3 and SN2 fractions, respectively. This opened up the possibility to identify components of pre40S-No2 particles that remain associated with the RRP12-containing subcomplexes, but not with the ENP1-containing subcomplexes, during the extraction of cell lysates with the SN2 buffer (Fig. 2b, e).

We observed using mass spectrometry analyses that some of the GFP-RRP12 complexes obtained from the SN2 fraction contain the late-maturation RBFs TSR1 and NOB1 (Fig. 6c). This indicates that these complexes include pre40S-No2 subparticles that have TSR1 and NOB1 but not ENP1 or LTV1. We also detected the interaction of RRP12 with two additional proteins, PARN (poly(A)-specific ribonuclease) and RRP8 (also known as NML). PARN is a 3′–5′ exoribonuclease predominantly localized in the nucleolus that, in mammalian cells, has functions in messenger RNA (mRNA) turnover and maturation of non-coding RNAs (ncRNAs) such as H/ACA snoRNAs, TERC (telomerase RNA component) RNA and microRNAs^28–36^. Recent studies have also connected this protein to a 18S pre-rRNA processing step. In line with this latter function, PARN has been detected in pre-40S particles where it seems to be involved in promoting the 3′ to 5′ trimming of the 18S-E pre-rRNA after cleavage at site E^37,38^. Consistent with this idea, its depletion leads to a delay in the 3′-end trimming of this pre-rRNA^37,38^. At this moment, however, it is not known whether this defect is associated with the actual implication of PARN in the maturation of the pre-40S particle or with the impairment of some other processes that indirectly slow down the 18S-E pre-rRNA maturation. RRP8 is a nucleolar methyltransferase that, in the context of the energy-dependent nucleolar silencing complex, can suppress pre-rRNA transcription in response to glucose deprivation^39^. Given that PARN is not conserved in yeast, we decided to focus our attention on this protein as it could potentially reveal the presence of some human-specific regulatory features in the maturation of the pre-40S particles. We found using the PSE method that PARN exhibits a RRP12-like enrichment in the SN2 fraction (Fig. 6d; first and second panels from top, lanes 1 to 3). This fractionation profile is RRP12 dependent, but not PNO1 dependent or CRM1 dependent, as demonstrated by the specific shift of PARN to the SN1 fraction observed upon the depletion of RRP12 in cells (Fig. 6d). In agreement with this observation, we found that PARN is within discrete SN2-specific subcomplexes that are totally disrupted upon the RRP12 depletion (Fig. 6e). These data indicate that a vast majority of PARN is associated with RRP12-containing complexes in the nucleolus.

**Fig. 7:**
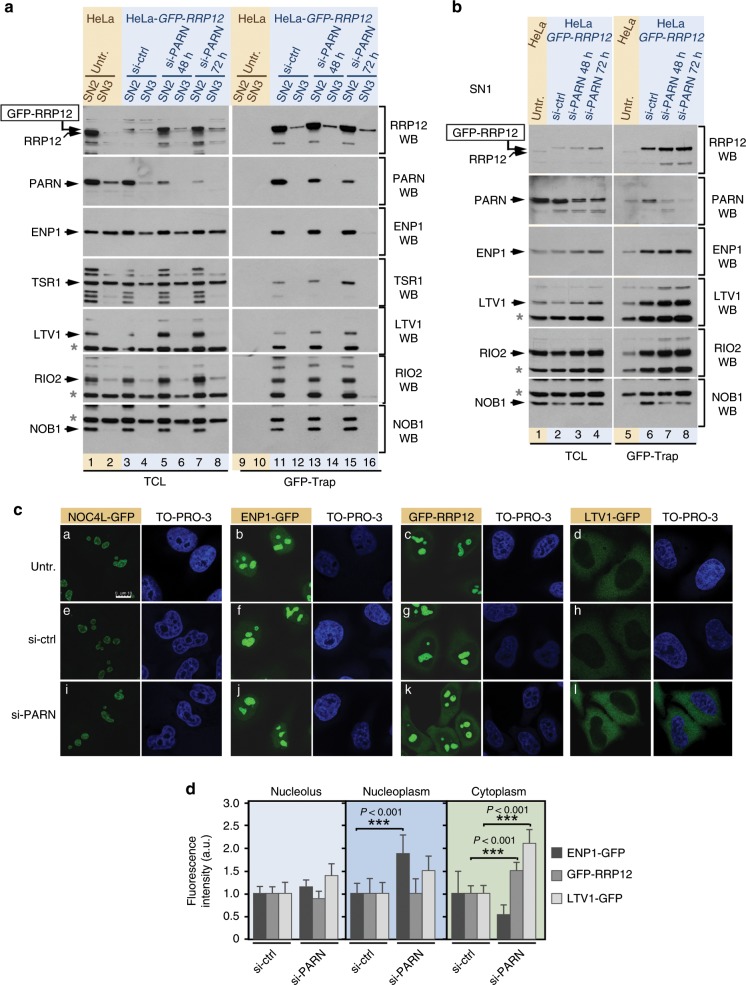
PARN is required for RRP12 release and late maturation of pre-40S particles. **a** Association of RBFs with GFP-RRP12 extracted in the SN2 and SN3 fractions of the PSE method in PARN-depleted cells. Untreated HeLa cells and HeLa•*GFP-RRP12* cells transfected with the indicated siRNAs were harvested, GFP-Trap preparations obtained and the amounts of bait (right top panel) and co-purifying RBFs (right panels underneath top panel) analyzed by Western blot. A parallel Western blot analyzed the contents of all proteins in the total fraction samples (left panels). Cells transfected with the control siRNA (si-ctrl) were analyzed 48 h after transfection. Asterisks indicate bands from previous hybridizations of membranes with other antibodies. **b** Association of RBFs with GFP-RRP12 extracted in the SN1 fraction upon PARN depletion. Cell lines, treatments and sample preparations were as indicated in **a** but using the SN1 extract fractions instead of the SN2 and SN3 fractions. **c** Subcellular localization of the indicated RBFs in PARN-depleted cells. Images were captures from HeLa-derived cell lines endogenously expressing the indicated GFP-fused RBFs untreated or transfected with the indicated siRNAs for 72 h. **d** Quantification of the changes in ENP1, RRP12, and LTV1 localization after depletion of PARN. Fluorescence signals in cell images from the si-ctrl and si-PARN samples shown in **c** were analyzed. Images were captured and processed in parallel. The fluorescence intensities (per pixel) in each cellular subcompartment were calculated relative to those obtained in the si-ctrl samples, which were given the arbitrary value of 1. Data are the mean ± s.d. from 30 cells of each condition in experimental duplicates. *P* values were determined by Mann–Whitney test. Source data are provided as a Source Data file. See also Supplementary Fig. 7.

Based on the above, we next investigated whether the previously reported 18S-E pre-rRNA processing activity of PARN could be required for the maturation of either the pre40S-No2 or the downstream pre40S-Nuc complexes (both of which contain RRP12). To explore this possibility, we tracked down the formation of those complexes in *PARN*-knockdown cells. The depletion of this protein induces the expected increase in the abundance of 18S-E pre-rRNA (Supplementary Fig. 7a), and the concomitant accumulation of RRP12, ENP1, TSR1, LTV1, and RIO2 in the SN1 fraction (Supplementary Fig. 7b). By contrast, it does not affect the distribution pattern of TBL3 in those fractions (Supplementary Fig. 7b, second panel from top). TBL3 is a factor associated with early preribosomes generated upstream of pre-40S particles. Further analyses indicated that the RRP12-containing complexes from the SN1 fraction of PARN-depleted cells contain LTV1 and RIO2 (Fig. 7b). However, they lack NOB1 and display a partial release of ENP1 (Fig. 7b). The interaction of GFP-RRP12 with all those RBFs in the SN2 extract does not change in the absence of PARN (Fig. 7a, lanes 11, 13, and 15). These results indicate that the *PARN* knockdown does not impair the formation of pre40S-No2 particles, but it does affect their downstream maturation outside the nucleolus. Consistent with this idea, we found that the depletion of PARN leads to the accumulation the GFP-RRP12 and LTV1-GFP in the cytoplasm of HeLa cells (Fig. 7c, d). It also promotes, in agreement with our protein–protein interaction data, a slight accumulation of ENP1 in the nucleoplasm (Fig. 7c, d). No overt alterations in the normal nucleolar localization of NOC4L are observed in *PARN*-knockdown cells (Fig. 7c), further indicating that the loss of PARN does not affect the assembly of earlier 40S subunit precursors. These findings indicate that RRP12 is not properly released from pre-40S precursors in PARN-depleted cells. In addition, they show that the block in the release of RRP12 compromises the final pre-40S particle reconfiguration, as demonstrated by the block in the release of LTV1 but not of NOB1 from those particles. Altogether, these data indicate that PARN is a bona fide 40S subunit maturation factor that is required for the completion of a RRP12-mediated maturation step that precedes the late-maturation phase of pre-40S particles in the cytoplasm.

## Discussion

Here, we have reported a protocol that facilitates the isolation of preribosomal particles from different cellular compartments in human cells. This strategy, together with the CRISPR-Cas9-mediated GFP tagging of endogenous RBFs, has allowed us to identify distinct transitional pre-40S stages, clarify hitherto obscure roles of some RBFs, and uncover human-specific maturation events linked to the biosynthesis of the 40S ribosomal subunit.

Previous proteomic and biochemical studies of preribosomes in yeast provided a wealth of information about ribosome maturation in that organism^1,2^. By contrast, the isolation of preribosomes from human cells has proven a more difficult challenge due to problems associated with the incomplete extraction of preribosomal complexes from the nuclei and the loss of integrity of the large pre-rRNA intermediate species. To improve the release of preribosomal particles, early studies incorporated the sonication-based isolation of nucleoli from mammalian cells^40^. This is a rather laborious approach that requires the use of large amounts of cells. In other cases, the isolation of these particles was improved using harsh extraction methods. For example, in one classic protocol^41^, the nuclei were treated with DNase in high salt buffer (0.5 M NaCl, 50 mM MgCl_2_) and then the ribonucleoprotein complexes extracted using a chelator under low ionic strength conditions (10 mM NaCl, 10 mM EDTA). Our attempts to purify preribosomes using this technique were hampered by the instability of the preribosomes and their tendency to aggregate during the incubation with affinity matrices. We surmise that this problem is caused by the stripping of many RBFs from these complexes during the extraction method. The PSE approach introduced here circumvents many of those technical problems. This protocol has been developed taking into consideration our own empirical observations, indicating that preribosomal particles at different stages of maturation can be efficiently released by altering ionic strength and divalent cation concentrations after the heparin- and the DNase I-mediated removal of the nuclear material that surrounds the nucleoli in mammalian cells. As we have shown here, this technique preserves the integrity of large rRNA precursors and offers a simple, high-throughput approach to isolate human preribosomal complexes from low amounts of starting material. Importantly, this method is fully compatible with standard analytical procedures for the characterization of preribosomal particles, such as protein immunoprecipitation, mass spectrometry, and sucrose-gradient fractionation analyses. The method has also enough sensitivity to monitor changes in the solubilization pattern of the interrogated proteins, complexes, and pre-rRNAs under diverse experimental conditions (e.g., siRNA transfection and drug treatments).

A key achievement of our study is the isolation of two distinct pools of 18S-E pre-rRNA-containing particles that represent two maturation stages of the 40 S subunit inside the nucleolus. These intermediate precursors have not been detected in previously published studies using mammalian or yeast cells^42,43^. This was probably due to the overlapping composition of these particles with other preribosomes generated downstream in the route, and by the tendency of some of them to disassemble during extraction at least in the case of human cells. The isolation of these particles could only be accomplished through the use of the PSE method. Our data indicate that the initial ≈40S preribosomes, designated here as pre40S-No1 particles, enter a separable maturation phase in the nucleolus during which they acquire the RBFs needed for ensuing downstream maturation events both in the nucleoplasm and cytoplasm (Supplementary Fig. 8). The complexes undergoing this maturation phase, designated here as pre40S-No2 particles, are rather distinct from the pre40S-No1 precursors according to proteomic composition, solubility properties and structural stability. A majority of pre40S-No2 intermediates tend to undergo disruption and generate small-size subparticle complexes during their extraction in the SN2 step. Given that the integrity of ≈40S preribosomes is preserved in the subsequent extraction step (SN3), this instability is probably due to some structural feature that is specific of the particles solubilized in the SN2 step rather than to a technical problem associated with the use of the PSE method. Notably, a distinctive component of pre40S-No2 particles is the PARN exoribonuclease. This might well explain the structural instability of those complexes, because the 3′ end of the 18S-E pre-RNA is likely to be exposed in order to undergo the PARN-mediated maturation step. An additional idea that emerges from our results is that the No1 and No2 intermediates might be physically segregated in different subcompartments of the nucleolus given their distinctive solubilization in the SN2 and SN3 extracts obtained with the PSE method. Such separation cannot be explained by a directional movement of the No2 particles from inner to outer regions of the nucleolar granular component because, for example, the SN2-specific RRP12 and the SN3-specific PES1 proteins display a similar distribution throughout the granular component according to microscopy analyses (this work and ref. ^44^). The differential extractability of the No1 and No2 complexes in distinct fractions of the PSE method suggests that the granular component might be composed of separate liquid-like phases endowed with different biophysical properties. In favor of this possibility, the existence of such phases has been recently postulated to cause the separation of the dense fibrillar and granular components in the nucleoli of *X. laevis* cells^16^.

The use of the PSE method also enabled us to clarify the roles of specific proteins in human 40S subunit synthesis and, perhaps more importantly, identify hitherto unknown regulatory steps and factors involved in them. For example, we could demonstrate that PNO1 and CRM1 play similar roles to those previously found in the case of their respective yeast homologs. These data rule out the potential implication of CRM1 in upstream maturation steps that had been inferred from the detection of pre-rRNA processing defects in *CRM1*-knockdown cells. We have also unveiled a human-specific regulatory step that entails the coordinated action of both RRP12 and PARN. We have shown that the former protein is essential for the generation of the pre-40S particles that have to be exported by CRM1 to the cytoplasm. Consistent with this, we have observed that the depletion of RRP12 triggers the formation of highly unstable particles that, unlike the case of the inhibition of CRM1, appear to be rapidly targeted for degradation within the nucleoplasm. The formation of these unstable pre-40S particles does not take place when RRP12 is depleted in yeast cells, suggesting that the human protein might be involved in a quality-control mechanism that ensures the transport of correctly formed pre-40S particles to the cytoplasm in human cells. Interestingly, a recent high-resolution structural study has shown that RRP12 is bound to the head region in a subset of pre-40S particles^18^. The head of those particles displays an immature configuration, indicating that the essential role we found for RRP12 in our study could be related to the regulation of the proper maturation of the head region before pre40S-Nuc particles are transported to the cytoplasm. Our work has also revealed that RRP12 is required for the docking of PARN onto the pre40S-No2 particles. The binding of PARN, in turn, is a condition sine-qua-non for the subsequent release of RRP12 from pre-40S particles that takes place before the final maturation steps in the cytoplasm. The function of PARN in this process could be related to its known ability to remove 3′ adenosine tails and the subsequent trimming of the 3′ ends of a variety of ncRNA processing intermediates to finalize their maturation^28,31–33,35^. We speculate that this protein might be involved in shortening of the ITS1 segment of the 18S-E pre-rRNA that can facilitate the last step of the 40S subunit maturation in the nucleoplasm. Supporting this idea, a recent report has described that the 18S-E pre-rRNA becomes polyadenylated under certain physiological conditions^6^. Moreover, this process has been associated with a block in the maturation of the 40S preribosome in the nucleoplasm^6^. The recent structural study of late human pre-40S particles has not identified any PARN-containing precursors, further highlighting the utility of our extraction method for the characterization of pre-40S intermediate states. Importantly, the RRP12-PARN regulatory step is probably restricted to both plant and animal metazoans, since there are no PARN homologs in yeast and invertebrate species. Mutations in PARN have been associated with several rare human syndromes, including the ribosomopathy dyskeratosis congenita^45–48^. It will be therefore interesting to evaluate if the PARN-mediated 40S synthesis regulatory step described in this study is altered in those pathologies.

It is likely that the further exploration of the factors that interact with the pre-40S intermediates identified here will help to pinpoint additional regulatory steps and proteins involved in the synthesis of 40S ribosomal subunits. The experimental strategy implemented in this work could also help to elucidate mechanistic aspects of the maturation pathway of the 60S ribosomal subunit. This is demonstrated, for example, by the efficient solubilization and good behavior in gradient sedimentation analyses of both the 32S and 12S pre-rRNAs obtained in the SN3 extracts (Fig. 2a). It will be also possible to use this method to identify and dissect the alterations in the assembly, compartmentalization, and/or export of preribosomal particles in ribosomopathies and other diseases.

## Methods

### Generation of GFP knock-in edition plasmids

Genomic editing of each individual locus required the generation of two plasmids (listed in Supplementary Table 1). One of the plasmids drives the expression of both the Cas9 nuclease and a scaffold/guide RNA (sgRNA). The other plasmid carries the DNA donor for homology-directed repair (HDR). The DNA donor contained the GFP sequence in-between two DNA segments homologous to the genome region to be edited. For the generation of the Cas9/sg plasmid, guide sequences were chosen using open-access online tools (crispr.mit.edu and benchling.com) that take into consideration the protospacer adjacent motif (PAM) sequences in the genomic region as well as the on-target and off-target scores. The genomic contexts of sg guide sequences used for the editing of *ENP1*, *NOC4L*, *RRP12*, and *LTV1* loci are shown in Supplementary Fig. 3. The sg sequences were cloned into the plasmid pX330 using synthetic oligonucleotides that were annealed and directly ligated to *Bbs*I-digested vector^49^. Sequences of the sg oligonucleotides used for each locus are listed in Supplementary Table 2. For the generation of the second plasmid, the HDR donor sequences were introduced into a cloning vector. The HDR plasmids (pBYSL-GFP-BYSL, pNOC4L-HDR) for the *BYSTIN* (NCBI gene ID: 705) and *NOC4L* (gene ID: 79050) loci, which contained the GFP cDNA sequence fused in-frame with the corresponding last codon, were generated by gene synthesis (GeneArt^©^, Invitrogen, Life Technologies). In the case of the NOC4L plasmid, a silent mutation in the PAM sequence [Ala 510 (GCC–>GCA)] was introduced to avoid cleavage of the repair template by Cas9. The HDR plasmids for the *RRP12* (gene ID: 23223) and *LTV1* (gene ID: 84946) loci were generated by sequential subcloning of genomic and GFP fragments. Genomic fragments were PCR amplified from HeLa cells, with the exception of the *LTV1* left-arm fragment that was obtained by gene synthesis (GeneArt^©^, Invitrogen, Life Technologies). For the generation of the *RRP12* HDR plasmid (pBN81), the right-arm fragment was cloned into pEGFP-C1 (Clontech) at *Xho*I-*Hin*dIII sites, generating a fusion of the last codon of *GFP* with the first codon of *RRP12*. Next, an *Nhe*I-*Mfe*I fragment containing the GFP and the right arm was excised and cloned into pBluescript (Stratagene) at the *Spe*I-*Eco*RI sites. Finally, the *Nhe*I site was restored by site-directed mutagenesis and the left-arm fragment was introduced at the *Nhe*I-*Age*I sites of pBluescript. In the case of the *LTV1* HDR plasmid (pGH1), the left-arm fragment was cloned into pEGFP-C1 at the *Xho*I-*Hin*dIII sites, generating a fusion of the last codon of *LTV1* with the first codon of *GFP*. Next, an *Nhe*I-*Mfe*I fragment containing the left arm and the GFP was excised and cloned into pBluescript (Stratagene) at the *Spe*I-*Eco*RI sites. Finally, the right-arm fragment was introduced at the *Xho*I-*Kpn*I sites of pBluescript. The construction of the *PES1-GFP* cell line (only used in Supplementary Fig. 2a) will be described in a future publication. The sequences of all oligonucleotides used for the generation of HDR donor plasmids are shown in Supplementary Table 2.

### Cell treatments and selection of genetically modified lines

The HeLa cell line was obtained from ATCC and the HCT116 line was kindly provided by professor María Sacristán of Centro de Investigación del Cáncer of Salamanca. Both cell lines were cultured in Dulbecco’s modified Eagle’s medium supplemented with 10% fetal bovine serum, 100 U/ml penicillin/streptomycin, 2 mM l-glutamine (Gibco), and maintained under standard tissue culture conditions. RNA polymerase I transcription was inhibited by 100 ng/ml ActD (Calbiochem) and CRM1 was inhibited by treatment with 40 nM LMB (Enzo). To knock down the expression of specific genes, siRNA duplexes (listed in Supplementary Table 3) were purchased from either Ambion (Silencer Select siRNA) or Invitrogen (stealth siRNA against PARN) and used to reverse transfect cells using Lipofectamine RNAiMAX (Life Technologies) as previously described^7^. Cells were harvested 24, 48, or 72 h after transfection, as indicated in the figures. Negative controls were either untreated cells or cells transfected with a control scrambled siRNA. For the GFP knock-in, cells were transfected with 1–3 μg of a mixture of Cas9/sg and HDR plasmids (1:2 molar ratio) using Lipofectamine 2000 (Life Technologies), re-transfected with the HDR plasmid 24 h after the first transfection and sorted on the basis of GFP fluorescence intensity 4–5 days after the second transfection. Individual GFP-positive cell clones were isolated, expanded, and analyzed by both Western blot and PCR to select those carrying the knock-in modification in all the alleles of the targeted locus.

### PSE method

The starting material was two 10 cm dishes of HeLa or HCT116 cells grown at **≈**80% confluency. Cells were harvested on ice-cold phosphate-buffered saline, frozen in liquid nitrogen in Eppendorf tubes, and kept at −80 °C until use. The cell pellets were resupended thoroughly in 0.5 ml of SN1 buffer (20 mM HEPES-NaOH [pH 7.5], 130 mM KCl, 10 mM MgCl_2_, 0.05% Igepal CA-630), supplemented with Cømplete protease inhibitor cocktail (Roche), followed by centrifugation in a microfuge (1300 × *g*, 3 min, 4 °C). The resulting supernatant was collected and stored as the SN1 fraction. The pellet was washed with 0.5 ml SN1 buffer, and then resuspended in 0.3 ml of SN2 buffer (10 mM HEPES-NaOH [pH 7.5], 10 mM NaCl, 5 mM MgCl_2_, 0.1% Igepal CA-630, 0.5 mg/ml heparin, 600 U/ml RNasin (Promega)] supplemented with 100 U RNase-free DNase I (Qiagen), and incubated for 10 min at room temperature with gentle mixing. The lysate was centrifuged (12,300 × *g*, 10 min, 4 °C) and the supernatant collected as the SN2 fraction. The remaining pellet was resuspended in 0.4 ml of SN3 buffer (20 mM HEPES-NaOH [pH 7.5], 200 mM NaCl, 4 mM EDTA, 0.1% Igepal CA-630, 0.04% sodium deoxycholate, 4 mM imidazole, 0.1 mg/ml heparin, 1 mM dithiothreitol (DTT), Cømplete, 600 U/ml RNasin) and incubated for 20 min at room temperature with moderate agitation. The extract was centrifuged (12,300 × *g*, 10 min, 4 °C) and the supernatant was collected as the SN3 fraction. For the analysis of protein content in each fraction, samples from equivalent volumes of the SN1, SN2, and SN3 fractions (ratio 10:6:8) were directly analyzed by Western blot. In some analyses, such as the one shown in Fig. 1b, 50 μg samples of whole-cell lysates prepared with RIPA buffer were analyzed in parallel. Quantifications of signals in Western blots were measured using ImageJ^TM^ software (NIH) and data from experimental triplicates were combined to calculate mean and standard deviation values. For the analysis of pre-rRNA species in the PSE samples, total RNAs were prepared from equivalent volumes of the PSE fractions (120 μl of SN1, 72 μl of SN2, and 96 μl of SN3) using the hot-phenol method^50^. The isolated RNAs were resuspended in formaldehyde-loading buffer and analyzed by Northern blot in parallel with 4 μg of total RNAs prepared from whole cells using the TRI reagent (Life Technologies) according to the manufacturer’s protocol.

For the experiment in which PSE fractions were further fractionated into small and large molecular weight complexes (Fig. 4c), we used HCT116 cells. The separation of small and large complexes was optimized in these cells using experiments in which the nucleolar stress response was induced by ActD to provoke an accumulation of free RPL5/RPL11 complexes (data not related to this study). Six 10 cm plates of HCT116 cells were used for each condition and samples of the PSE fractions [200 μl of SN1, 200 μl of SN2, and 100 μl of SN3 (taken to 200 μl with SN3 buffer)] were ultracentrifuged at 155,000 × *g* in a TLA-100 rotor for 120 min at 4 °C. Pellets were resuspended in the same original buffer and volume. Aliquots of those samples (containing the high-molecular-weight complexes) and of the corresponding ultracentrifugation supernatant (containing low-molecular-weight complexes) were analyzed by Western blot.

### Total RNA preparation and Northern blot analysis

Whole-cell total RNAs were extracted by the Trizol method using the TRI reagent. Quantifications were performed using a Nanodrop (VWR) espectrophotometer. RNAs from the three supernatants obtained with the PSE method, sucrose-gradient fractions, or GFP-Trap-purified complexes were prepared by the hot-phenol method. RNA preparation, oligonucleotide labeling, RNA separation, Northern blotting, and hybridizations were performed following standard procedures^51^. All Northern blot analyses were performed on RNAs separated on 1.2% agarose/formaldehyde gels, except in the case of 7S and 5.8S pre-rRNAs, which were performed on RNAs resolved on 8% acrylamide-urea gels. The sequences of the oligonucleotides used as probes are shown in Supplementary Table 2.

### Total protein preparation and Western blot analyses

Total cellular lysates were prepared following a protocol described by Castle et al.^52^. Briefly, cells were lysed in RIPA buffer (25 mM Tris-HCl [pH 7.6], 150 mM NaCl, 1% NP-40, 1% Triton X-100, 1% sodium deoxycholate, 0.1% sodium dodecyl sulfate [SDS]) supplemented with Cømplete protease inhibitor cocktail, kept on ice for 20 min, and then lysates were cleared by centrifugation (12,300 × *g*, 10 min, 4 °C). Protein concentrations in the cleared supernatants were determined with Precision Red reagent (Cytoskeleton) according to the manufacturer’s instructions. The sources and dilutions of primary antibodies used in Western blot analyses are shown in Supplementary Table 4. Primary antibodies were detected by horseradish peroxidase-conjugated secondary antibodies to rabbit and mouse immunoglobulins (GE Healthcare) and the Pierce ECL Western Blotting Substrate (Thermo).

### Sucrose-gradient analysis

Five 10-cm plates of HeLa cells (**≈**80% confluent) were used to prepare extracts with the PSE method as described above, although doubling the total volumes of the buffers used in the SN1, SN2, and SN3 steps. SN2 and SN3 fractions were prepared and two aliquots were stored, one (one-twentieth of the total fraction volume) to directly analyze protein content and the other one (one-tenth of the total fraction volume) to prepare total RNA by the hot-phenol method. The rest of the fraction sample was loaded onto linear 7–50% sucrose gradients containing 20 mM HEPES-NaOH (pH 7.5), 200 mM NaCl, 4 mM EDTA, 0.1% Igepal CA-630, 0.1 mg/ml heparin, and 1 mM DTT. Ultracentrifugation was performed on a SW40 (Beckman) rotor at 192,000 × *g* for 165 min at 4 °C. Fractions (0.5 ml) were collected on a gradient collector system (ISCO) and subsequently processed for Western and Northern blot analyses. For protein analyses, 360 μl samples from each fraction were precipitated with 12.5% trichloroacetic acid, washed with acetone, and resuspended in SDS-polyacrylamide gel electrophoresis (SDS-PAGE) loading buffer before being loaded onto SDS-PAGE polyacrylamide gels. For pre-rRNA analyses, 120 μl samples from each fraction were taken to prepare total RNAs by the hot-phenol procedure^51^. RNAs were finally resuspended in formaldehyde-loading buffer and loaded onto 1.2% agarose-formaldehyde gels. For the experiment shown in Supplementary Fig. 5, the protocol was scaled up and five sucrose gradients for each one of the supernatants (SN3 and SN2) were run in parallel. The corresponding fractions of the five gradients were pooled, aliquots were taken for Northern and Western blot analyses as described above, and the rest was combined to generate the four pools indicated in the figure that were used for purification of ENP1-GFP with GFP-Trap.

### Protein–RNA and protein–protein co-purification experiments

GFP-tagged proteins were purified from SN1, SN2, and SN3 fractions obtained from five 10-cm dishes of HeLa cells using GFP-Trap. Aliquots were taken for protein content analysis (one-twentieth of the whole volume) and total RNA preparation (one-tenth of the whole volume) from each fraction, and the rest of the fraction was incubated with 25 μl binding-control agarose beads (Chromotek) for 1 h at 4 °C to eliminate non-specific binding material. The sample was then incubated with 15 μl of GFP-Trap beads (Chromotek) at 4 °C for 2 h and washed five times with the corresponding (SN1, SN2, or SN3) ice-cold buffer. For the protein–protein interaction experiments, the whole sample of purified material was resuspended in SDS-PAGE sample buffer and analyzed, in parallel with TCL protein samples, by Western blot. For the protein–RNA interaction experiments, only one-fifth of the purified material was resuspended in SDS-PAGE sample buffer and analyzed, in parallel, with total fraction protein samples by Western blot. The rest of the purified material was resuspended in 400 μl of 50 mM sodium acetate plus 10 mM EDTA (pH 5.2) and processed for RNA extraction by the hot-phenol method. After precipitation, the recovered RNA was resuspended in formaldehyde-loading buffer and analyzed by Northern blot in parallel with 4 μg of the TCL RNA samples prepared from the samples taken prior to the GFP-Trap purification step. For mass spectrometry analyses, two samples of GFP-Trap-purified material were used, each of them prepared as described for protein–protein interaction analyses but using 1:2 dilutions of the supernatant fractions to reduce non-specific binding of proteins. The two samples of purified material were pooled and resolved onto SDS-PAGE. Major bands were identified by silver staining, sliced, and subjected to mass spectrometry analysis for their identification following standard procedures at the Genomics and Proteomics Unit of the Centro de Investigación del Cáncer of Salamanca^53^.

### Indirect immunofluorescence and confocal microscopy

For immunofluorescence analyses, cells were fixed in PBS containing 4% paraformaldehyde and permeabilized for 10 min in 0.25 % Triton in TBS-T (20 mM Tris-HCl [pH 7.5], 150 mM NaCl, 0.1% Tween-20). After blocking with 2% bovine serum albumin for 30 min, coverslips were incubated with primary antibody for 2 h at room temperature. Preparations were washed four times with TBS-T, incubated with the secondary antibody for 45 min, and stained with DAPI (4′,6-diamidino-2-phenylindole) before being mounted in Mowiol. Antibodies used for immunofluorescence, and the dilutions used, are shown in Supplementary Table 4. Supplementary Fig. 2a shows representative images of subcellular localization analyses performed in 40–60 cells. Cells endogenously expressing GFP-tagged proteins were fixed, stained with TO-PRO-3 (Thermo), and mounted onto Mowiol prior to microscopy observation. Imaging was performed on a Leica TCS SP8 X confocal microscope, driven by the LAS-X^TM^ version 3.1.5 16308 software, using a ×63/1.4 oil immersion optical lens (HC PL APO SC2) (optical section: 0.896 µm). GFP and TO-PRO-3 samples were excited with a pulsed white light laser at 488 and 641 nm, respectively. GFP images were acquired using a Leica HyD reflected light detector and TO-PRO-3 images with a photomultiplier tube. For each reporter cell line, images were acquired using the same conditions (laser power and detector gain). No manipulations were made other than brightness and contrast adjustments. Images used for quantifications of fluorescence pixel intensities come from samples of the same experiment that were processed and imaged identically. Quantifications of GFP signal intensities were made on 30 cells of each condition in experimental duplicates using ImageJ^TM^. Mann–Whitney test analyses and graphic representations were performed using GraphPad Prism^TM^ version 6.00 for Windows (GraphPad Software).

### Reporting summary

Further information on research design is available in the Nature Research Reporting Summary linked to this article.

## Supplementary information

Supplementary Information

Peer Review

Reporting Summary

## Data Availability

The data that support this study are available from the corresponding author upon reasonable requests. Source data underlying graphs in Figs. 3b, 5b, 7d and in Supplementary Figs. 2b, d and 6a are supplied as a zipped Source Data file. Uncropped versions of blots in Figs. 1b, d, 2a–f, 4a–f, 6a–e, 7a, b and in Supplementary Figs. 2c, 4a–d, i–l, 5, 6b, 7a, b are shown in the Source Data file.

## Source data

Source Data
