# Supplementary material for: Identification of distinct maturation steps involved in human 40S ribosomal subunit biosynthesis
Source: Nat Commun. 2020 Jan 9;11:156. doi: 10.1038/s41467-019-13990-w (PMC6952385; doi:10.1038/s41467-019-13990-w)
Supplement: Supplementary file 1 — Supplementary Information [file 41467_2019_13990_MOESM1_ESM.pdf]

Supplementary Information

Identification of distinct maturation steps involved in human 40S ribosomal subunit biosynthesis

Nieto et al.

Supplementary Fig. 1
Nieto et al.

a

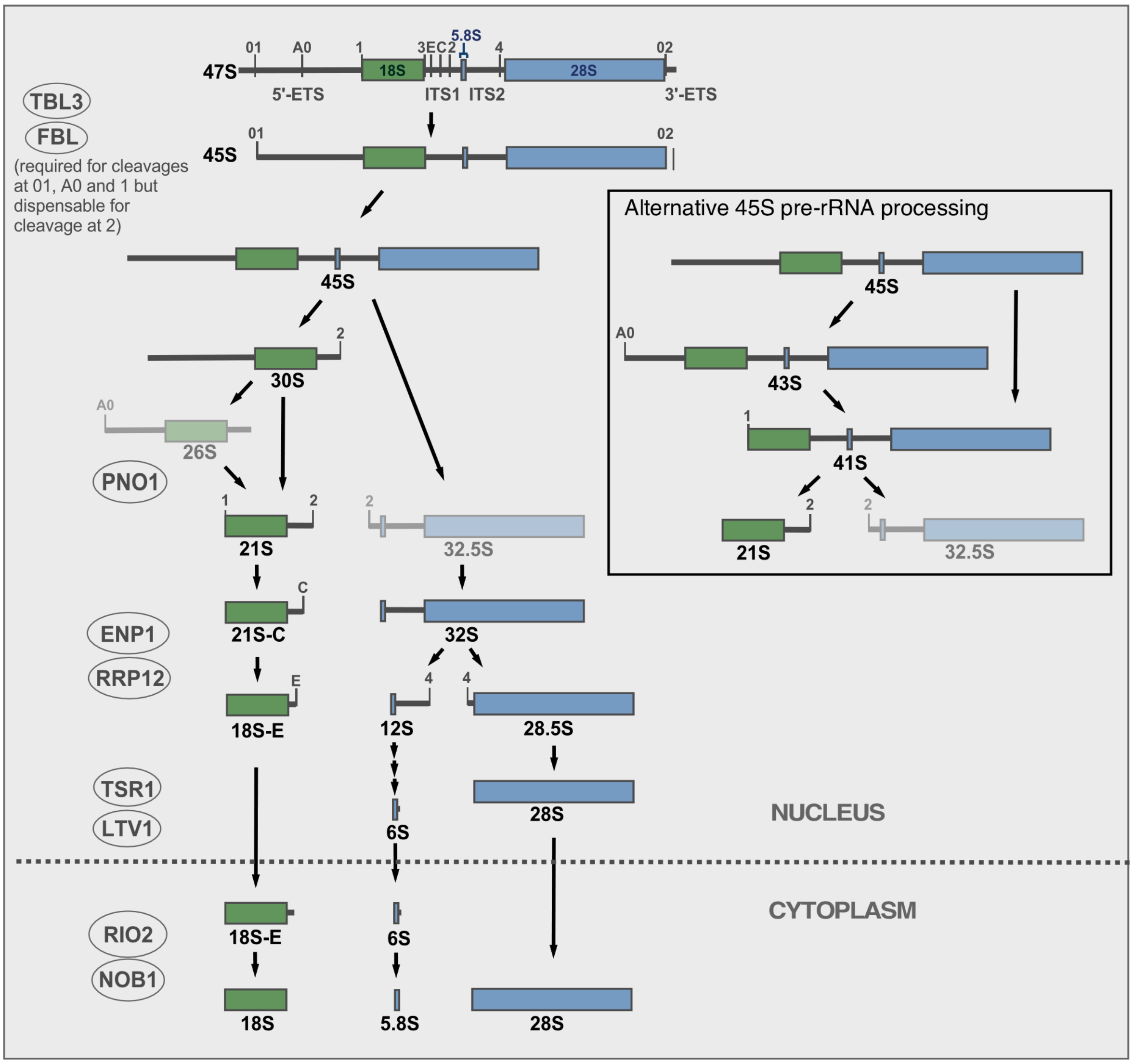

b

Pathway	RBF	Localization	Depletion phenotype (changes in pre-rRNAs contents)	References (PMID)
40S subunit synthesis	TBL3	No	47S↑ 34S↑ 21S↓ 18S-E↓	23439679, 23973377, 22418842
	FBL			
	PNO1	No Nuc Cyt	26S↑ 21S↓ 18S-E↓	19564402, 23973377
	ENP1	No Nuc Cyt	21S↑ 21S-C↑ 18S-E↓	20805244, 23439679
	RRP12			21097556, 23973377
	TSR1	No Nuc Cyt	18S-E↑(nucleus + cytoplasm)	20805244
	LTV1	No Nuc Cyt	21S↑ 21S-C↑ 18S-E↑	19564402, 23973377
	RIO2	Nuc Cyt	18S-E↑(cytoplasm)	16037817, 19564402, 23482395
	NOB1	Nuc Cyt	30S↓ 26S↑ 18S-E↑(cytoplasm)	19564402, 21097556, 28402503
	PARN	No Nuc	18S-E↑(nucleus + cytoplasm)	27899605, 28402503
60S subunit synthesis	PES1	No	41S↑ 12S↓	15225545, 16738141

Supplementary Fig. 1. The pre-rRNA processing steps with participating RBFs. (a) Scheme of the human pre-rRNA processing pathway showing the major intermediate species, the cleavage sites and several 40S RBFs at the steps they are presumed to act on. The 47S primary pre-rRNA contains the 18S, 5.8S and 28S rRNAs flanked by external (5'-ETS and 3'-ETS) and internal (ITS1 and ITS2) RNA segments. This transcript can follow two alternative pathways to produce the 21S and the 32S species. Maturation of the 21S pre-rRNA involves the partial removal of the 3' ITS1 extension by exonucleolytic trimming, giving rise to the 21S-C pre-rRNA. This precursor follows an endonuclease cleavage at site E that yields the 18S-E pre-rRNA. It has long been assumed that the 18S-E species is rapidly exported from the nucleolus to the cytoplasm, where it undergoes final cleavage at site 3 by the endonuclease NOB1. There are no comprehensive analyses on the compositional changes underwent by the nucleolar pre-40S particles upstream of the 18S-E-containing particles. The compositions and structures of late pre-40S particles, which contain 18S-E pre-rRNA, are known. They contain a small set of maturation factors that include NOB1, ENP1, LTV1, TSR1, RIO2, and PNO1. Several factors are indicated on the left at the steps of the pathway they are presumed to act on. The highly-transient 26S and 32.5S pre-rRNAs are shown in faded colors. **(b)** Information summary about the subcellular localization and the depletion-associated pre-rRNA processing phenotypes of the RBFs studied in this work. The nine factors shown in (a) are mostly located in one subcellular compartment, either the nucleolus (No), the nucleoplasm (Nuc) or the cytoplasm (Cyt). The compartment of predominant localization is written in bold large letters. The pre-rRNA species accumulated or decreased upon depletion of the RBF are indicated by arrows pointing up or down, respectively. The assignment of the RBFs to the specific steps shown in (a) is inferred from the function of the homologous protein in yeast, the accumulation of pre-rRNA intermediates and/or the delocalization pattern of 40S synthesis reporters upon depletion of the RBF in human cells. The table also includes information about PES1 and PARN, two factors not shown in (a) that were analyzed in some experiments of this study. The 34S pre-rRNA (not depicted in **a**) contains the 18S rRNA sequence flaked by the complete 5'-ETS region and the segment of ITS-1 encompassed by the 3 and 2 sites. This species is accumulated upon depletion of TBL3 or FBL.

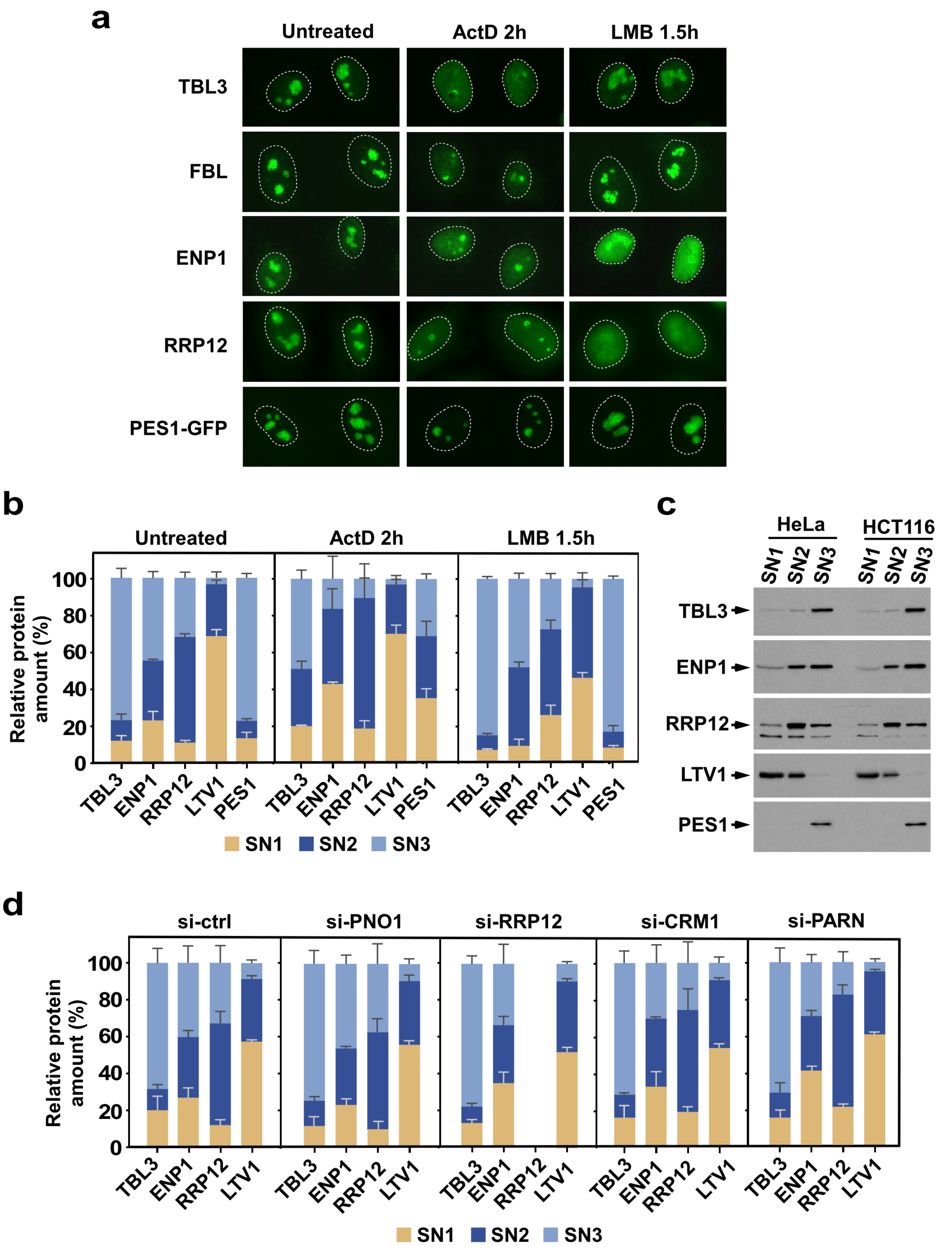
a
Untreated
ActD 2h
LMB 1.5h
TBL3
FBL
ENP1
RRP12
PES1-GFP
b
Untreated
ActD 2h
LMB 1.5h
Relative protein amount (%)
0
20
40
60
80
100
TBL3
ENP1
RRP12
LTV1
PES1
SN1
SN2
SN3
c
HeLa
HCT116
SN1
SN2
SN3
TBL3
ENP1
RRP12
LTV1
PES1
d
si-ctrl
si-PNO1
si-RRP12
si-CRM1
si-PARN
Relative protein amount (%)
TBL3
ENP1
RRP12
LTV1
SN1
SN2
SN3

Supplementary Fig. 2. Fractionation of preribosomal components with the PSE method. (a) Subcellular localization of the indicated RBFs revealed by immunofluorescence analyses of HeLa cells (top four panels) and fluorescence microscopy of HeLa•*PES1-GFP* cells untreated or treated with ActD or LMB for the indicated times. The area of the cell nucleus is indicated by a dashed line. Previous studies also reported the localization of these proteins in the nucleolus (see summary information in Supplementary Fig. 1b). **(b)** Relative contents of the indicated RBFs in PSE fractions (SN1, SN2 and SN3) prepared from HeLa cells untreated and treated with ActD or LMB. Data are the mean and ± s.d. quantifications of Western blots from experimental triplicates (one of them shown in Fig. 1b). **(c)** PSE fractionation profiles of several RBFs in HeLa and HCT116 cells. PSE extracts were prepared from exponentially-growing HeLa and HCT116 cells, and the relative contents of the indicated RBFs were analyzed by Western blot. **(d)** Relative contents of four RBFs in PSE fractions prepared from HeLa cells transfected with the indicated si-RNAs for 48 hours, except in the case of si-PARN that was analyzed at 72 hours. si-ctrl: control si-RNA. Data are the mean and ± s.d. quantifications of Western blots from experimental triplicates (two of them shown in Figs. 4b and Supplementary Fig. 7b). Source data are provided as a Source Data file.

Supplementary Fig. 3
Nieto et al.

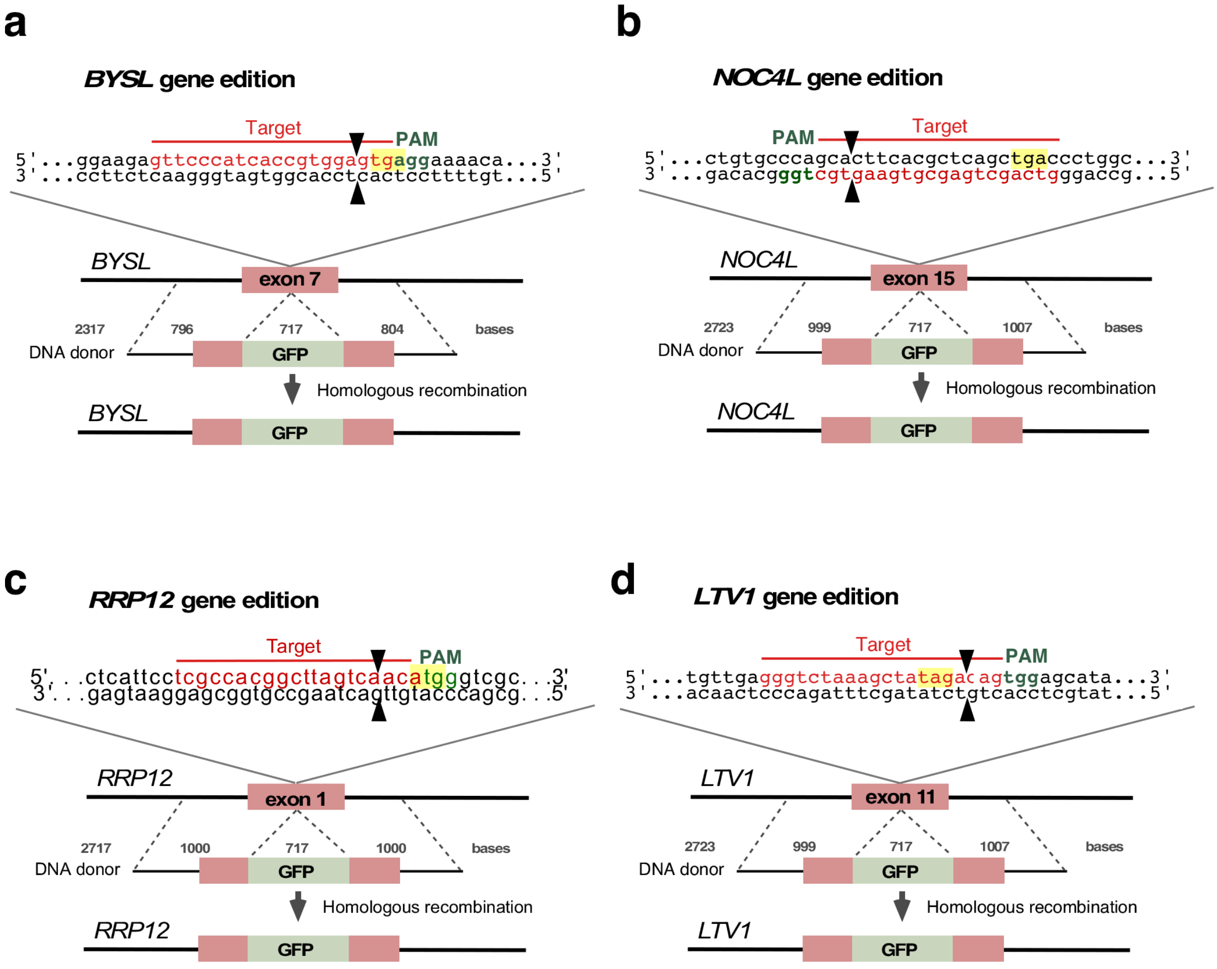

Supplementary Fig. 3. Generation of cell lines that endogenously express GFP-tagged versions of four RBFs. (a-d) Scheme showing the CRISPR/Cas9 strategies used to edit the *BYSL*, *NOC4L*, *RRP12* and *LTV1* genes. The sequence targeted by the gRNA (in red), the protospacer adjacent motif (PAM) sequence (in green), the STOP or START codons (in yellow), the cleavage site (arrows) and the resulting recombination product are shown. Sizes (bp) of the GFP sequence, left arm and right arm in the recombination donor cassette are also indicated.

Supplementary Fig. 4
Nieto et al.

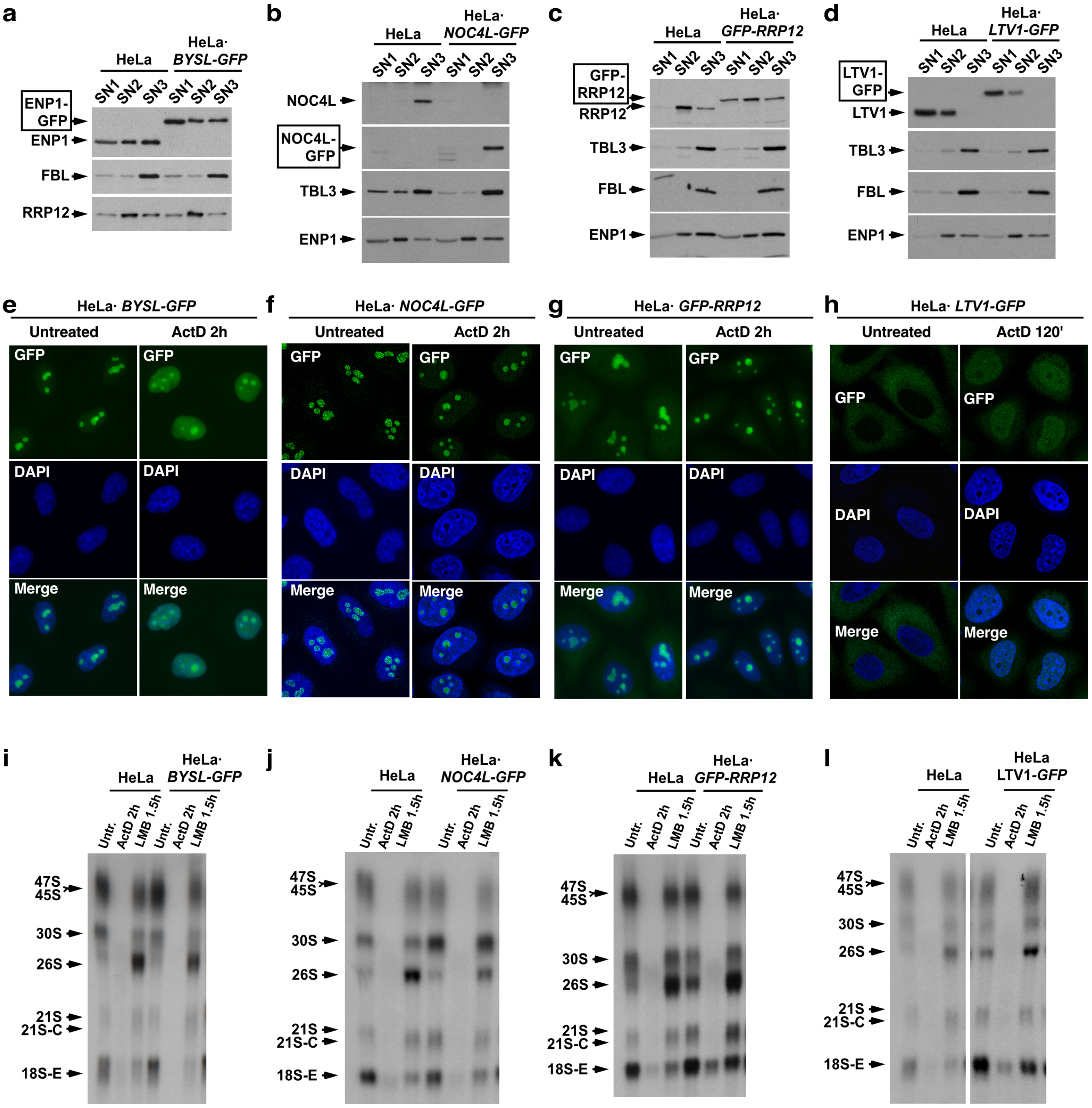

Supplementary Fig. 4. Functionality of GFP-tagged versions of RBFs in the absence of the corresponding wild type proteins. (a-d) PSE fractionation profiles of the indicated proteins in HeLa-derived cell clones in which all copies of one individual gene (*BYSL*, *NOC4L*, *RRP12* or *LTV1*) have been edited to express a GFP-fused version of the corresponding protein as indicated in Supplementary Fig. 3. The fractionation profiles of the normal proteins in non-modified HeLa cells are shown in the first three lanes. Each Western blot was incubated with antibodies for the protein of interest and for early (FBL, TBL3) or intermediate (RRP12, ENP1) RBFs. Incubation with antibodies to GFP is also shown in the case of NOC4L-GFP because the epitope recognized by the NOC4L antibody became masked in the NOC4L-GFP fusion (b). **(e-h)** Subcellular distributions of RBFs in the corresponding gene-edited homozygous HeLa cell clones untreated and treated with ActD for 2 h. ENP1-GFP, NOC4L-GFP and GFP-RRP12 remain in the nucleolar interior, excluded from nucleolar caps, upon ActD treatment. This is consistent with their presence in maturing preribosomes localized in the nucleolar granular component. **(i-l)** 18S pre-rRNA processing profiles of normal HeLa cells and the indicated gene-edited HeLa clones untreated and treated with ActD or LMB for the indicated times. Some differences are observed in the properties of ENP1-GFP and GFP-RRP12 proteins as compared to the normal counterparts. Both of them are relatively enriched in the SN1 supernatant (panels a and c) and, in the case of GFP-RRP12, it causes an accumulation of the 26S pre-rRNA (panel k). These differences might be due to changes in the turnover/recycling rates of the proteins in the cytoplasm and/or a delayed import in the nucleus. Such changes do not have a major impact in the production of 40S subunits, as indicated by the normal growth and normal 28S/18S ratios exhibited by the cells. Several experiments shown in the main text confirmed that both GFP-fused proteins are efficiently incorporated onto pre-40S-No1 and No2 complexes (Figs. 2c -2f, 6a, 6b, and Supplem. Fig. 5).

Supplementary Fig. 5
Nieto et al.

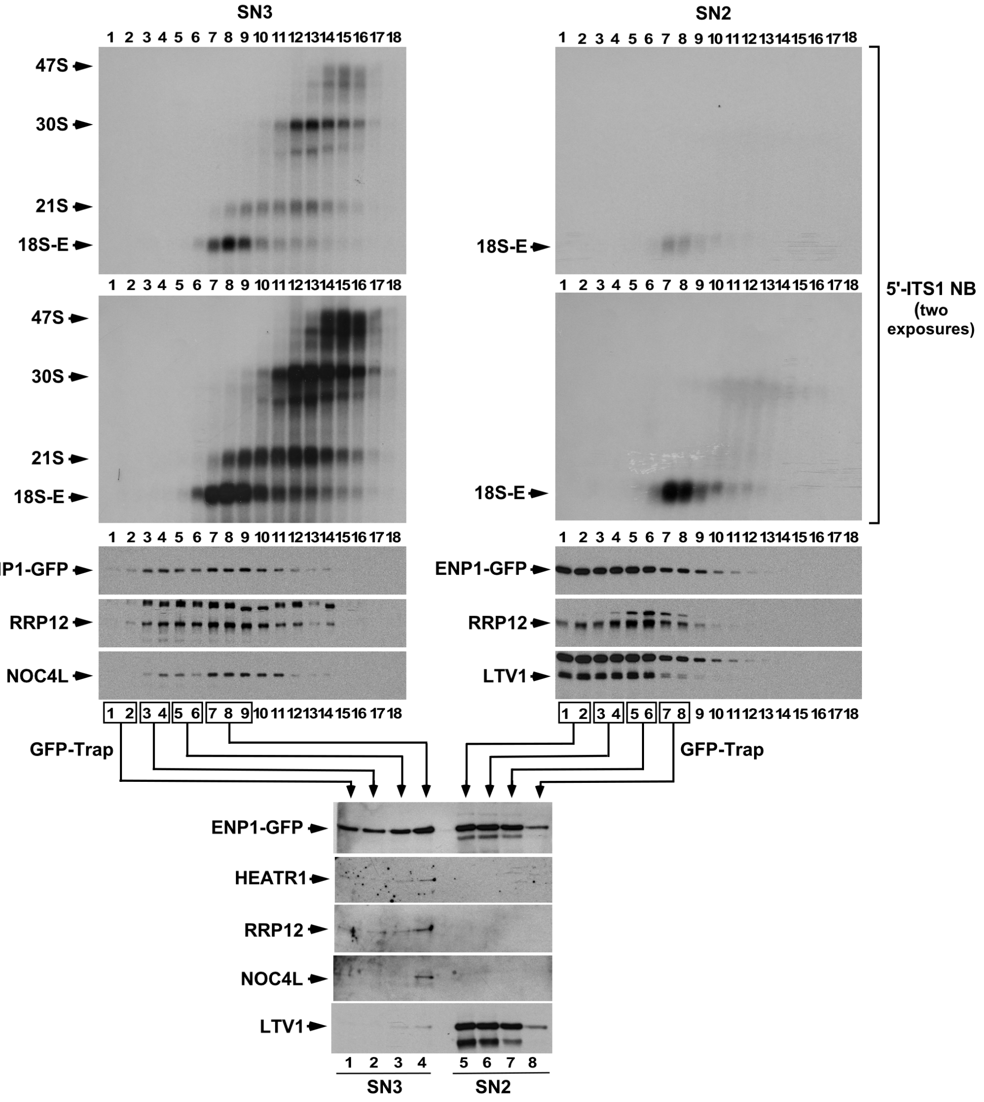

Supplementary Fig. 5. The PSE method allows the separation of two distinct ENP1-containing pre-40S intermediates. Sedimentation profiles of preribosome species in the SN3 (top panels on the left) and SN2 (top panels on the right) supernatants obtained with the PSE method, and differential detection of RBFs in ENP1-GFP-containing complexes (bottom set of panels). PSE extracts were prepared from HeLa•*BYSL-GFP* cells and subjected to sucrose gradient sedimentation analyses. The contents of pre-rRNA species, ENP1-GFP, RRP12 and NOC4L in each fraction of the gradient were detected by Northern blot and Western blot. The fractions corresponding to the top half of the gradient were combined into four pools. The fourth pool included the fractions enriched for the 18S-E pre-rRNA (fractions 7-8-9 in the SN3 gradient and fractions 7-8 in the SN2 gradient). ENP1-GFP was purified from the four fraction pools using GFP-Trap, and co-purification of HEATR1, RRP12, NOC4L and LTV1 was assessed by Western blot. In this experiment the SN3 and SN2 supernatant samples were prepared from the same cells, and sucrose gradient fractionations and GFP-Trap purifications were performed in parallel. The side-by-side Northern and Western blot images correspond to the same exposures. The pre-40S particles in the SN2 supernatant tend to be disrupted upon extraction, as indicated by the presence of a large proportion of ENP1-GFP, RRP12 and LTV1 in the upper fractions of the gradient (top panels on the right, fractions 1-6).

Supplementary Fig. 6
Nieto et al.

a

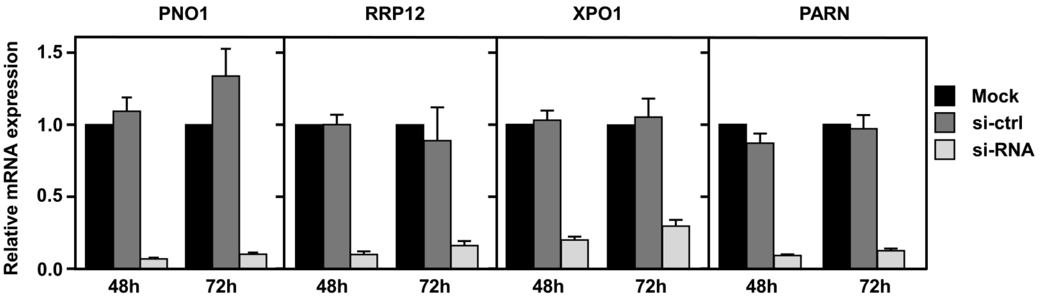

b

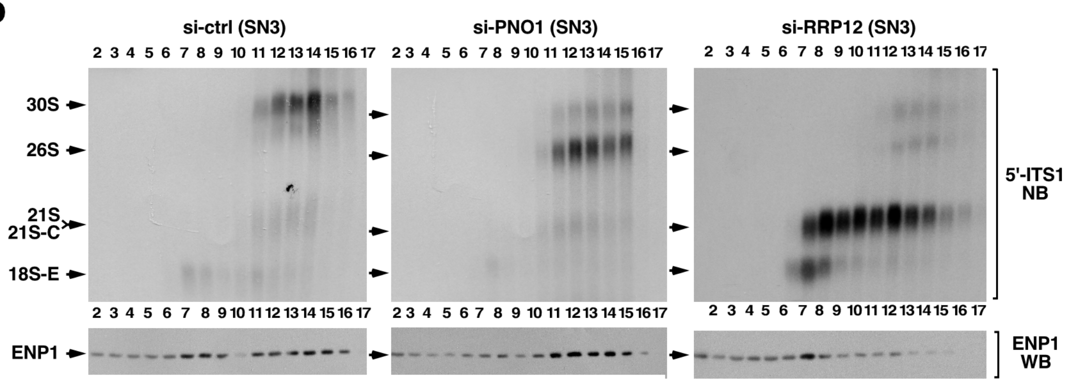

Supplementary Fig. 6. Aberrant accumulation of pre-40S complexes in the absence of PNO1 or RRP12. (a) Efficiencies of the gene-expression knockdown conditions used in this work. Results of qPCR analyses showing the contents of *PNO1*, *RRP12*, *CRM1 (XPO1)* and *PARN* mRNAs in HeLa cells transfected with either a control siRNA (si-ctrl) or with the corresponding specific si-RNA. Cells were harvested at 48 hours and 72 hours after transfection. mRNA contents were calculated relative to those obtained in mock-transfected cells, which were given the arbitrary value of 1. Error bars represent the s.e.m. values. **(b)** Aberrant accumulation of 26S pre-rRNA- and 21S pre-rRNA-containing particles in the absence of PNO1 and RRP12, respectively. PSE extracts were prepared from HeLa cells transfected with the indicated siRNAs for 48 hours and the SN3 fraction was subjected to sucrose gradient sedimentation analyses. The contents of the pre-rRNA species and ENP1 protein in each fraction were detected by Northern blot using the 5'-ITS1 probe and Western blot with an anti-ENP1 antibody, respectively. Source data are provided as a Source Data file.

Supplementary Fig. 7
Nieto et al.

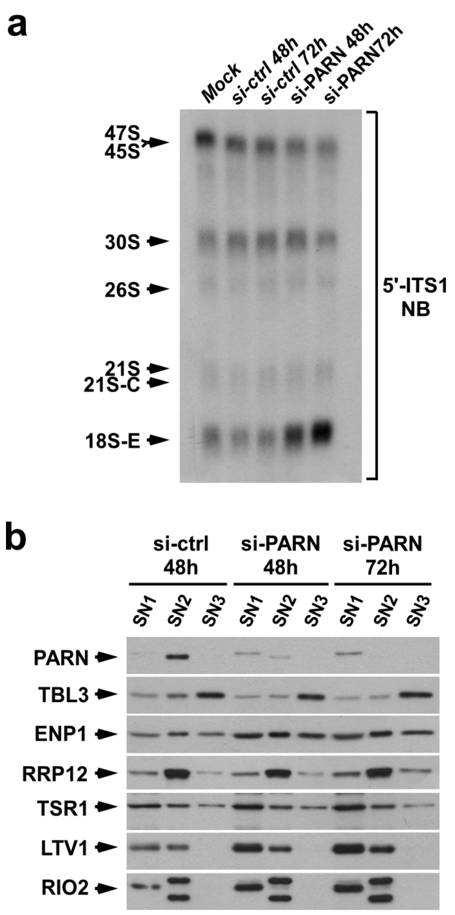

Supplementary Fig. 7. PARN depletion induces an abnormal accumulation of 40S late-maturation factors outside the nucleolus. (a) Relative contents of pre-rRNA processing species in HeLa cells transfected with the indicated siRNAs that were harvested at the indicated times after transfection. The 5'-ITS1 probe was used for Northern blot analyses of total RNAs prepared with the Trizol method. **(b)** Relative contents of several RBFs in the SN1, SN2 and SN3 fractions obtained with the PSE method from HeLa cells transfected with the indicated siRNAs and harvested at the indicated times after transfection.

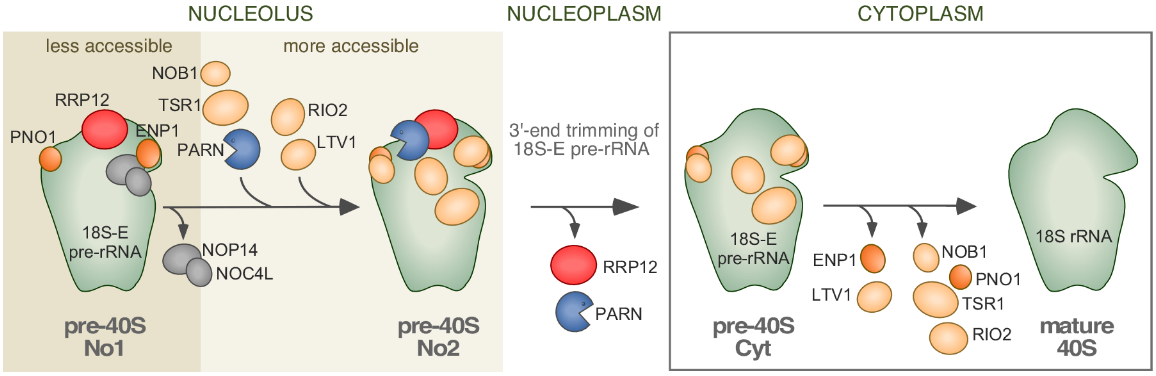

Supplementary Fig. 8. Model of the maturation of the human 40S ribosomal subunit. The initial pre-40S particles emerge in a low-solubility subcompartment of the nucleolus (No1 phase), and then enter a maturation phase that takes place in more-accessible nucleolar sites. During this second stage (No2 phase) pre-40S particles incorporate PARN and a subset of late maturation RBFs. After leaving the nucleolus, the particles undergo a PARN/RRP12-mediated event that renders the late precursors to be matured in the cytoplasm (Cyt). It was previously described that the 18S-E pre-rRNA species, which is formed upon emergence of pre-40S No1 particles, undergoes a 3' to 5' PARN-mediated trimming inside the nucleoplasm (Montellese et al., 2017). Based on the order of compositional changes established here, such a trimming process must be happening just before or at the time of RRP12 release. No equivalent step, mediated by a ribonuclease such as PARN, is found in yeast. The very final pre-rRNA processing step, the endonucleolytic cleavage at site 3, occurs in the cytoplasm. In this manuscript we unveil the segregation of the emerging (No1) and maturing (No2) particles inside the nucleolus, the differences in composition and structural stability between the two nucleolar stages, the positioning of the step mediated by RRP12, and the involvement of PARN in such step (see main text for more details). The presence of PNO1 in all pre-40S intermediates is based on data from previously-published work by other groups (Ameismeier et al., 2018; Larburu et al., 2016; Zemp et al., 2009). PNO1 was not seen by silver staining as a major component of the ENP1- or RRP12-containing complexes purified in this study, indicating that its association is weak, sensitive to the extraction procedures followed here, or masked by other proteins present in the purifications. The stepwise release of late maturation factors in the cytoplasm has been described by others (Ameismeier et al., 2018).

Supplementary Table 1. Plasmids used in this study

Name	Description	Use	Reference
pX330	pX330-U6-Chimeric_BB-CBh-hSpCas9	Generation of Cas9/sg plasmids	Feng Zhang generous deposit at Addgene (plasmid # 42230)
pBN72	pX330-sg4CBYSL	GFP knock-in at ENP1 C-terminus (Cas9/sg plasmid)	This study
pBN79	pX330-sg2NRRP12	GFP knock-in at RRP12 N-terminus (Cas9/sg plasmid)	This study
pBN81	pBluescript-gRRP12(Chr. X: 97402208-97401232)-GFP-gRRP12(Chr. X: 97401231-97400232)	GFP knock-in at RRP12 N-terminus (HDR donor plasmid)	This study
pBN83	pX330-sg2CLTV1	GFP knock-in at LTV1 C-terminus (Cas9/sg plasmid)	This study
pBN84	pX330-sg1CNOC4L	GFP knock-in at NOC4L C-terminus (Cas9/sg plasmid)	This study
pBYSL-GFP-BYSL	pMK-RQ-gBYSL(Chr. VI: 41931907-41932703)-GFP-gBYSL(Chr. VI: 41932704-41933506)	GFP knock-in at ENP1 C-terminus (HDR donor plasmid)	This study (GeneArt, Invitrogen)
pGH1	pBlluescript-gLTV1(Chr. VI: 143862525-143863524)-GFP-gLTV1L(Chr. VI: 143863525-143864528)	GFP knock-in at LTV1 C-terminus (HDR donor plasmid)	This study
pNOC4L-HDR	pMA-RQ-gNOC4L(Chr. XII: 132151399-132152398)-GFP-gNOC4L(Chr. XII: 132152399-132153398)	GFP knock-in at NOC4L C-terminus (HDR donor plasmid)	This study (GeneArt, Invitrogen)

Supplementary Table 2. Oligonucleotides used in this study

No	Name	Sequence	Use
1	sgCBYSL F4	CACCGGTTCCCATCACCGTGGAGTG	Generation of Cas9/sg plasmid for *BYSL* edition
2	sgCBYSL R4	AAACCACTCCACGGTGATGGGAACC	Generation of Cas9/sg plasmid for *BYSL* edition
3	sgCNOC4L F1	CACCGGTCAGCTGAGCGTGAAGTGC	Generation of Cas9/sg plasmid for *NOC4L* edition
4	sgCNOC4L R1	AAACGCACTTCACGCTCAGCTGACC	Generation of Cas9/sg plasmid for *NOC4L* edition
5	sgNRRP12 F2	CACCGTCGCCACGGCTTAGTCAACA	Generation of Cas9/sg plasmid for *RRP12* edition
6	sgNRRP12 R2	AAACTGTTGACTAAGCCGTGGCGAC	Generation of Cas9/sg plasmid for *RRP12* edition
7	gRRP12 F2	GCCACTCGAGCTATGGGTCGCTCGGGAAAGTTGCC	Generation of HDR donor for *RRP12* edition
8	gRRP12 R2	CCAATGAAGCTTGGCCAGAGCTGAAGAAAAGGC	Generation of HDR donor for *RRP12* edition
9	gRRP12 F1-2	GCCAGCTAGCGAGTGCAATGGCGTGATCTCAGCTCACCGC	Generation of HDR donor for *RRP12* edition
10	gRRP12 R1-2	CCAATGACCGGTAGGTTGACTAAGCCGTGGCGAGGAATGAGCTTAAATGACCGGC	Generation of HDR donor for *RRP12* edition
11	sgCLTV1 F2	CACCGGGGTCTAAAGCTATAGACAG	Generation of Cas9/sg plasmid for *LTV1* edition
12	sgCLTV1 R2	AAACCTGTCTATAGCTTTAGACCCC	Generation of Cas9/sg plasmid for *LTV1* edition
13	gLTV1 F2	CTGACATCTCGAGCTTAGACAGTGGAGCATACAGGGCAAGGC	Generation of HDR donor for *LTV1* edition
14	gLTV1 R2	CCAATGGGTACCTCAGAGCAACAGTGTTCACAGATTT	Generation of HDR donor for *LTV1* edition
15	5'-ITS1	CCTCGCCCTCCGGGCTCCGGGCTCCGTTAATGATC	Northern blot
16	ITS2	CTGCGAGGGAACCCCCAGCCGCGCA	Northern blot
17	U3	ACCACTCAGACCGCGTTCTCTCC	Northern blot

Supplementary Table 3. siRNAs used in this study

siRNA	Gene Symbol	Gene name	Gene ID	siRNA ID	Sense siRNA Sequence	Source/Reference
PARN	*PARN*	Poly(A)-specific ribonuclease	5073		GAGCUCUGUCCUAUGUAUCUCCUAA	Invitrogen stealth siRNA
PNO1	*PNO1*	Partner of Nob1 homolog (S. cerevisiae)	56902	s32351	CAGUCCCAGCUAACAGAUAtt	Ambion / #4392420
RRP12	*RRP12*	Ribosomal RNA processing 12 homolog (S.cerevisiae)	23223	s23326	GCAUCCAGGAGAUUGAGAAtt	Ambion / #4392420
XPO1	*XPO1*	Exportin 1 (CRM1 homolog, yeast)	7514	s14937	AUCUAAUAAGUUGAUAUCCag	Ambion / #4390824

Supplementary Table 4. Antibodies used in this study

Antibody	Raised in	Use and Dilution	Source / Reference
ENP1	Rabbit	Western blot 1:1000<br>Immunofluorescence 1:400	Bethyl / A304-568A
Fibrillarin	Mouse	Immunofluorescence 1:500	EnCor / MCA-38F3
Fibrillarin	Rabbit	Western blot 1:200	Santa Cruz (H-140) / sc-25397
GFP	Mouse	Western blot 1:1000	Clontech / 632381
Histone H3	Rabbit	Western blot 1:5000	Abcam / ab1791
LTV1	Rabbit	Western blot 1:1000	Sigma / HPA030161
Mouse Alexa488	Donkey	Immunofluorescence 1:500	Invitrogen / A21202
Nucleophosmin/B23	Mouse	Western blot 1:2000	Invitrogen / 32-5200
NOB1	Rabbit	Western blot 1:1000	Novus Biologicals / NBP2-19558
NOC4L	Rabbit	Western blot 1:1000	Novus Biologicals / NBP1-80464
PARN	Rabbit	Western blot 1:1000	Abcam / ab27778
PCNA	Mouse	Western blot 1:2000	Abcam (PC10) / ab29
PES1	Mouse	Western blot 1:100	Santa Cruz (H-10) / sc-166300
PNO1	Rabbit	Western blot 1:100	Santa Cruz (K-14) / sc-133265
Rabbit Alexa488	Donkey	Immunofluorescence 1:500	Life Technologies / A21206
RIOK2	Rabbit	Western blot 1:1000	Novus Biologicals / NBP1-30098
RRP12	Rabbit	Immunofluorescence 1:200	Novus Biologicals / NBP2-15105
RRP12	Mouse	Western blot 1:1000	Santa Cruz (A-3) / sc-398593
TBL3	Rabbit	Western blot 1:1000	Sigma / HPA042562
TSR1	Rabbit	Western blot 1:1000	Bethyl / A305-112A
Tubulin	Mouse	Western blot 1:2000	Calbiochem / CP06
