# Supplementary material for: Identification of distinct maturation steps involved in human 40S ribosomal subunit biosynthesis
Source: Nat Commun. 2020 Jan 9;11:156. doi: 10.1038/s41467-019-13990-w (PMC6952385; doi:10.1038/s41467-019-13990-w)
Supplement: Supplementary file 3 — Reporting Summary [file 41467_2019_13990_MOESM3_ESM.pdf]

natureresearch

Corresponding author(s): Mercedes Dosil

Reporting Summary

Nature Research wishes to improve the reproducibility of the work that we publish. This form provides structure for consistency and transparency in reporting. For further information on Nature Research policies, see Authors & Referees and the Editorial Policy Checklist.

Statistical parameters

When statistical analyses are reported, confirm that the following items are present in the relevant location (e.g. figure legend, table legend, main text, or Methods section).

n/a	Confirmed	
☐	☒	The exact sample size (*n*) for each experimental group/condition, given as a discrete number and unit of measurement
☐	☒	An indication of whether measurements were taken from distinct samples or whether the same sample was measured repeatedly
☐	☒	The statistical test(s) used AND whether they are one- or two-sided<br>*Only common tests should be described solely by name; describe more complex techniques in the Methods section.*
☒	☐	A description of all covariates tested
☒	☐	A description of any assumptions or corrections, such as tests of normality and adjustment for multiple comparisons
☒	☐	A full description of the statistics including central tendency (e.g. means) or other basic estimates (e.g. regression coefficient) AND variation (e.g. standard deviation) or associated estimates of uncertainty (e.g. confidence intervals)
☒	☐	For null hypothesis testing, the test statistic (e.g. *F*, *t*, *r*) with confidence intervals, effect sizes, degrees of freedom and *P* value noted<br>*Give P values as exact values whenever suitable.*
☒	☐	For Bayesian analysis, information on the choice of priors and Markov chain Monte Carlo settings
☒	☐	For hierarchical and complex designs, identification of the appropriate level for tests and full reporting of outcomes
☒	☐	Estimates of effect sizes (e.g. Cohen's *d*, Pearson's *r*), indicating how they were calculated
☐	☒	Clearly defined error bars<br>*State explicitly what error bars represent (e.g. SD, SE, CI)*

Our web collection on statistics for biologists may be useful.

Software and code

Policy information about availability of computer code

Data collection	Software used for data collection: NanoDrop 1000 3.8.1 (Thermo Scientific); LAS-X 3.1.5 (Leica); Metamorph 7.6.2.0 (MDS Analytical Technologies); FACSDiva 7.0 (BD); Flex Control 3.0 (Bruker Daltonics)
Data analysis	Software used for data analysis: Excel 2010 (Microsoft); Image J 1.46r (NIH); GraphPad Prism 6.0 (GraphPad); Flex Analysis 3.0 (Bruker Daltonics); MASCOT 2.3.2 (Matrix Science)

For manuscripts utilizing custom algorithms or software that are central to the research but not yet described in published literature, software must be made available to editors/reviewers upon request. We strongly encourage code deposition in a community repository (e.g. GitHub). See the Nature Research guidelines for submitting code & software for further information.

Data

Policy information about availability of data

All manuscripts must include a data availability statement. This statement should provide the following information, where applicable:

- Accession codes, unique identifiers, or web links for publicly available datasets
- A list of figures that have associated raw data
- A description of any restrictions on data availability

All raw data (uncropped blots and data used to generate quantification graphs) associated to all figures are provided in a Source Data file.

April 2018

Field-specific reporting

Please select the best fit for your research. If you are not sure, read the appropriate sections before making your selection.

☒ Life sciences ☐ Behavioural & social sciences ☐ Ecological, evolutionary & environmental sciences

For a reference copy of the document with all sections, see nature.com/authors/policies/ReportingSummary-flat.pdf

Life sciences study design

All studies must disclose on these points even when the disclosure is negative.

Sample size	Sample-size calculations not employed. Sample size chosen for each experiment included a number of control and problem samples adequate to render consistent and comparable data. All samples within a experiment were treated, processed and analyzed side-by-side.
Data exclusions	No data were excluded
Replication	All experiments were reproduced. Results were similar in all replicates. Microscopy, protein-complex mass-spectrometry, and total-lysate Western and Northern blot analyses were repeated a minimum of three times. Sucrose-gradient sedimentation and GFP-Trap co-precipitation analyses were repeated a minimum of two times.
Randomization	Not apllicable in this study
Blinding	Not applicable in this study

Reporting for specific materials, systems and methods

Materials & experimental systems

n/a	Involved in the study
☐	☒ Unique biological materials
☐	☒ Antibodies
☐	☒ Eukaryotic cell lines
☒	☐ Palaeontology
☒	☐ Animals and other organisms
☒	☐ Human research participants

Methods

n/a	Involved in the study
☒	☐ ChIP-seq
☒	☐ Flow cytometry
☒	☐ MRI-based neuroimaging

Unique biological materials

Policy information about availability of materials

Obtaining unique materials	The four GFP knock-in HeLa-derived cell lines generated in this study are fully available upon request. All gRNA/Cas9 and HDR template plasmids used to edit the genes of ribosome biogenesis factors are fully available upon request. The rest of materials are available from commercial sources as indicated in the Methods section.

Antibodies

Antibodies used	All primary antibodies and corresponding providers are listed in Supplemental Table 4.
Validation	Primary antibodies used in Western blots were validated on the basis of (1) correspondence to protein bands identified by mass spectrometry, (2) expected fractionation of the recognized protein in specific subcellular fractions and/or (3) diminished signal upon specific siRNA-mediated gene silencing. Primary antibodies used in immunofluorescence studies were validated on the basis of expected subcellular localization and specific de-localization pattern upon treatment of cells with actinomycin D or leptomycin B.

Eukaryotic cell lines

Policy information about cell lines

Cell line source(s)	HeLa (ATCC); HCT116 kindly provided by prof. María Sacristán, Centro de Investigación del Cáncer, Salamanca, Spain
Authentication	STR method
Mycoplasma contamination	All cell lines were tested for mycoplasm contamination
Commonly misidentified lines (See ICLAC register)	None of the used cell lines is listed in ICLAC database
