# Supplementary material for: Identification of distinct maturation steps involved in human 40S ribosomal subunit biosynthesis
Source: Nat Commun. 2020 Jan 9;11:156. doi: 10.1038/s41467-019-13990-w (PMC6952385; doi:10.1038/s41467-019-13990-w)

FIGURE 1b

α-TBL3

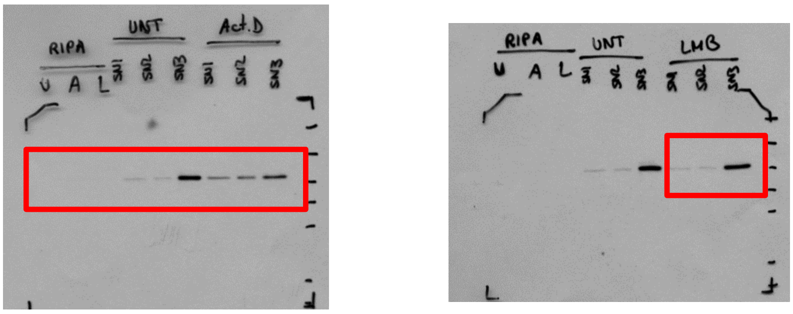

α-FBL

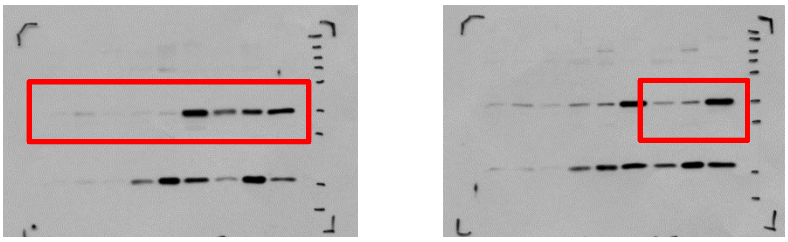

α-ENP1

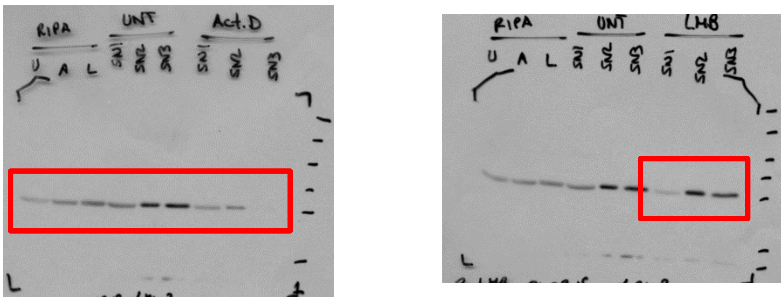

α-RRP12

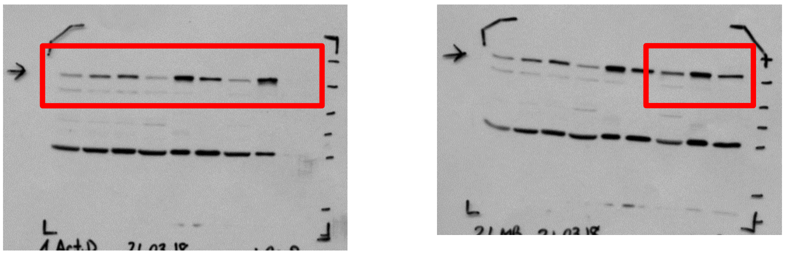

α-TSR1

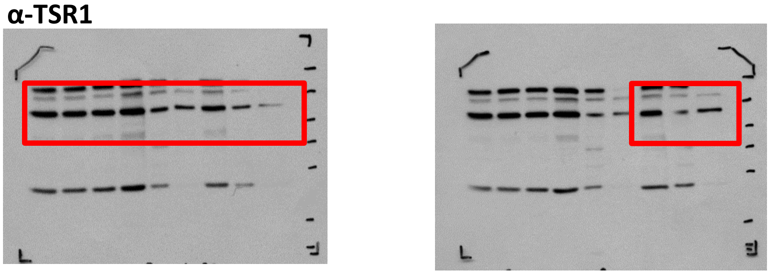

α-LTV1

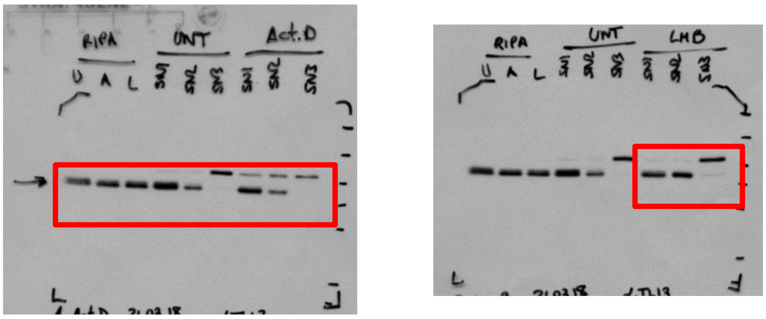

FIGURE 1b (continued)

α-RIO2

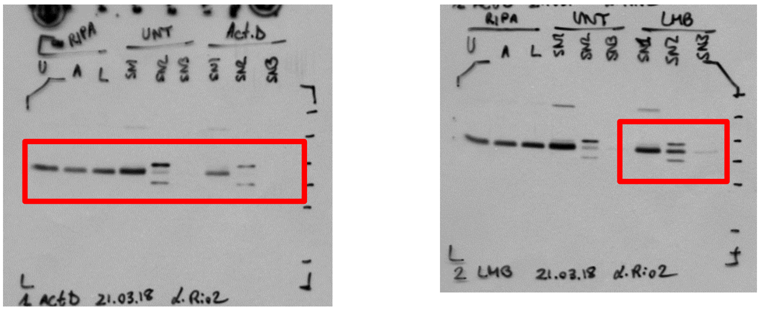

α-NOB1

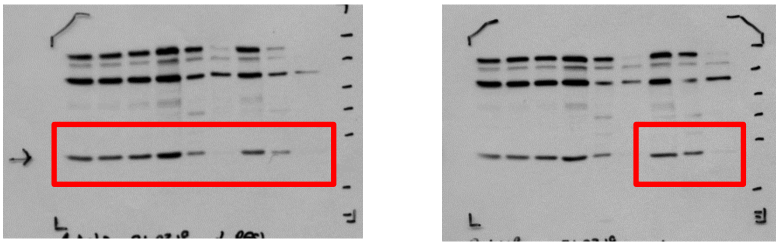

α-PES1

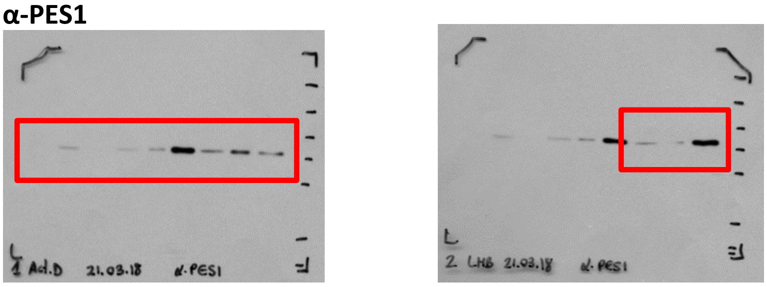

α-Tubulin

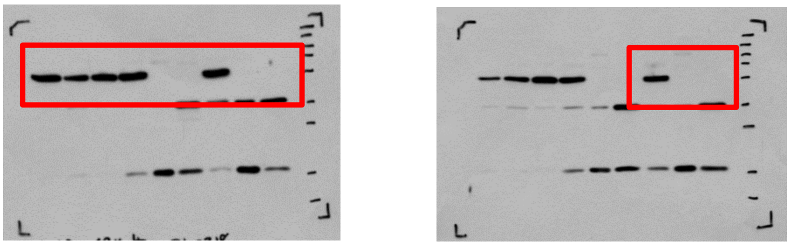

α-PCNA

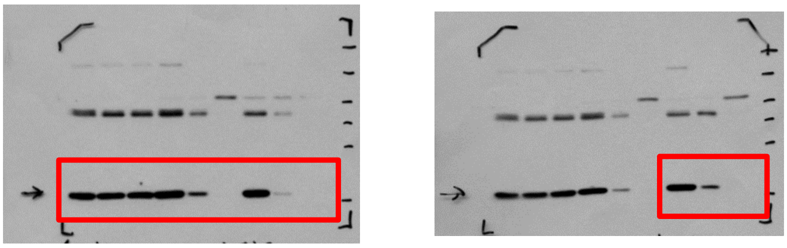

α-H3

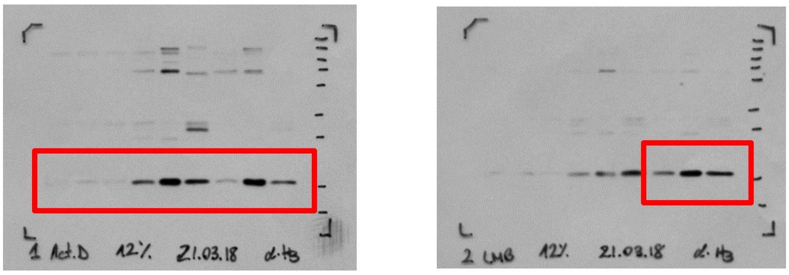

FIGURE 1d

5’-ITS1

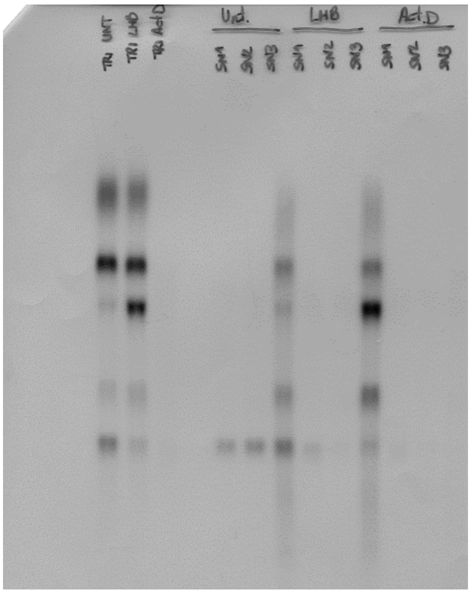

U3

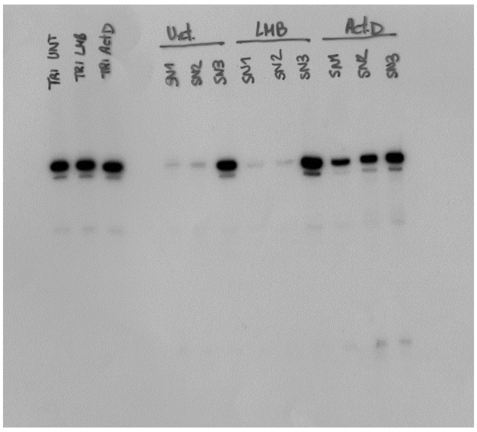

ITS2

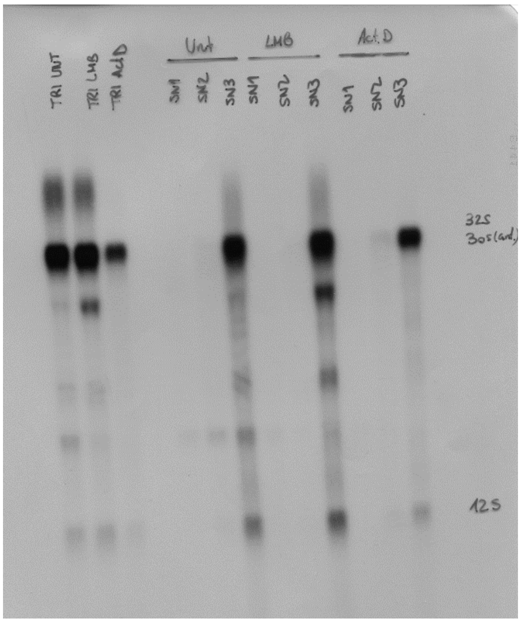

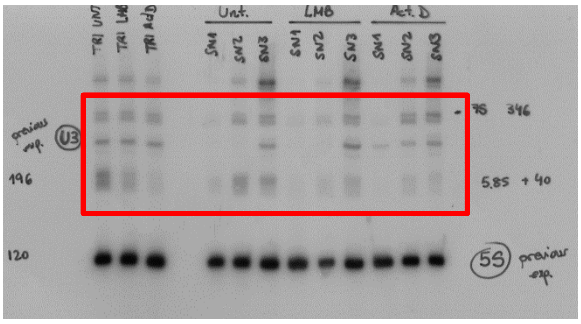

FIGURE 2a

5'-ITS1

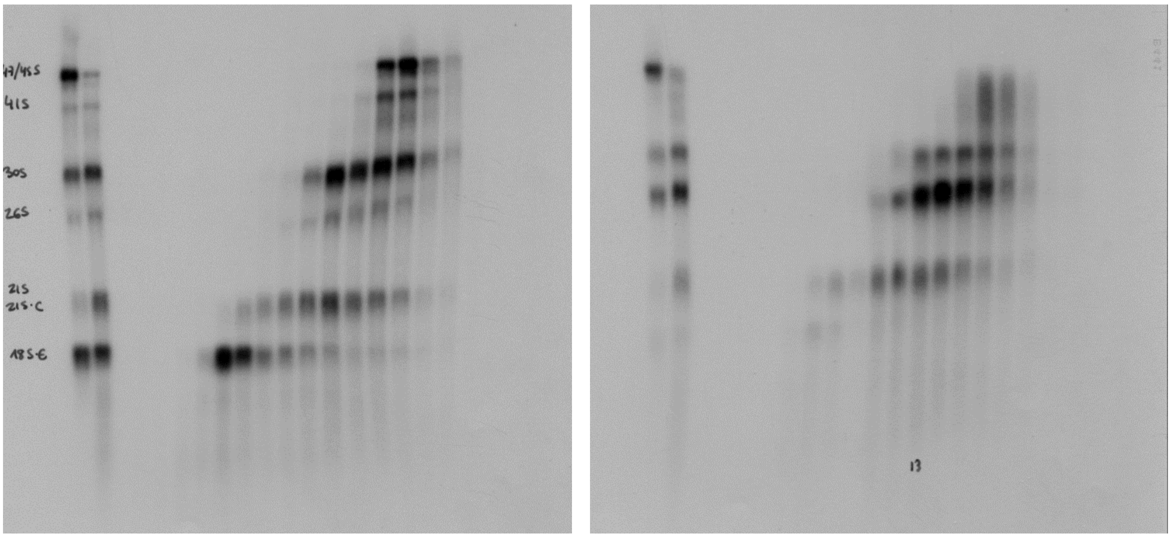

ITS2

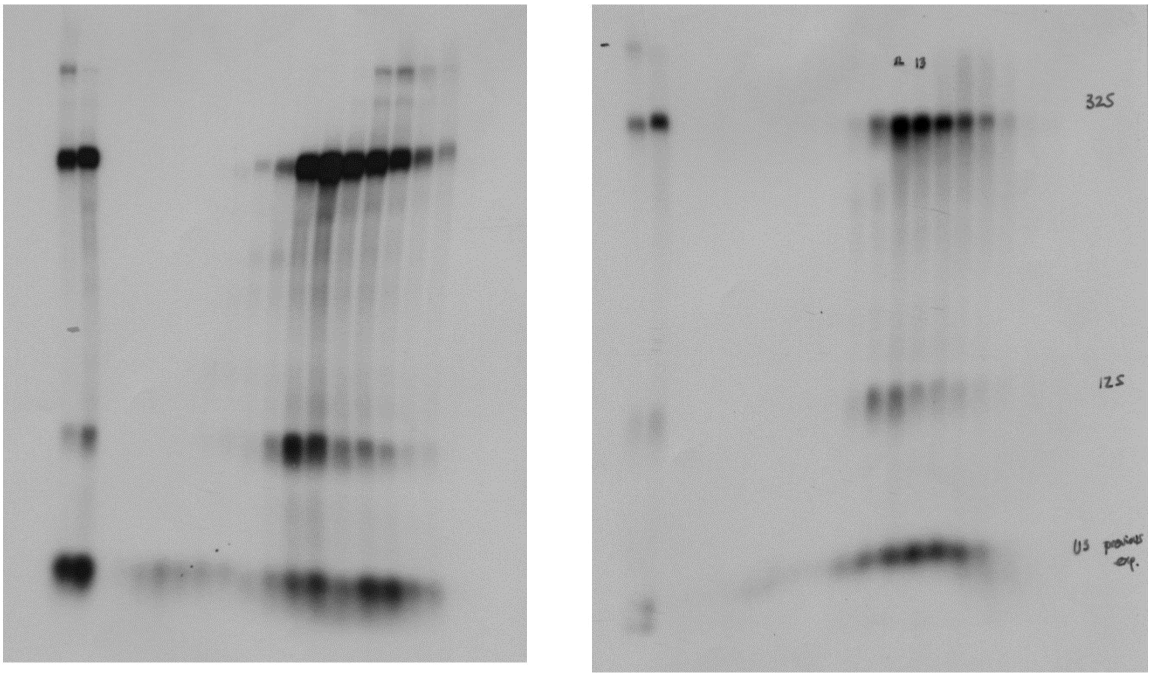

α-TBL3

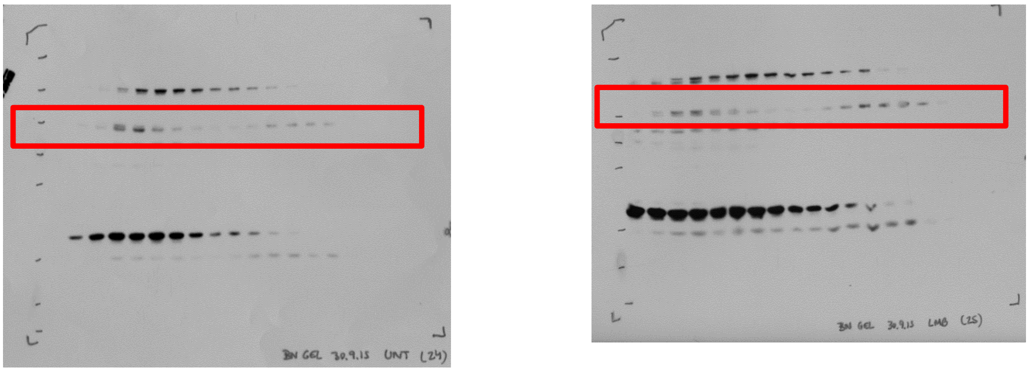

FIGURE 2a (continued)

α-FBL

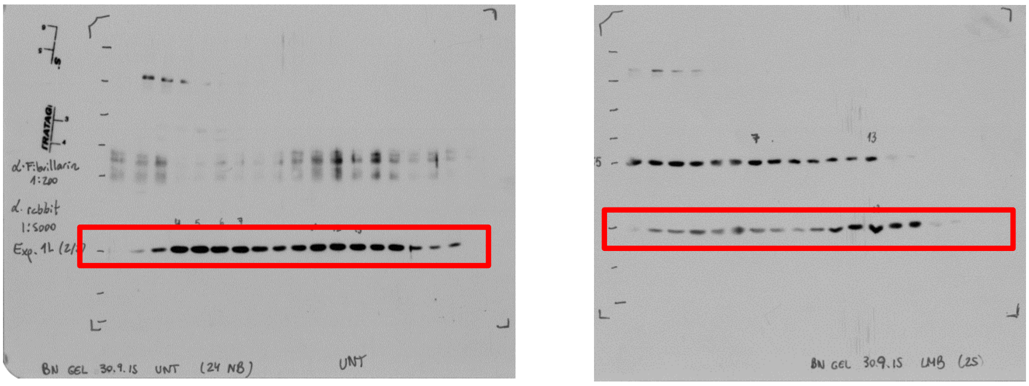

α-ENP1

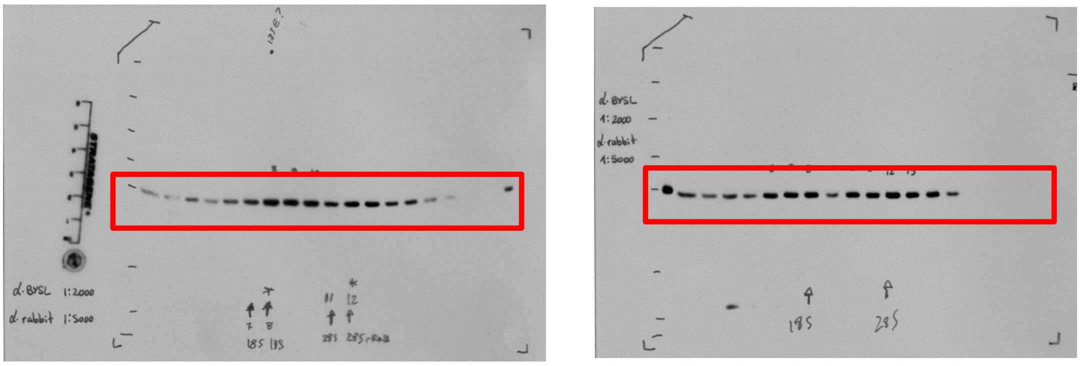

α-RRP12

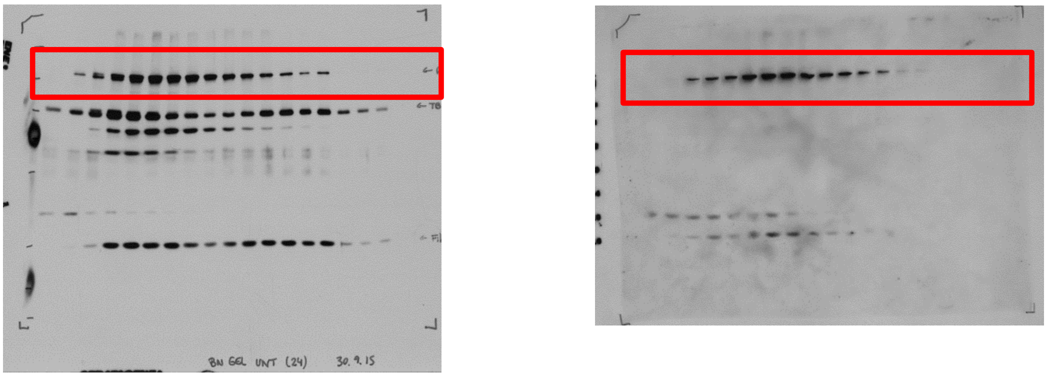

FIGURE 2b

5’-ITS1

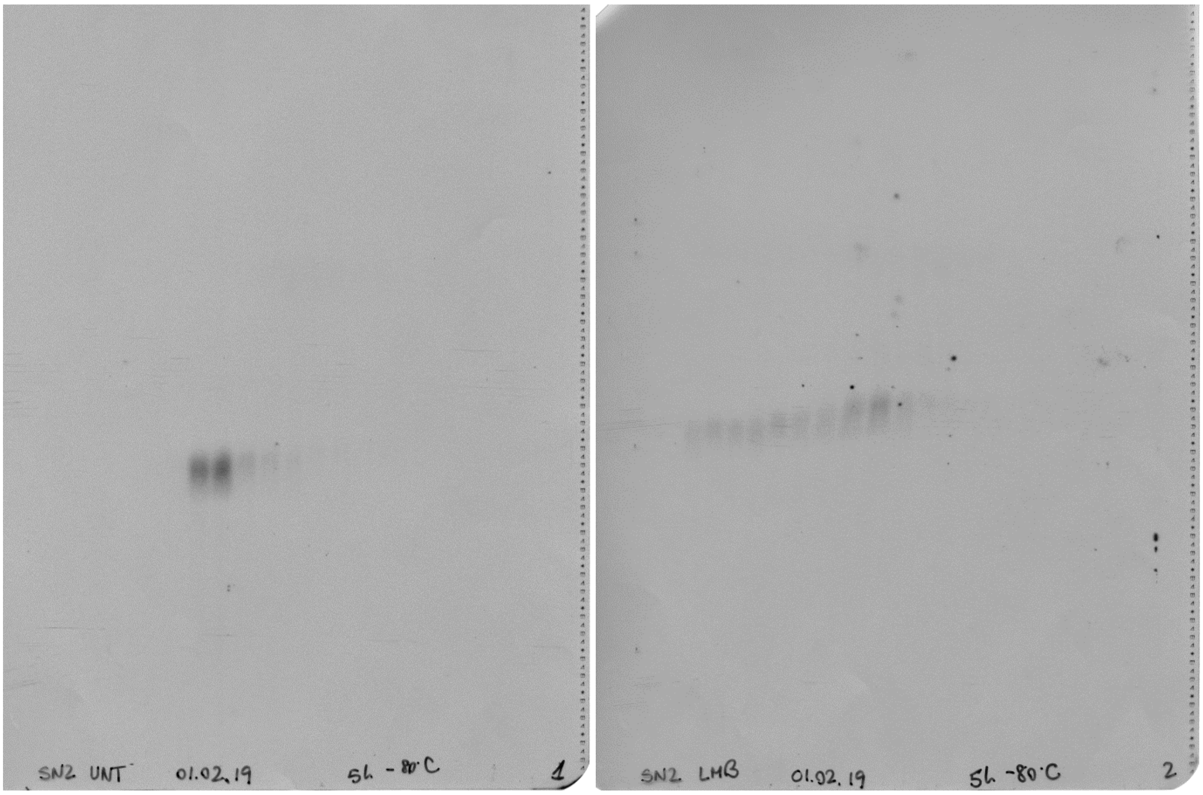

α-ENP1

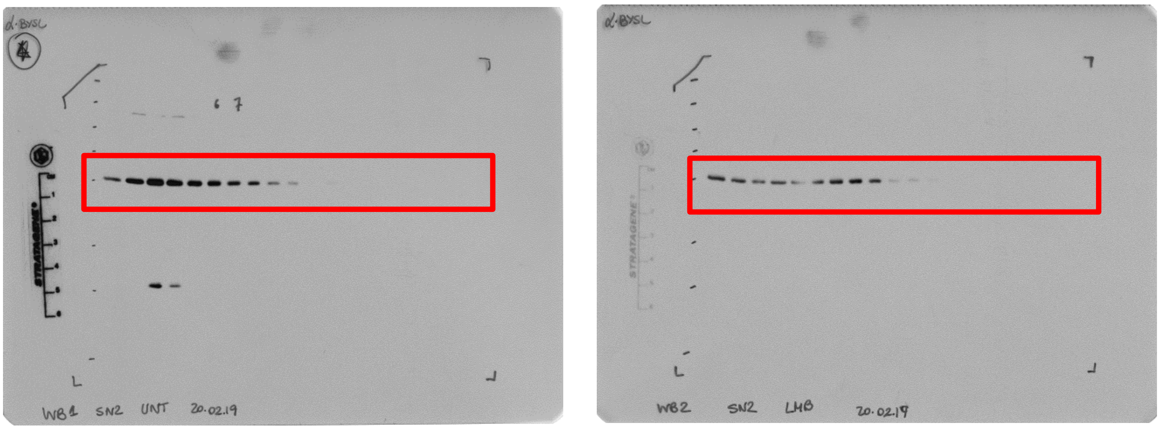

α-RRP12

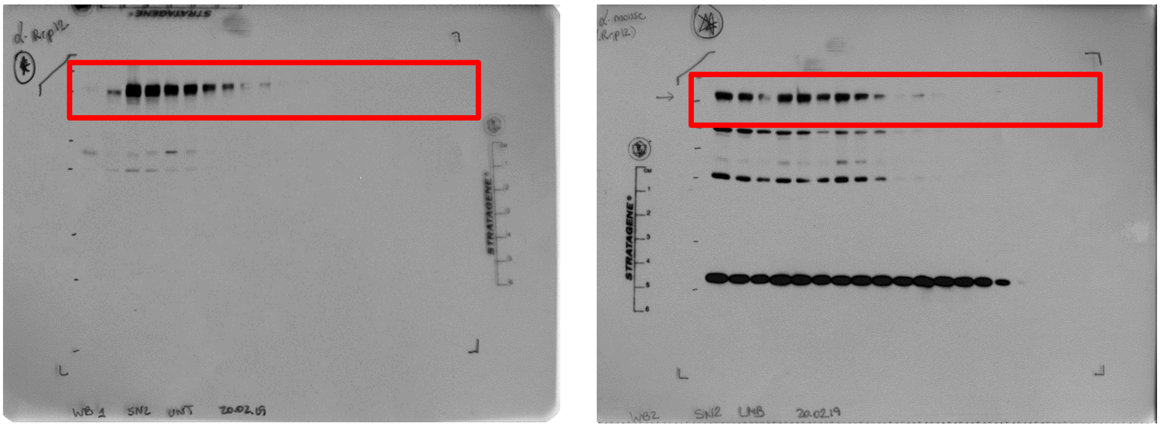

FIGURE 2c

α-ENP1

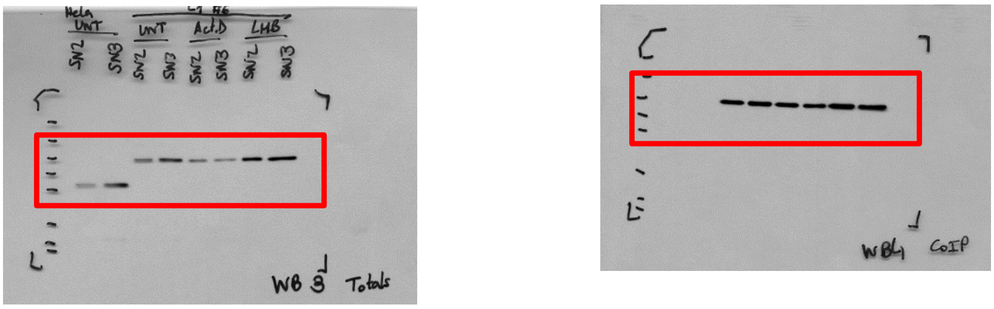

5’-ITS1

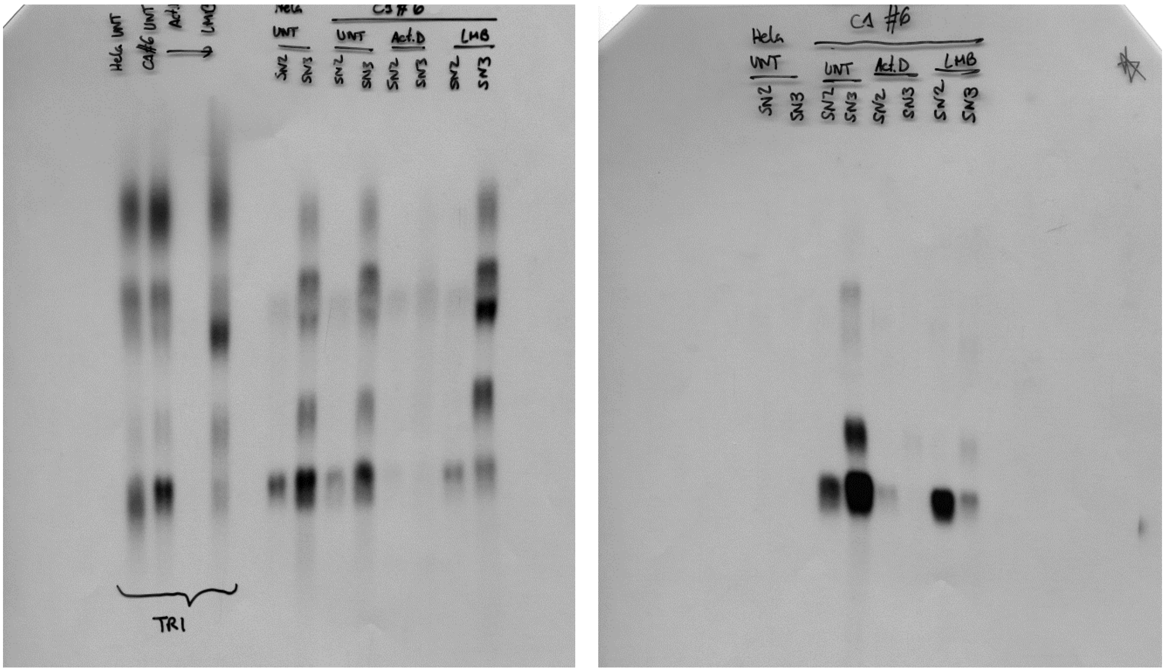

FIGURE 2d

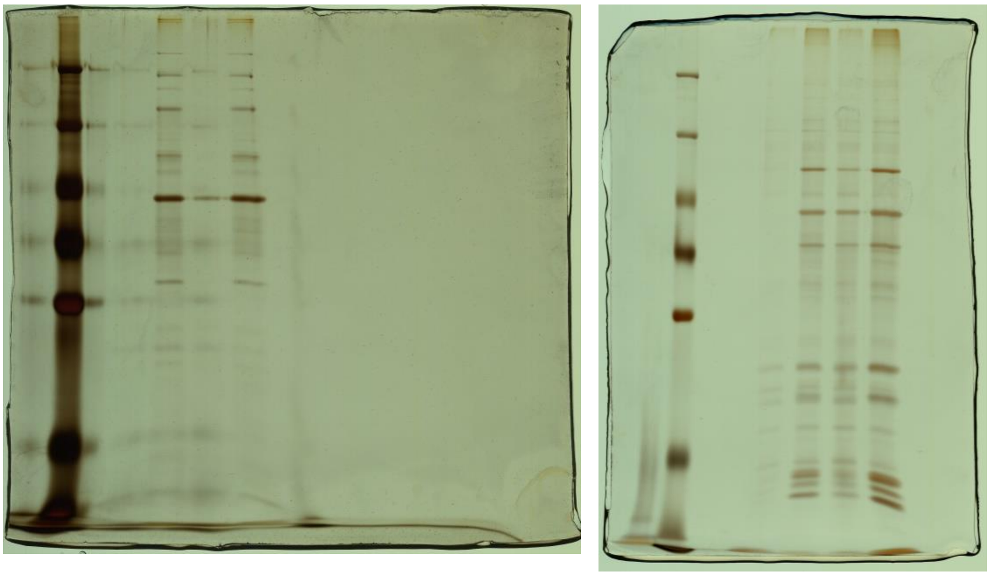

FIGURE 2e

α-ENP1

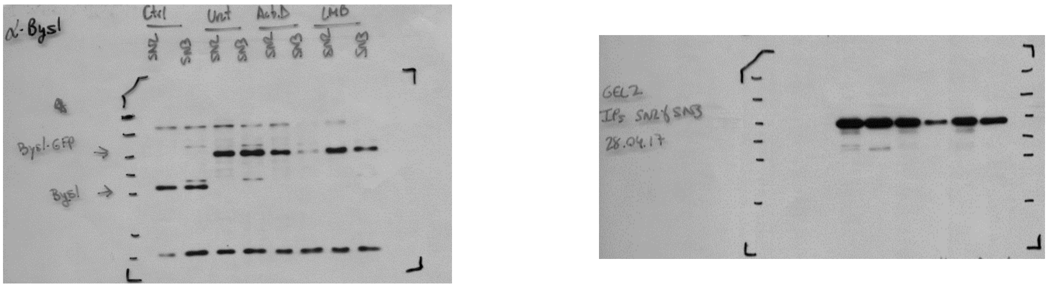

α-NOC4L

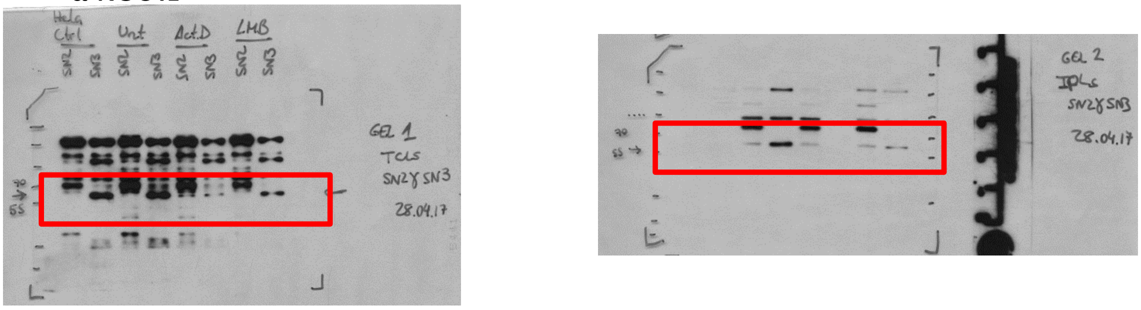

α-RRP12

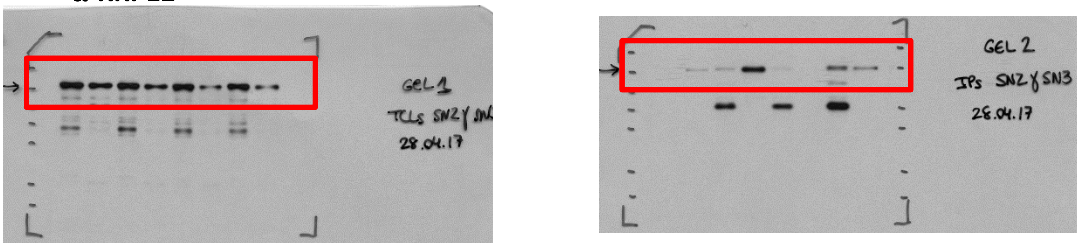

α-TSR1

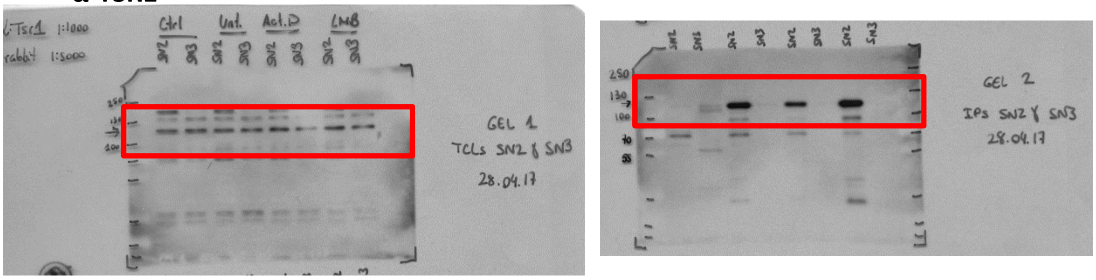

α-LTV1

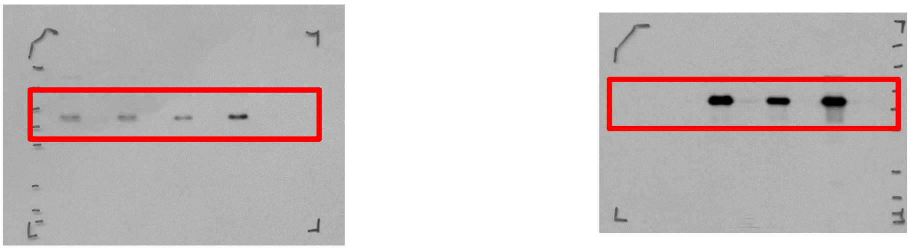

α-RIO2

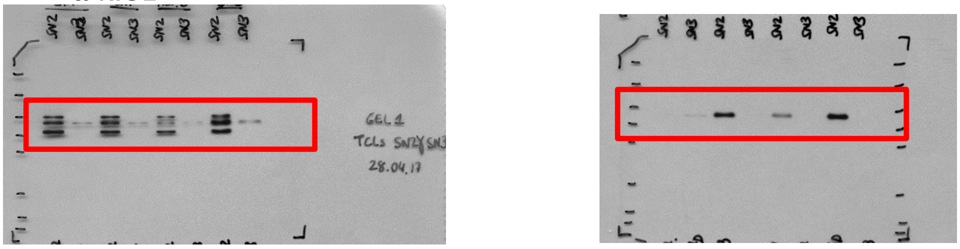

FIGURE 2f

α-ENP1

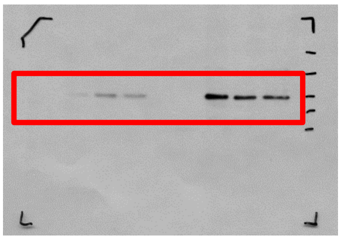

α-RRP12

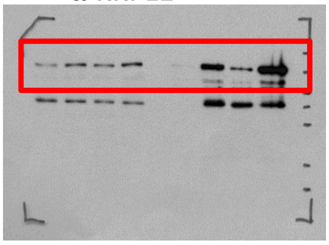

α-TSR1

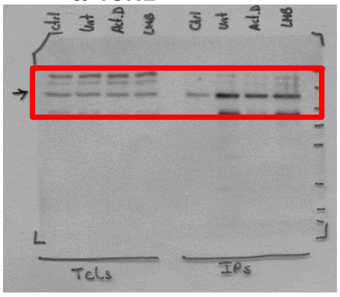

α-LTV1

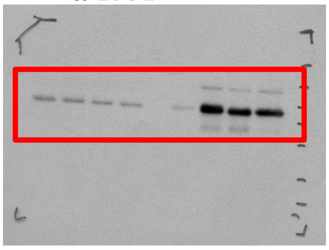

α-RIO2

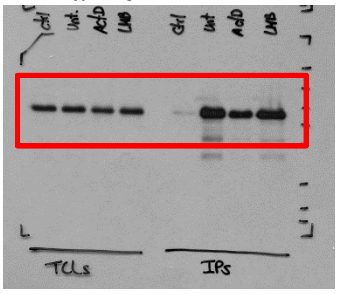

FIGURE 4a

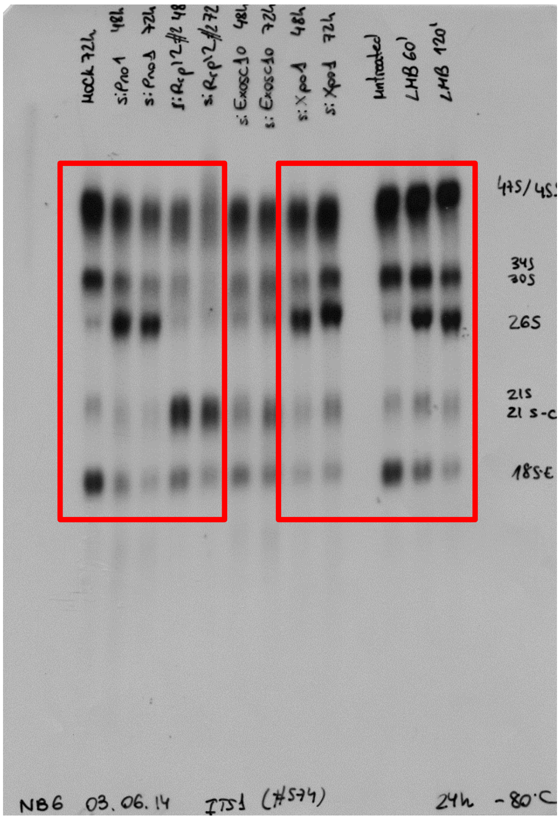

FIGURE 4b

α-TBL3

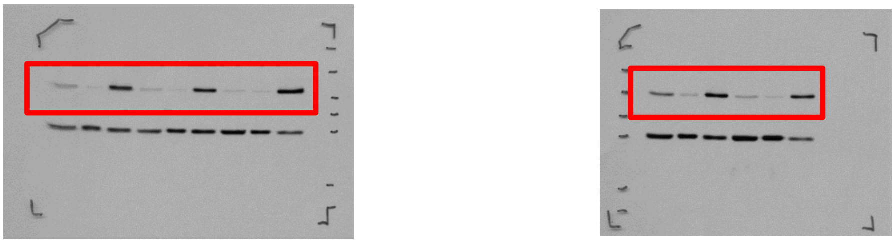

α-NOC4L

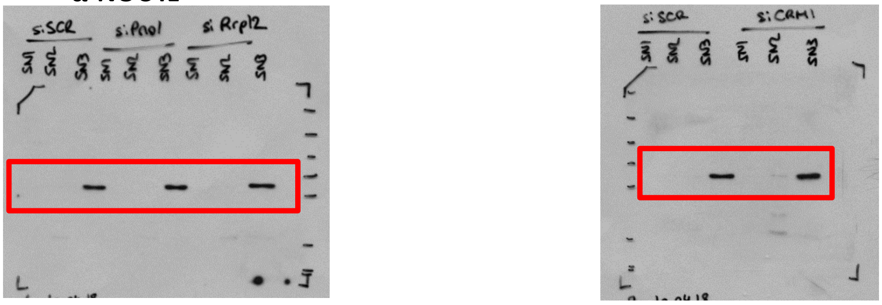

α-ENP1

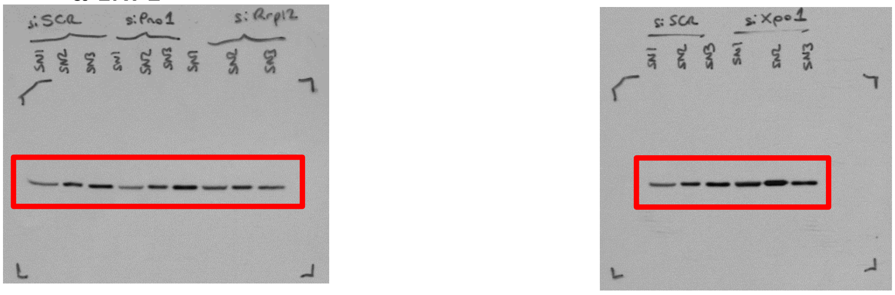

α-RRP12

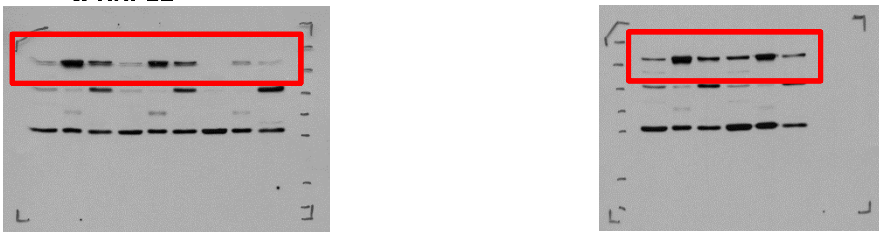

α-LTV1

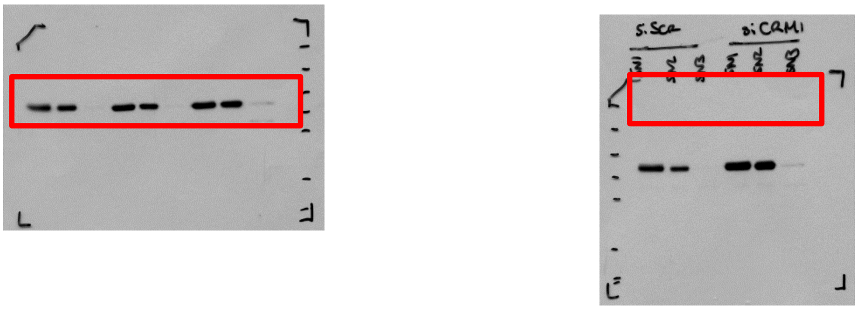

FIGURE 4c

α-TBL3

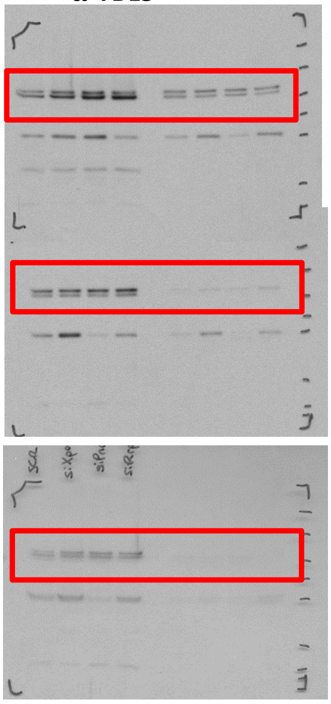

α-ENP1

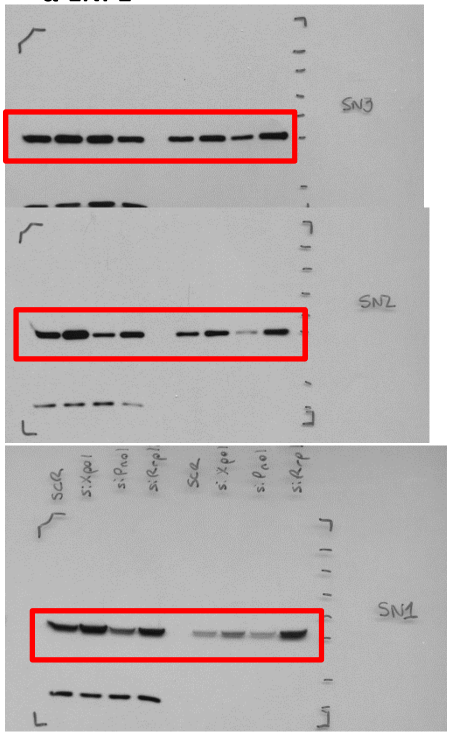

FIGURE 4d

α-ENP1

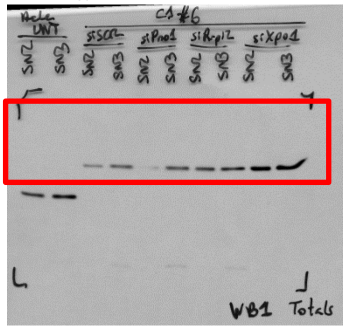

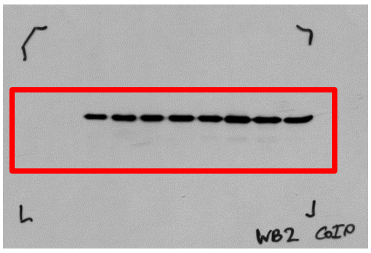

5'-ITS1

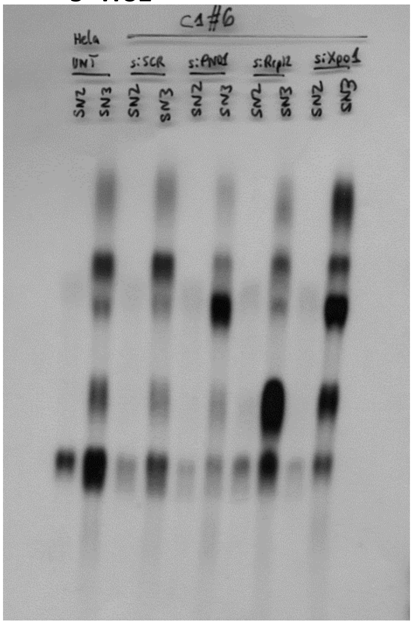

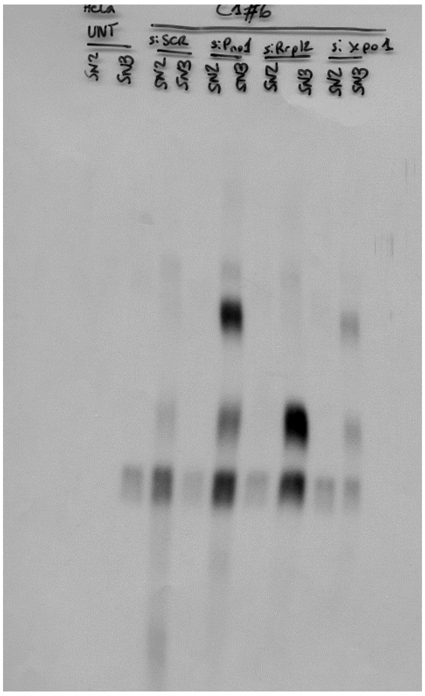

FIGURE 4e

α-ENP1

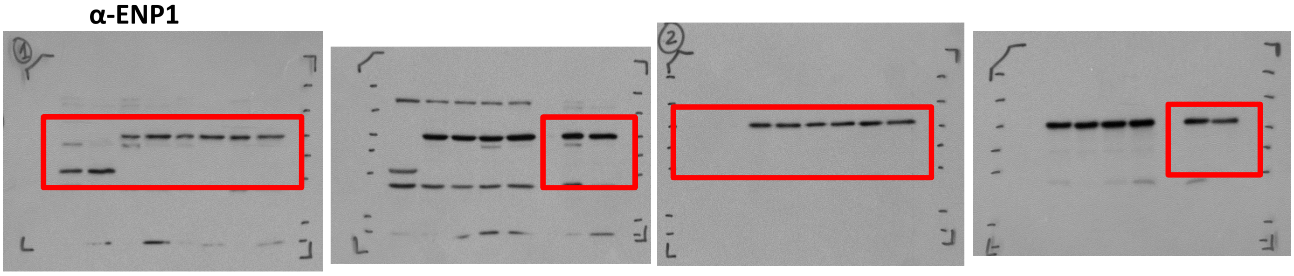

α-NOC4L

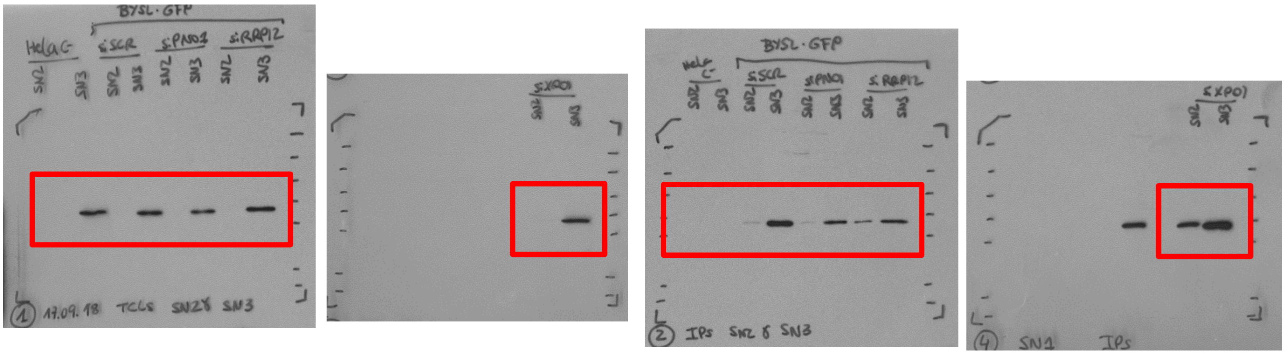

α-RRP12

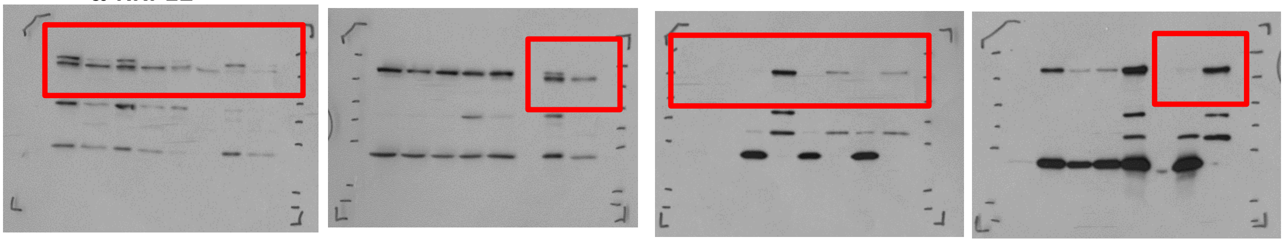

α-TSR1

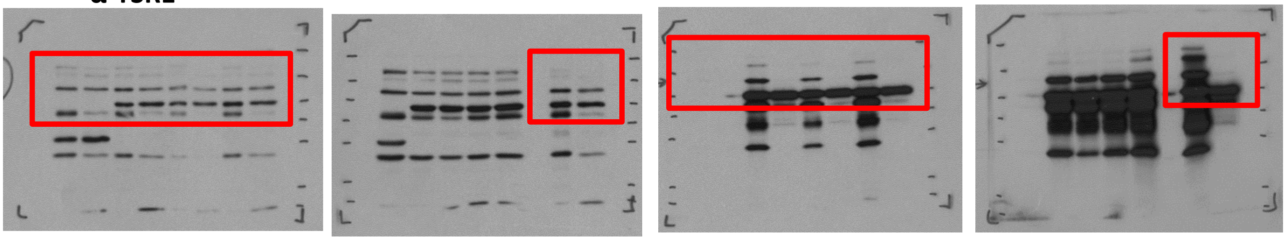

α-LTV1

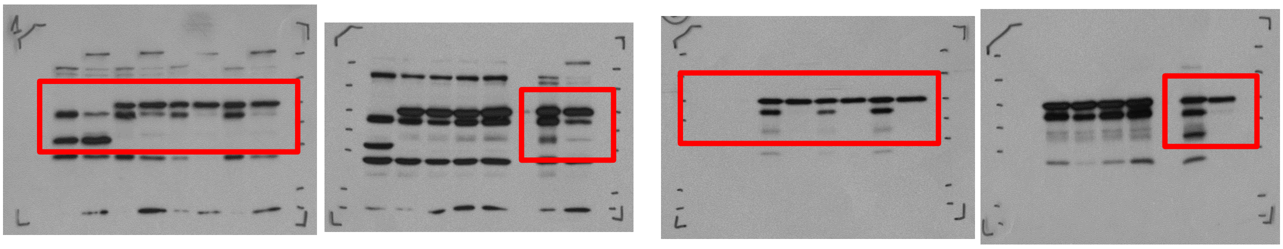

α-NOB1

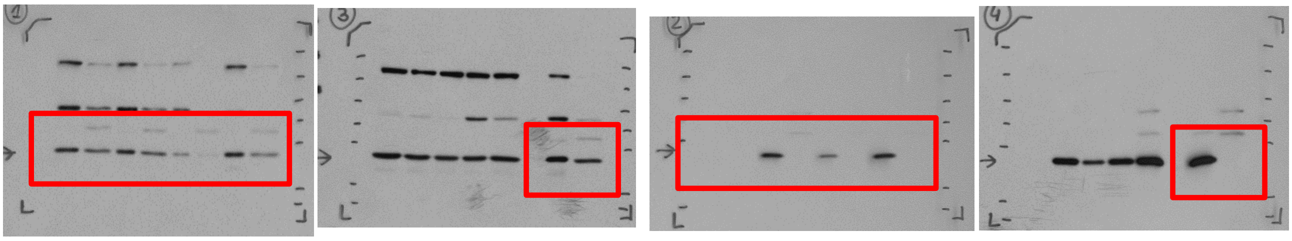

FIGURE 4e (continued)

α-ENP1

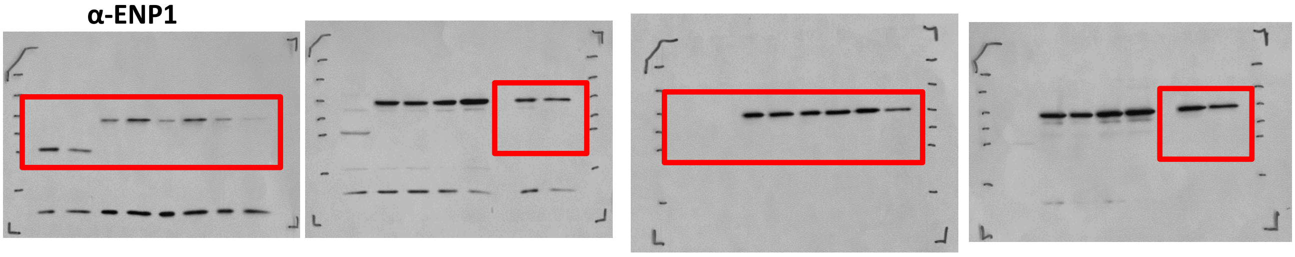

α-RIO2

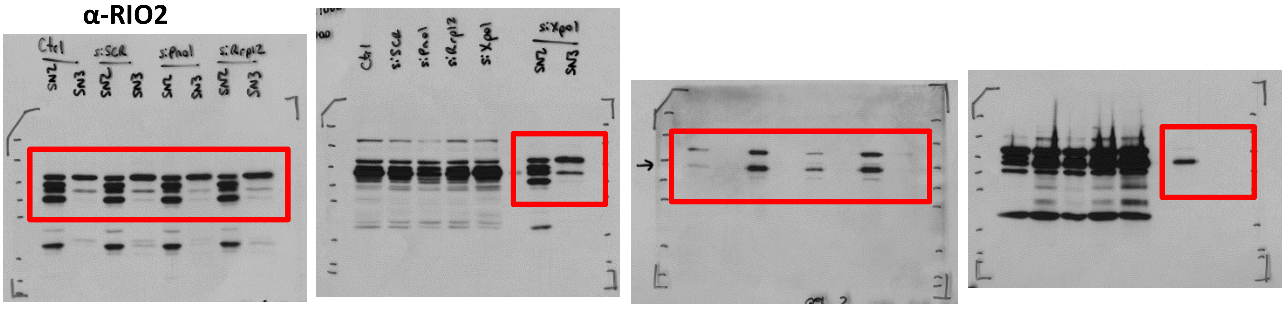

FIGURE 4f

α-ENP1

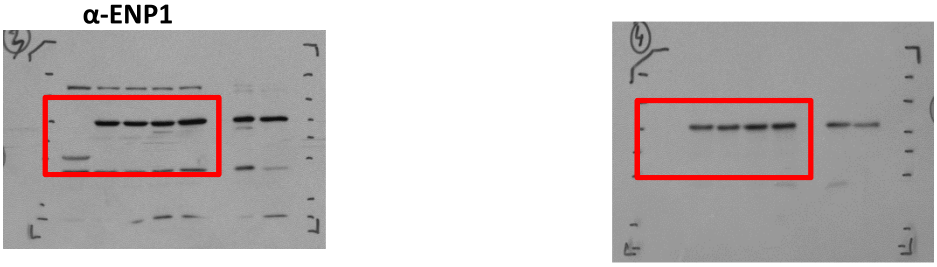

α-RRP12

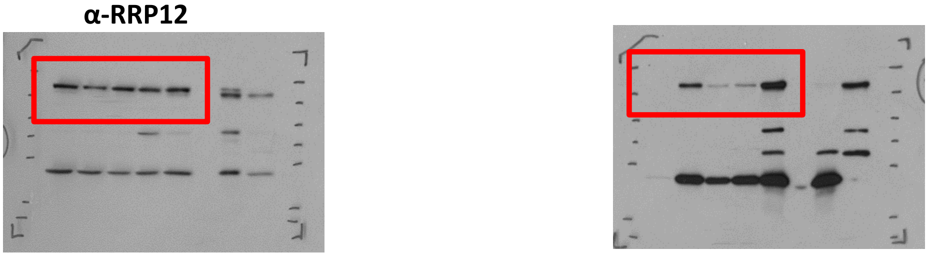

α-TSR1

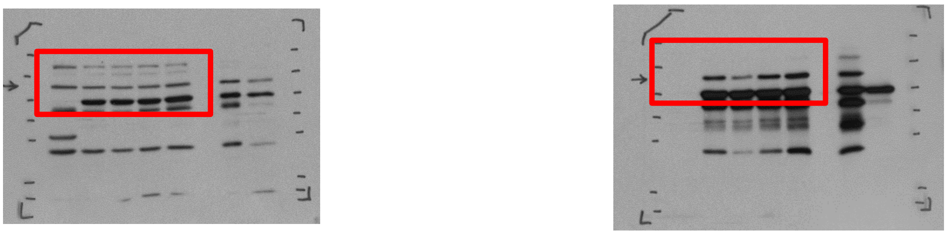

FIGURE 4f (continued)

α-LTV1

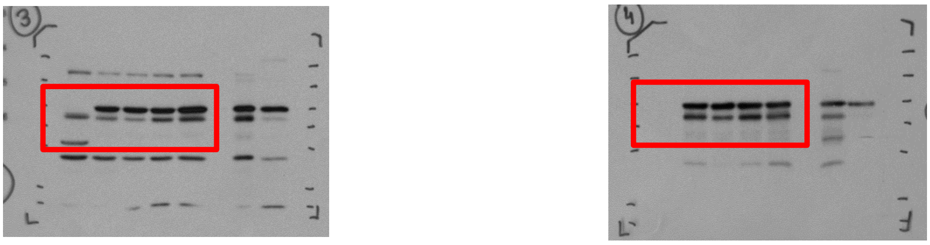

α-NOB1

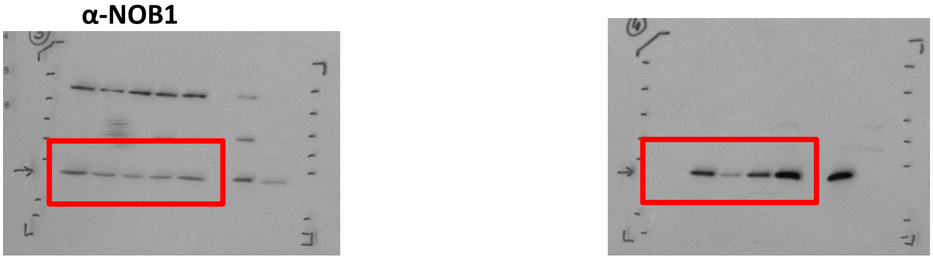

α-ENP1

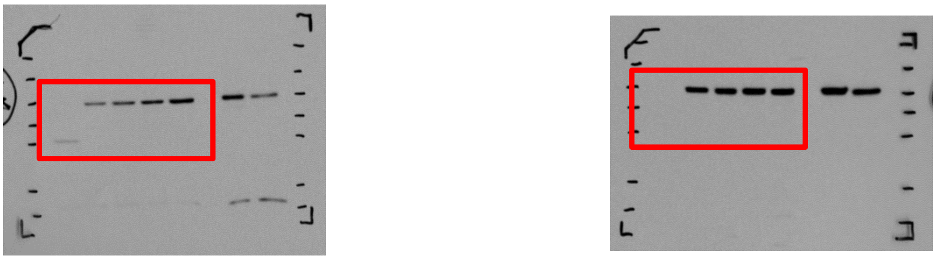

α-RIO2

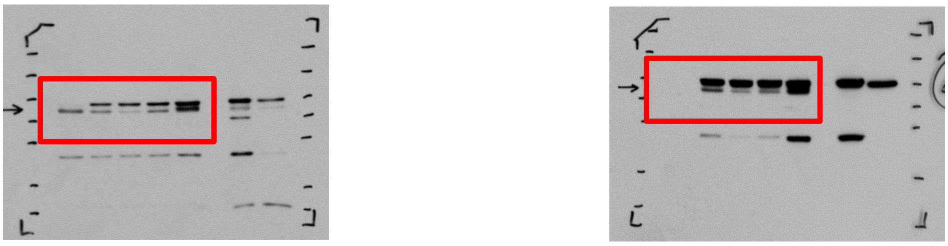

FIGURE 6a

α-RRP12

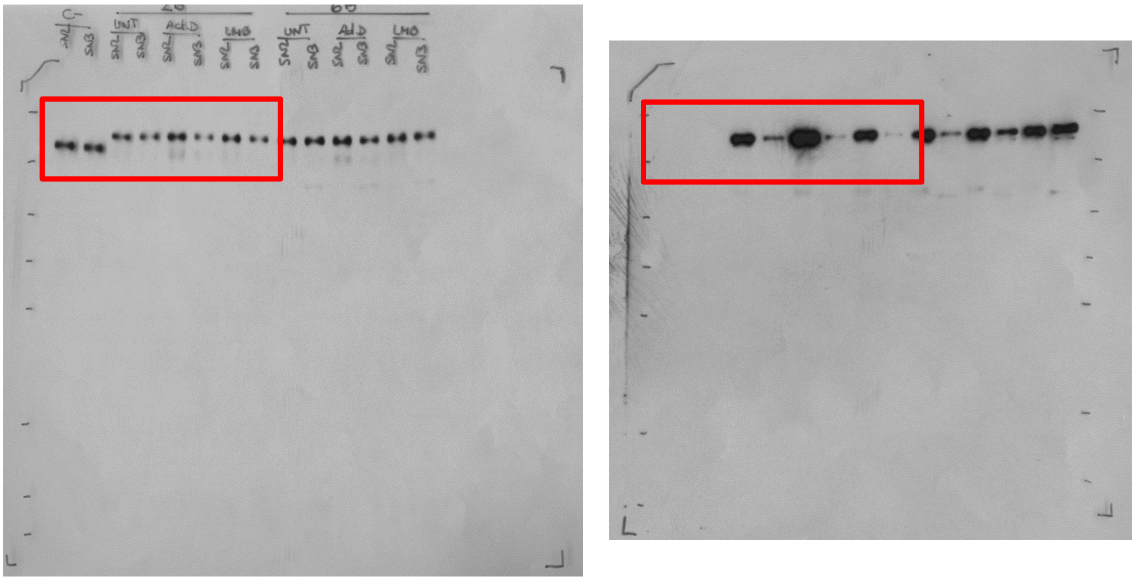

5'-ITS1

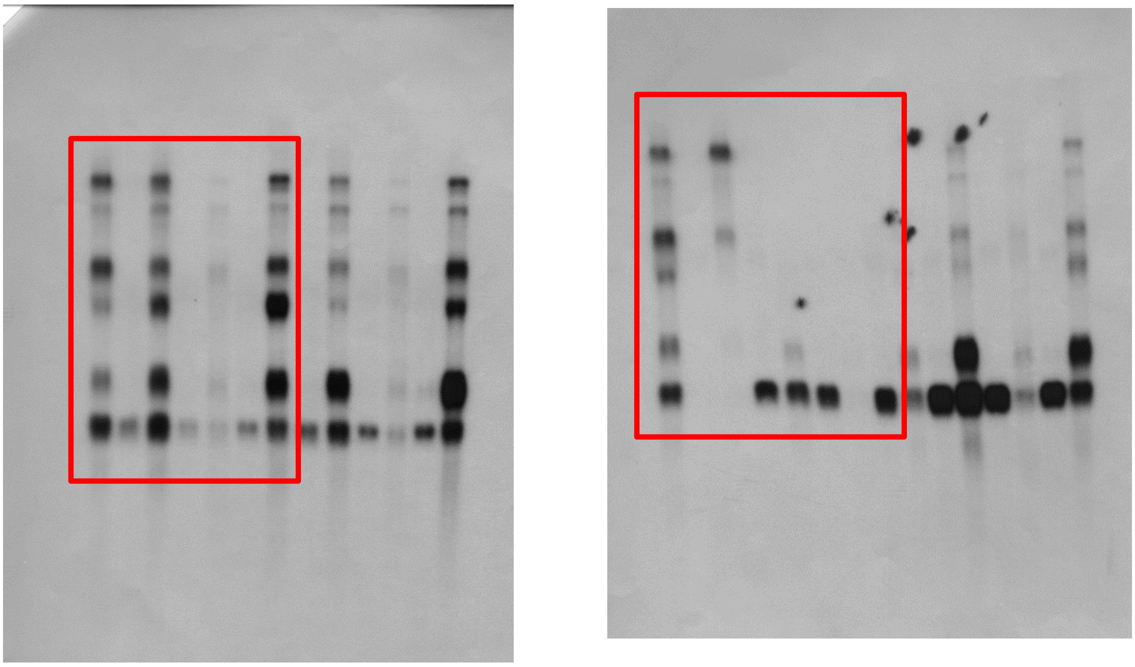

FIGURE 6b

α-RRP12

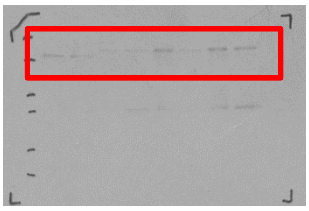
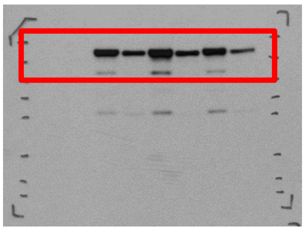

α-NOC4L

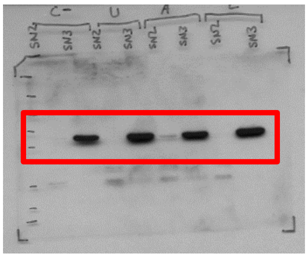
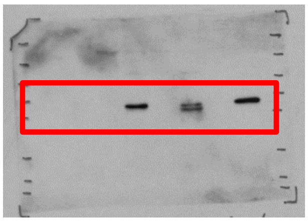

α-ENP1

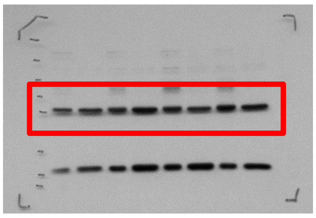
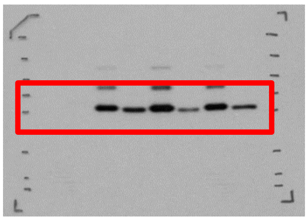

α-TSR1

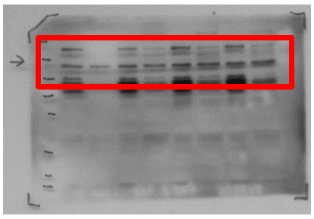
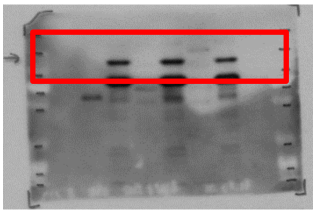

α-LTV1

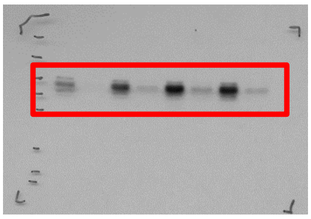
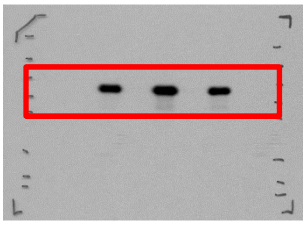

α-RIO2

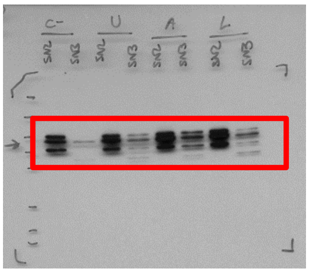
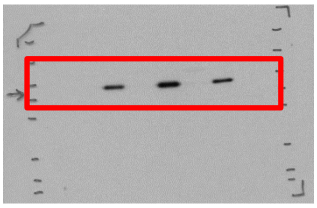

FIGURE 6c

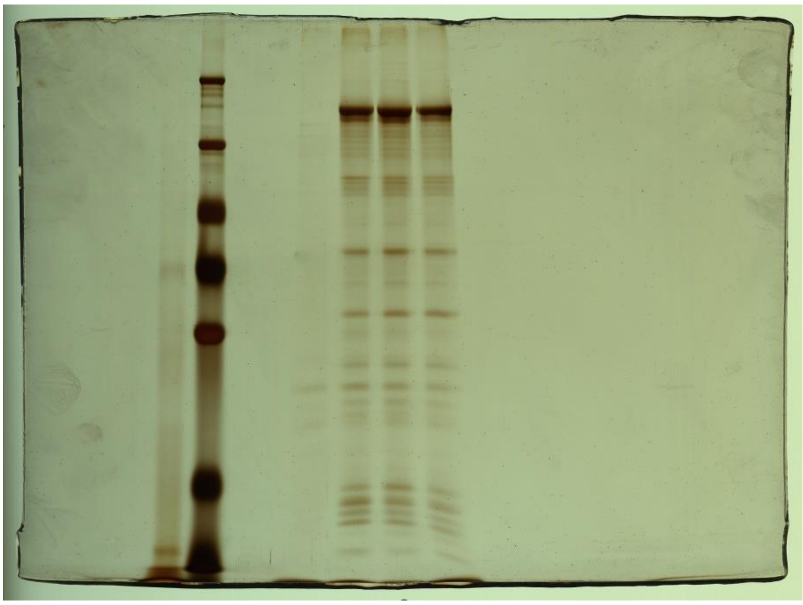

FIGURE 6d

α-PARN

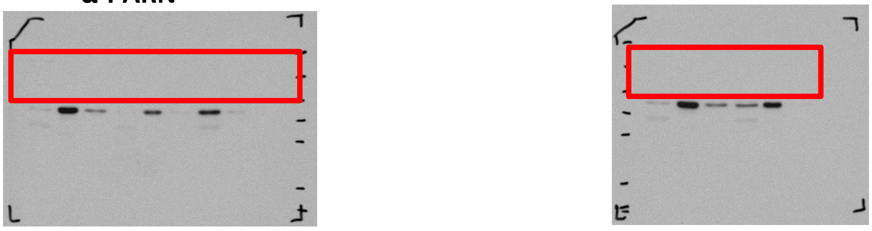

α-RRP12

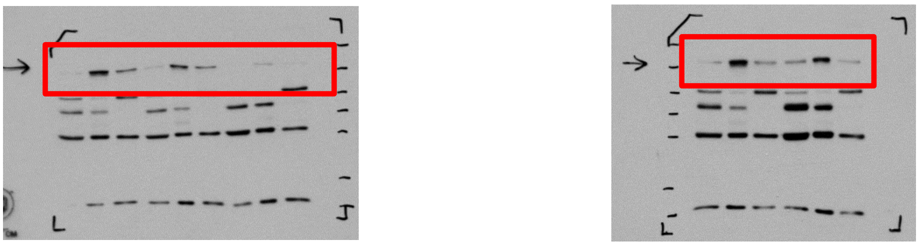

α-TBL3

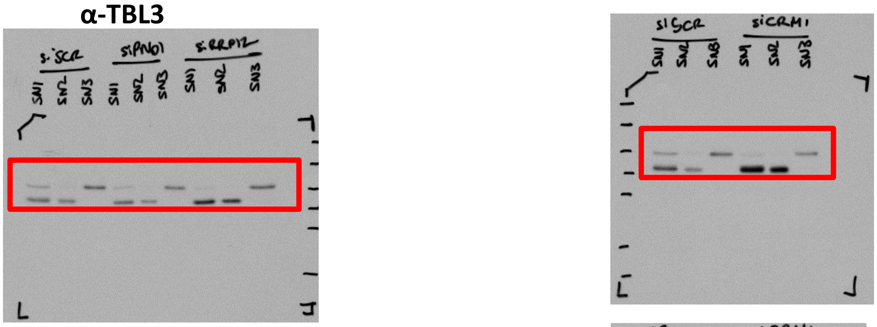

α-LTV1

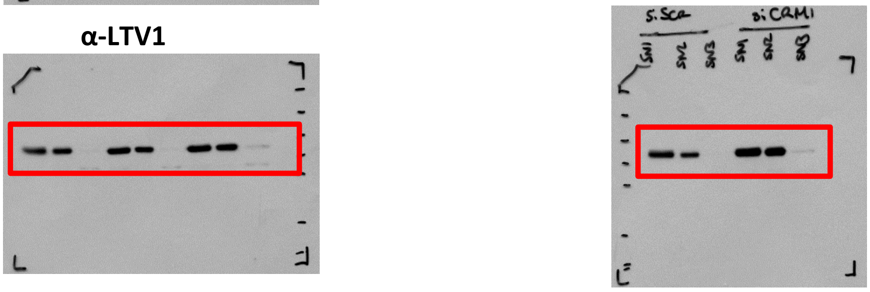

FIGURE 6e

α-PARN

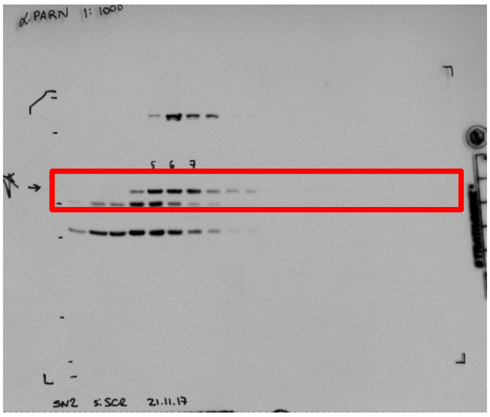

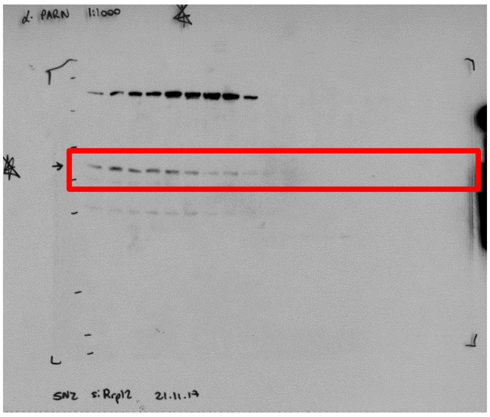

α-ENP1

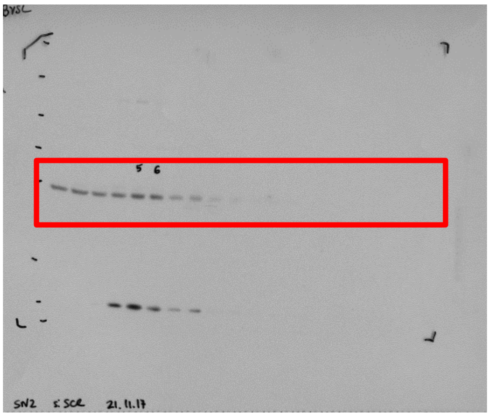

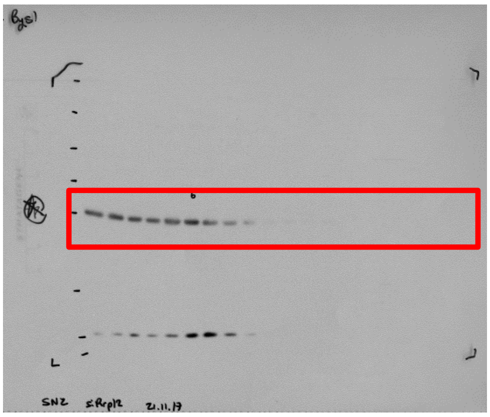

α-LTV1

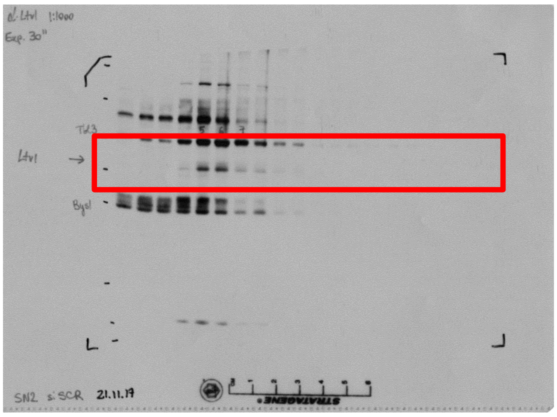

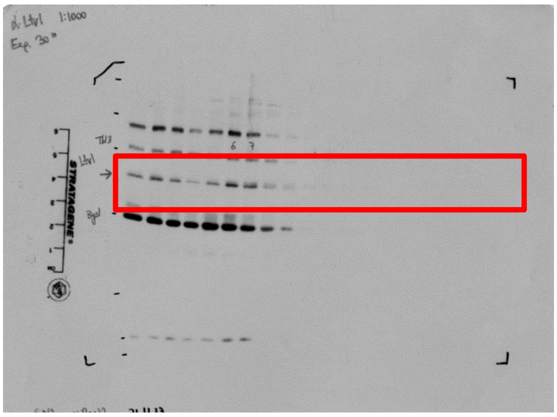

FIGURE 7a

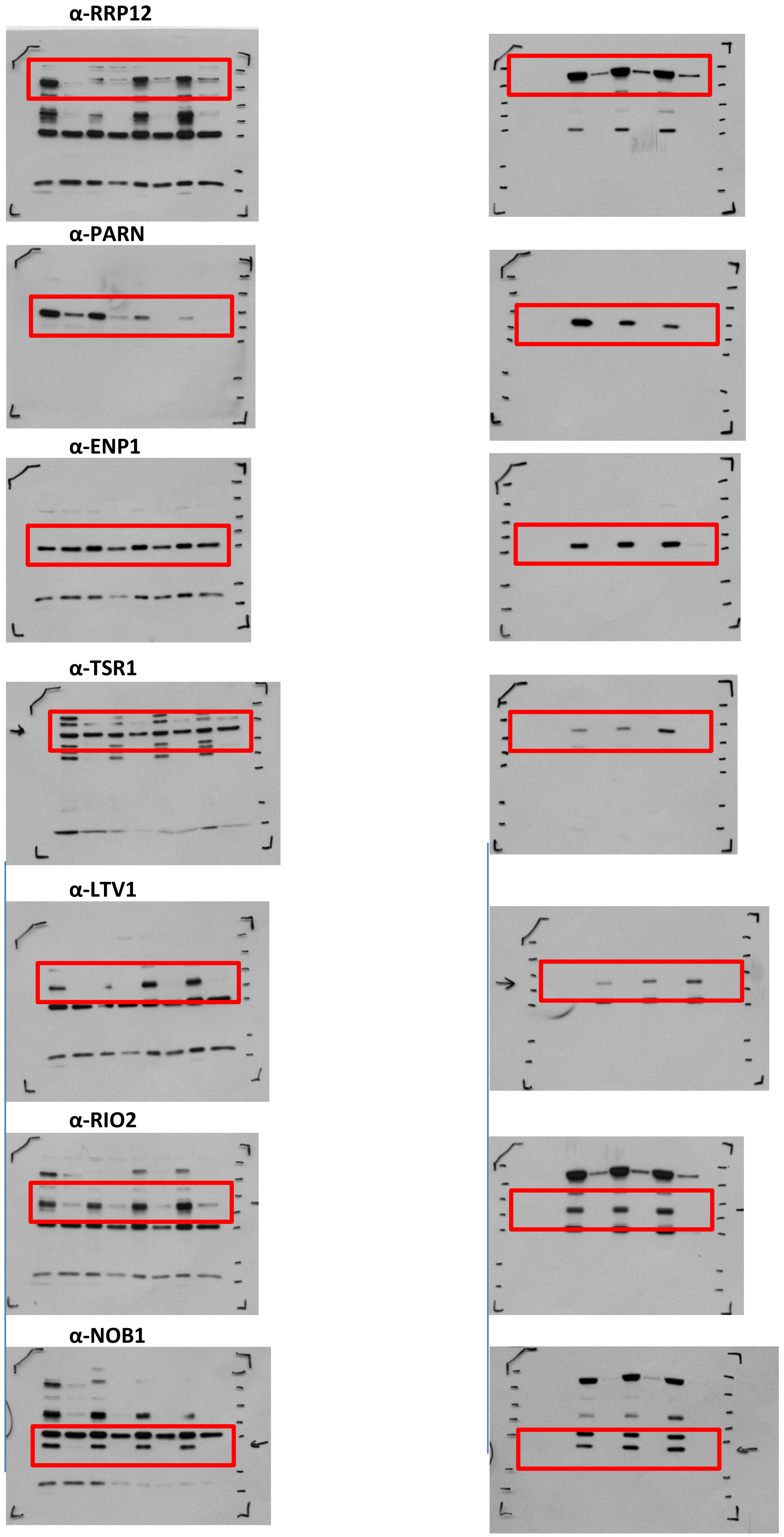

FIGURE 7b

α-RRP12

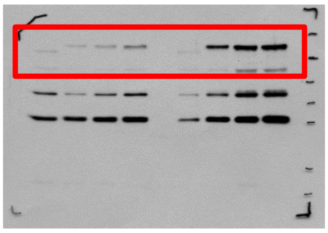

α-PARN

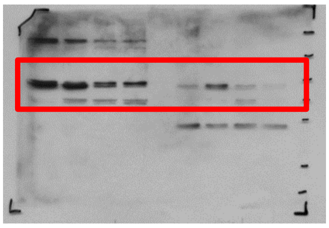

α-ENP1

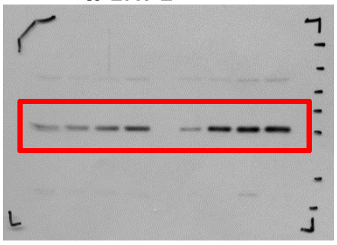

α-LTV1

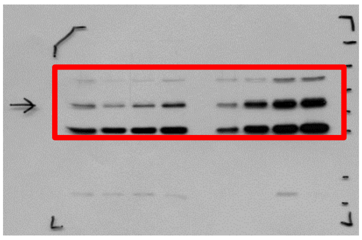

α-RIO2

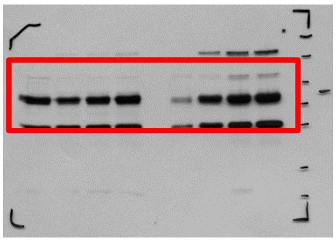

α-NOB1

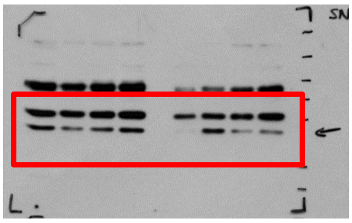

FIGURE S2c

α-TBL3 & α-ENP1

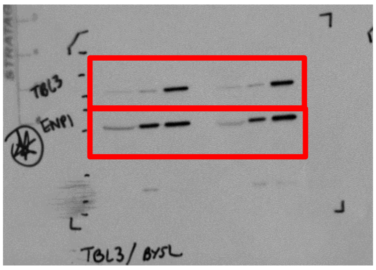

α-RRP12

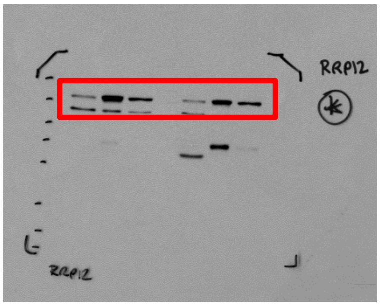

α-LTV1

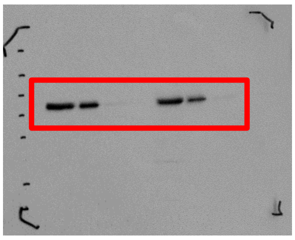

α-PES1

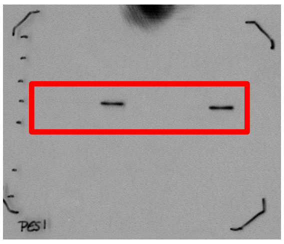

FIGURE S4a

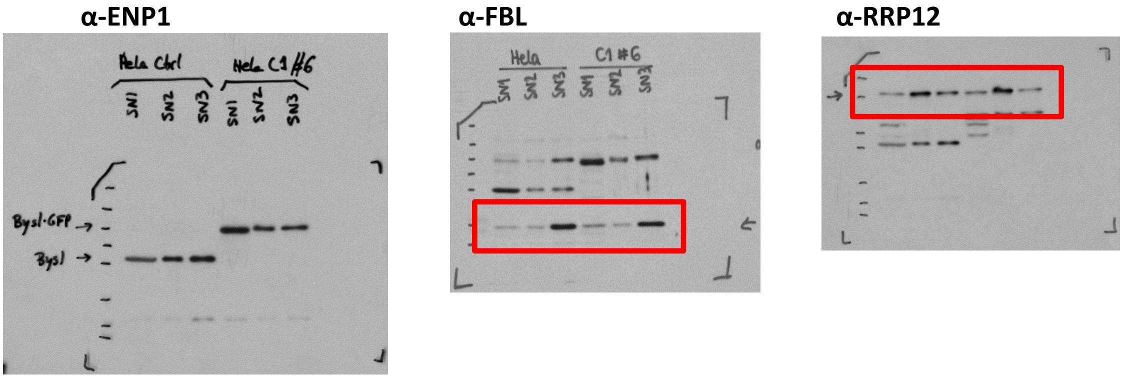

FIGURE S4b

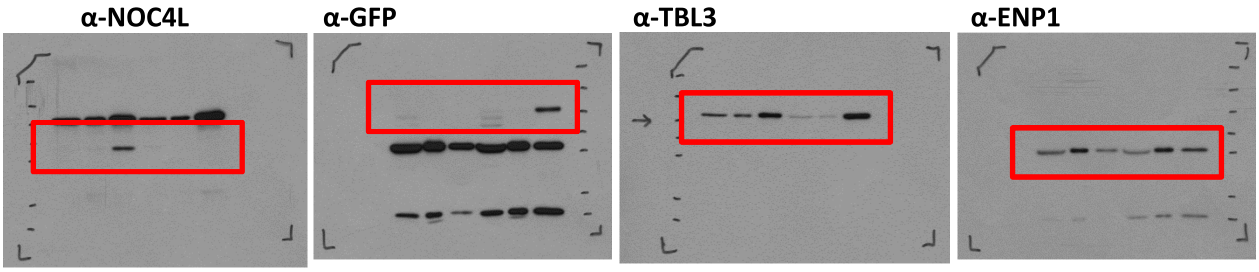

FIGURE S4c

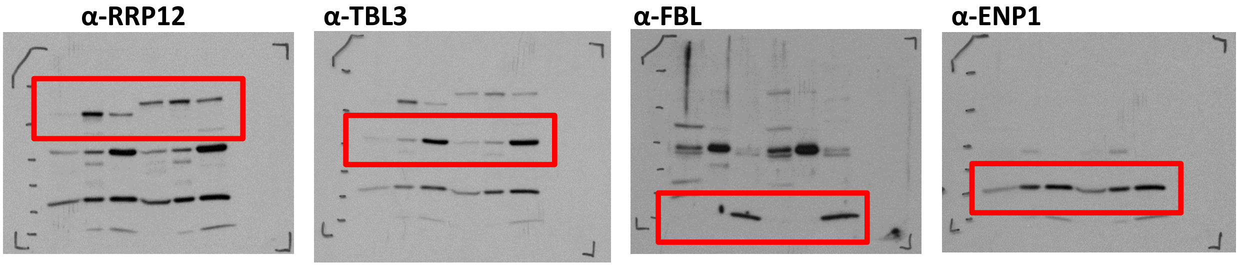

FIGURE S4d

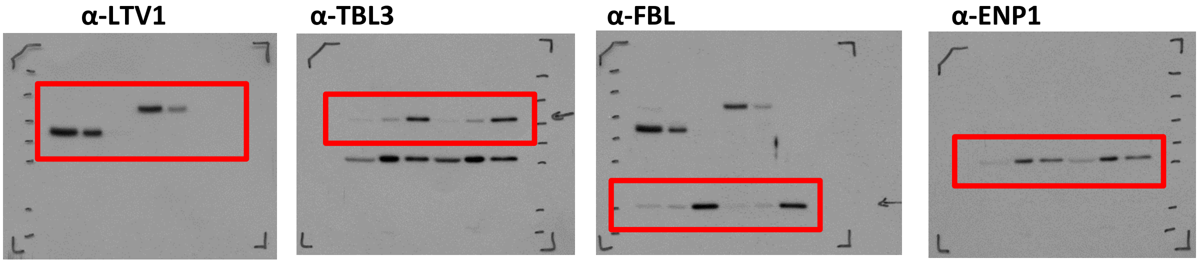

FIGURE S4i

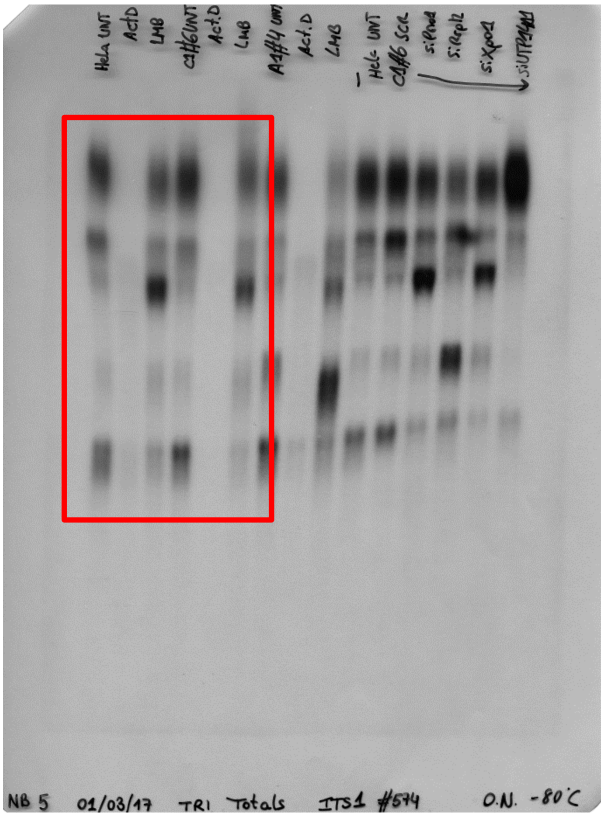

FIGURE S4j

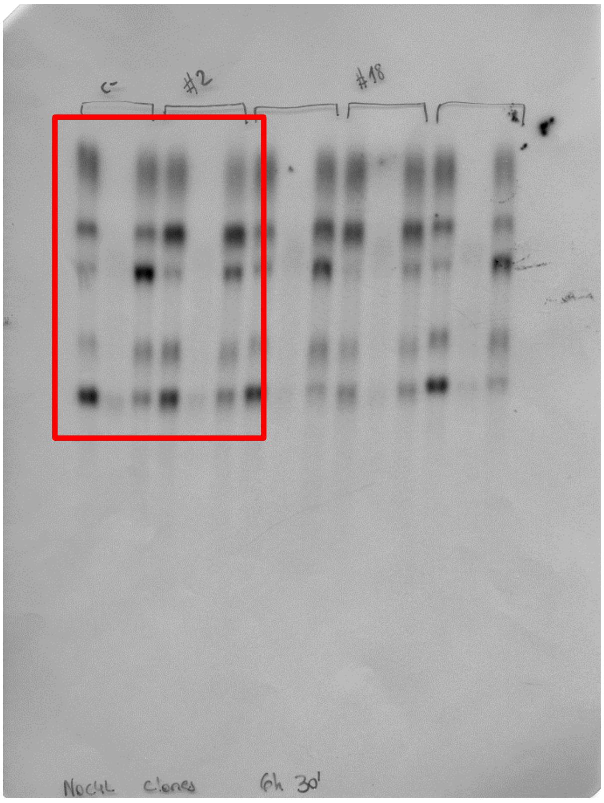

FIGURE S4k

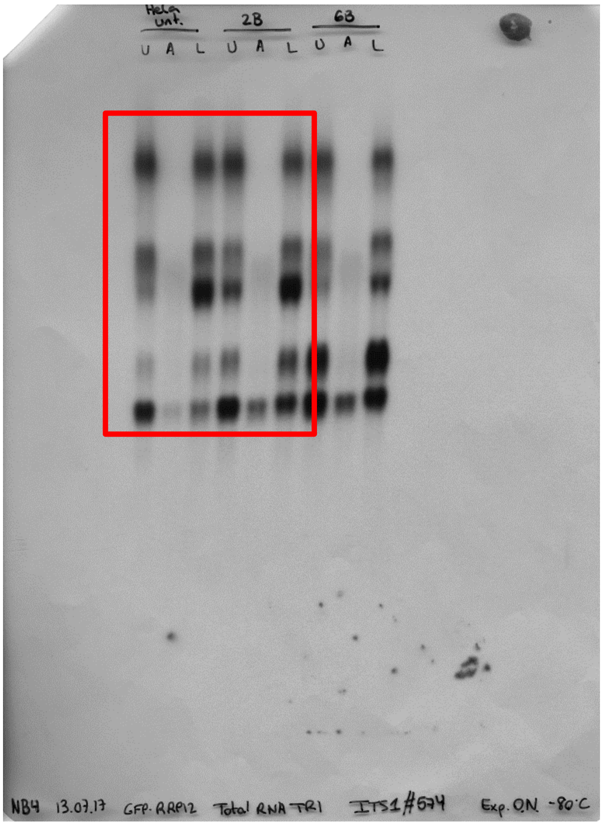

FIGURE S4l

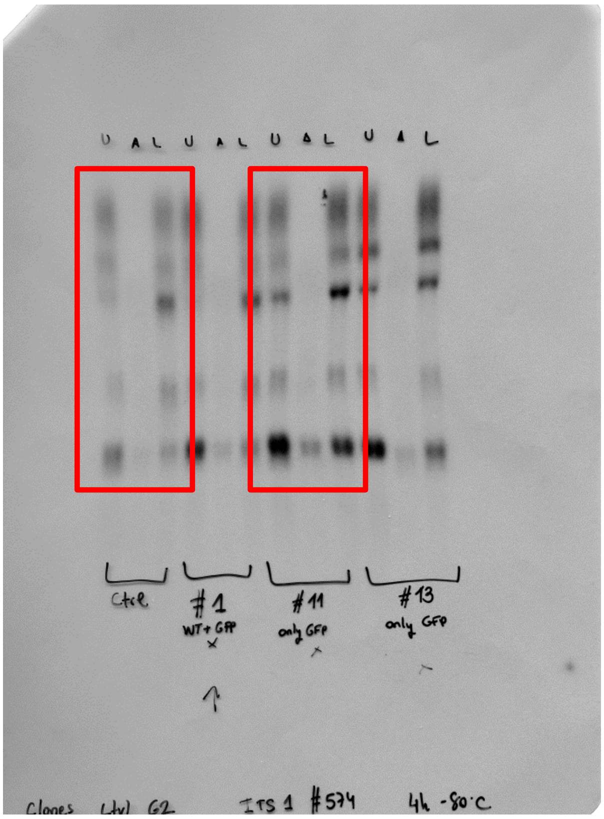

FIGURE S5

NB SN3 5'-ITS1

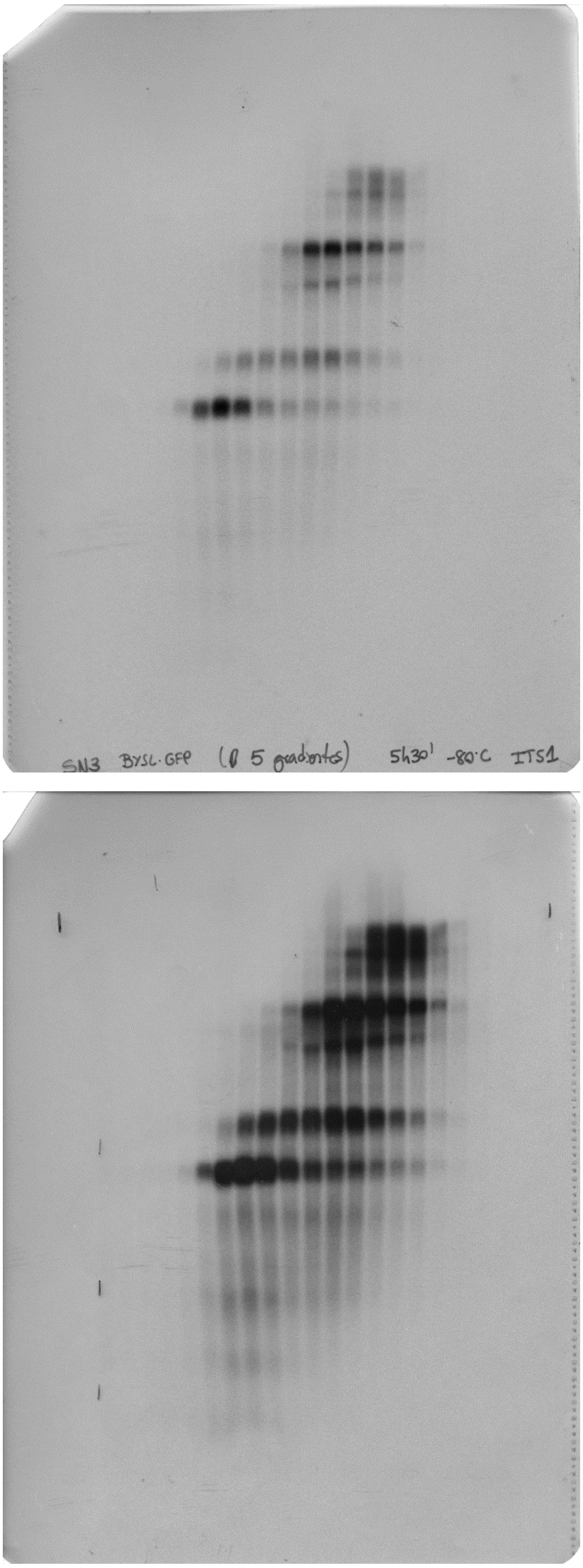

NB SN2 5'-ITS1

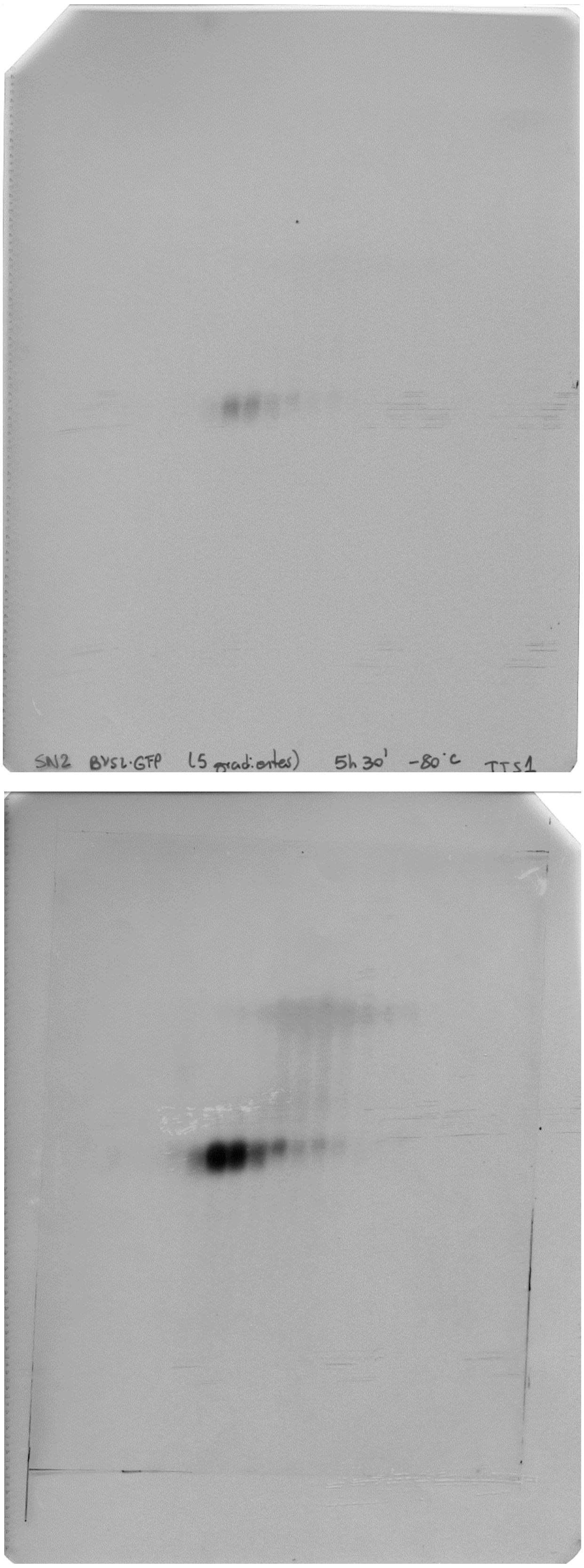

FIGURE S5 (continued)

WB SN3 α-ENP1

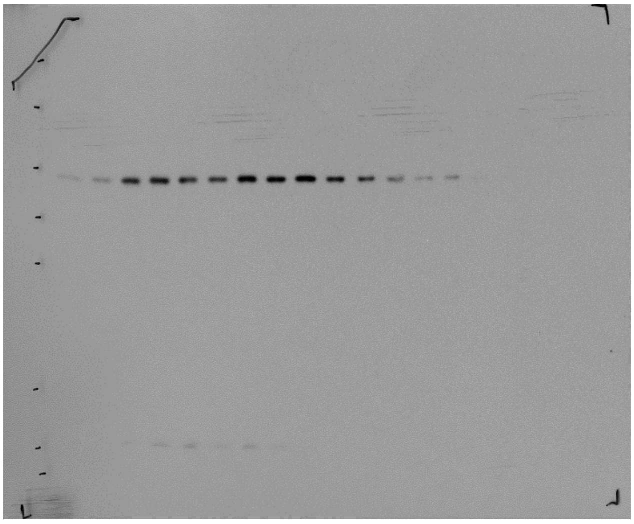

WB SN2 α-ENP1

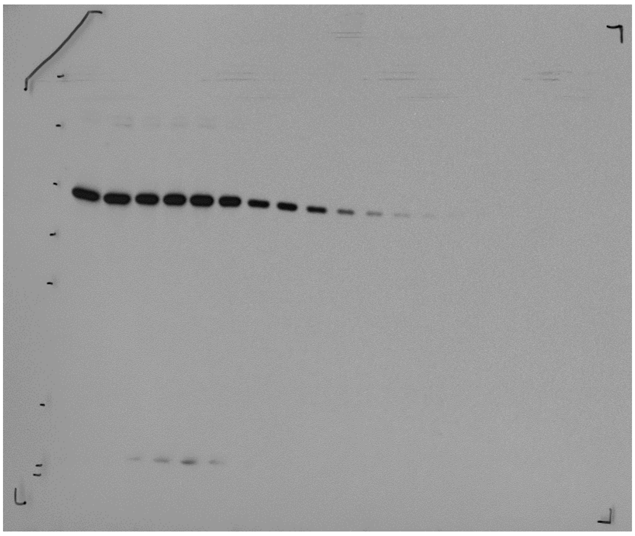

WB SN3 α-RRP12

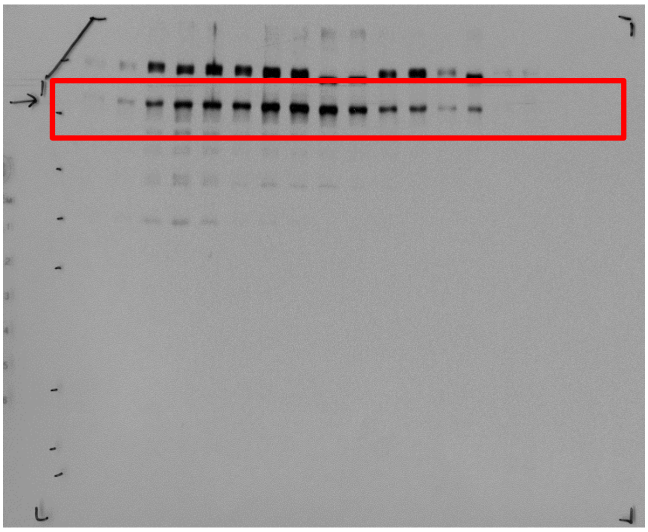

WB SN2 α-RRP12

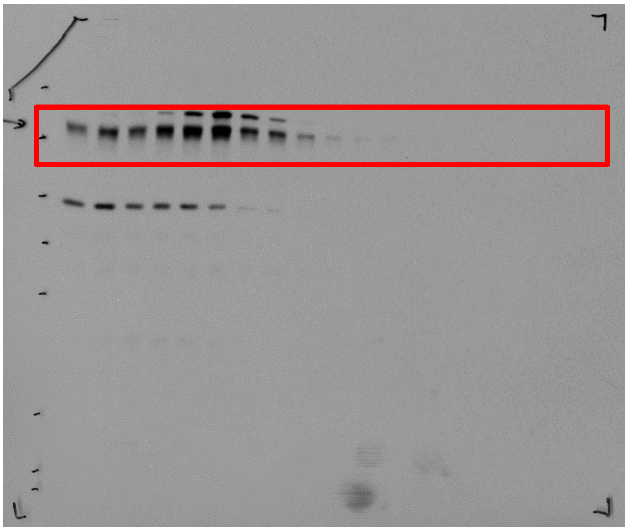

WB SN3 α-NOC4L

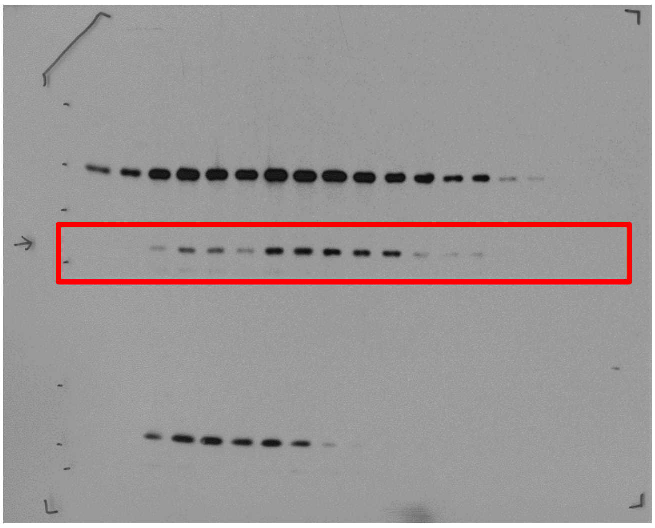

WB SN2 α-LTV1

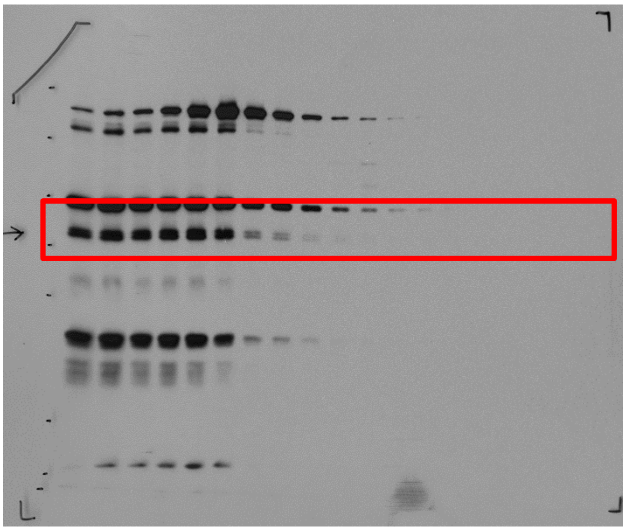

FIGURE S5 (continued)

WB COIP α-ENP1

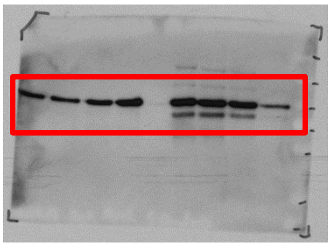

WB COIP α-RRP12

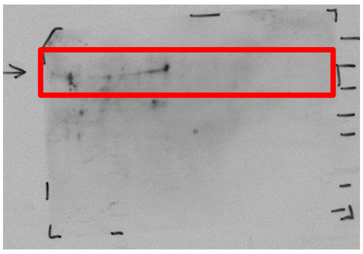

WB COIP α-NOC4L

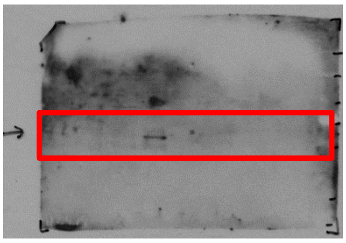

WB COIP α-HEATR1

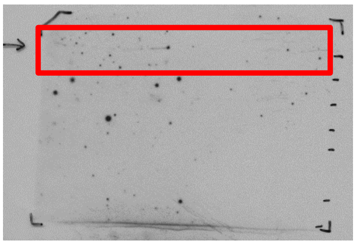

WB COIP α-LTV1

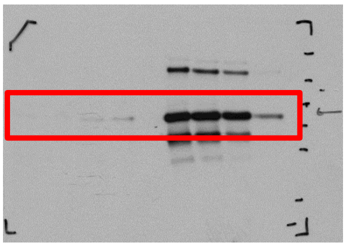

FIGURE S6

si-ctrl (SN3)

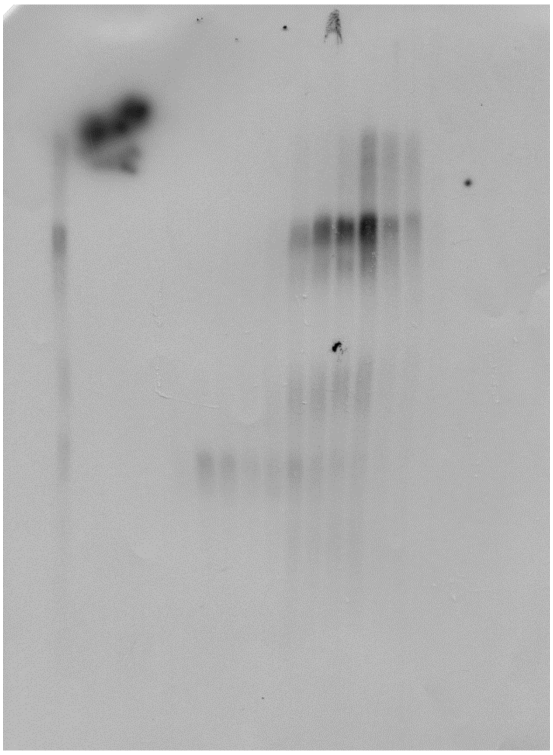

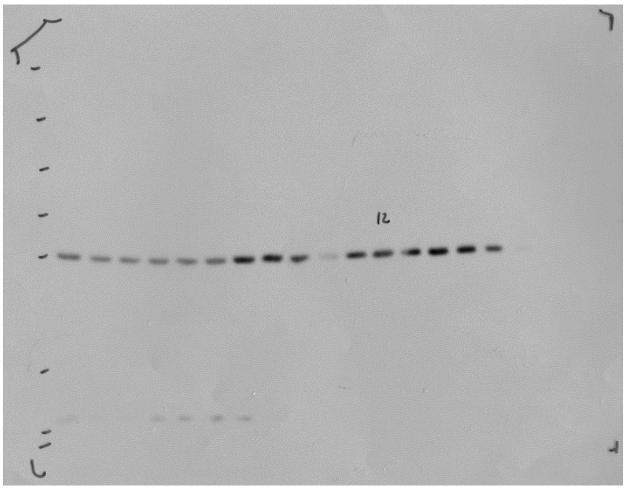

FIGURE S6 (continued)

si-PNO1 (SN3)

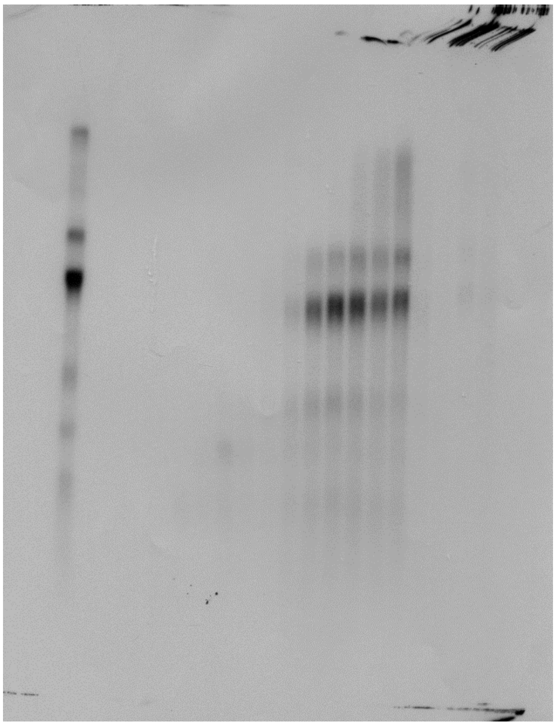

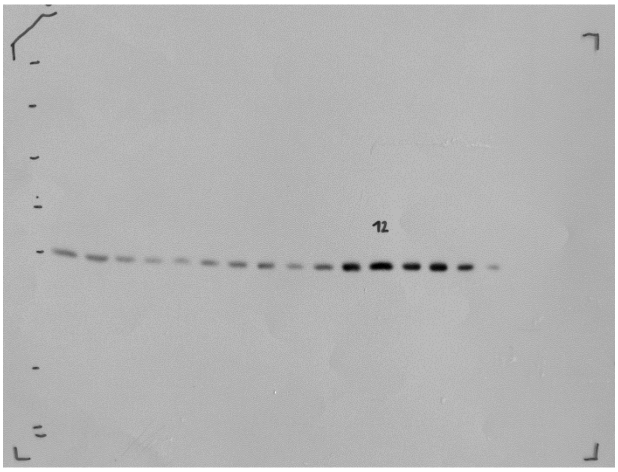

si-RRP12 (SN3)

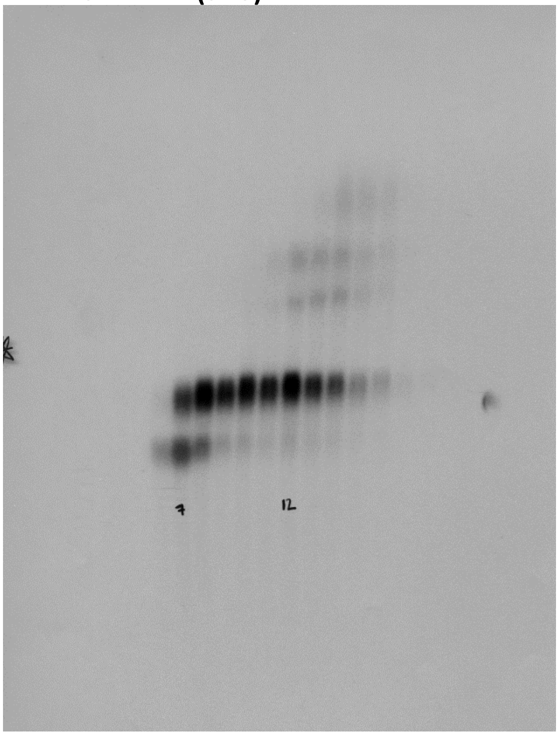

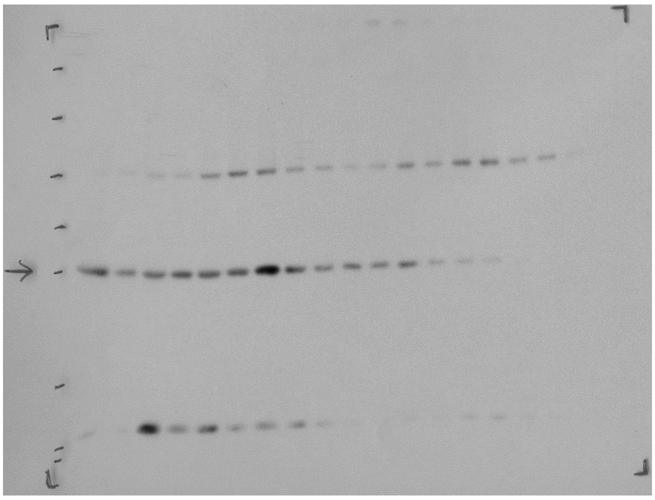

FIGURE S7a

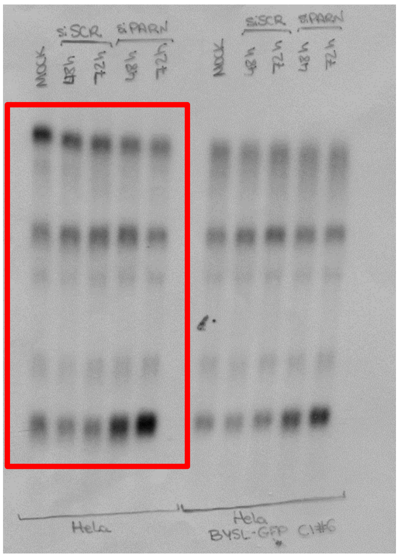

FIGURE S7b

α-PARN

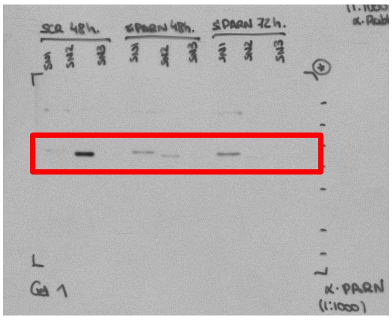

α-TBL3

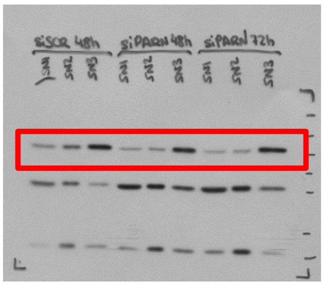

α-ENP1

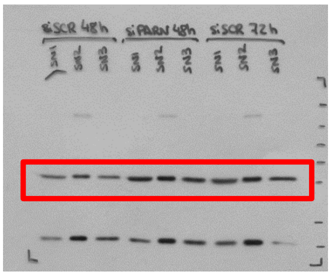

α-RRP12

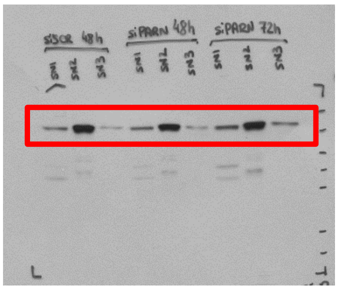

α-TSR1

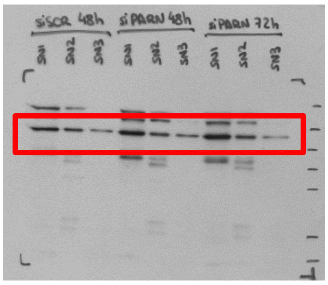

α-LTV1

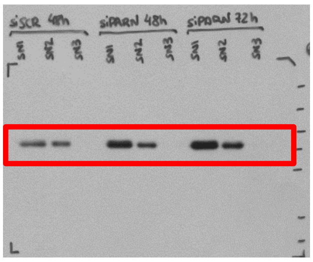

α-RIO2

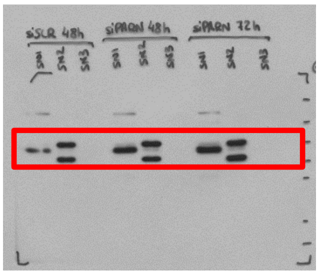

Supplement: Supplementary file 4 — Source Data [file 41467_2019_13990_MOESM4_ESM.zip › 188091_3_related_ms_4279076_q238md.pdf]
