# Supplementary material for: Identification of distinct maturation steps involved in human 40S ribosomal subunit biosynthesis
Source: Nat Commun. 2020 Jan 9;11:156. doi: 10.1038/s41467-019-13990-w (PMC6952385; doi:10.1038/s41467-019-13990-w)
Supplement: Supplementary file 2 — Peer Review [file 41467_2019_13990_MOESM2_ESM.pdf]

Reviewers' comments:

Reviewer #1 (Remarks to the Author):

In this paper "IDENTIFICATION OF DISTINCT MATURATION STEPS INVOLVED IN HUMAN 40S RIBOSOMAL SUBUNIT BIOSYNTHESIS", the authors have investigated the pathway of ribosome biogenesis in human cells (HeLa), with a close focus on the pre-40S maturation pathway.

In overall, the study addresses basic technical limitations regarding the biochemical isolation of pre-ribosomal particles from mammalian cells, such as the difficult and incomplete extraction of intact and soluble pre-ribosomal intermediates from human cell nuclei, whose nucleoli have a viscoelastic environment and are surrounded by dense heterochromatin. The authors propose the Preribosome Sequencial Extraction (PSE) method as an effective extraction method requiring low starting material.

It is a 3-step centrifugation procedure that sequentially extracts the cytoplasmic and nuclear fractions in the supernatant 1 (SN1), the more accessible nucleolar compartment in the SN2 and the less accessible nucleolar compartment in the SN3, by using buffers where the salt concentration is varied and heparin (in steps 2 and 3) and DNAse I (in step 2) are provided to dismantle and digest surrounding nucleosomes and DNA, respectively.
In addition, the authors introduce the use of the CRISPR-Cas9-mediated GFP tagging to genomically edit specific RBF (Ribosome Biogenesis Factor) genes, thus avoiding the unwanted consequences of ectopic expression (i.e. poor incorporation of protein baits into particles) and facilitating the use of tagged proteins for microscopy localization experiments and pre-ribosomal particle purification.

The application of these new methodologies allowed the authors
- to isolate and identify two pre-40S maturation precursor parrticles, here referred as pre-40S-n°1 (isolated from SN3) and pre-40S-n°2 (isolated from SN2), differently localized in the nucleolus and having different RBFs compositions
- to offer new insights about the role of specific RBFs such as PNO1 (suggested to participate in pre-40S n°1 formation), CRM1 (export role confirmed) and RRP12. In particular, RRP12 is shown to stabilize a nuclear pre-40S precursor
- RRP12 is also suggested to recruit a 3�much exonuclease activity, PARN, which apparently has no homolog in yeast and is suggested to deadenylate and trim IST1 of 18S-E pre-RNA and to be important for Rrp12 release.

General comments
It is our opinion that the applied pre-ribosome sequencial extraction method (PES) could develop into a well-used technique that allows a more efficient isolation of pre-ribosomal particles from human cells. For this reason, we think that it is an important technical advance for this research field.
After applying this sequential extraction method, the authors isolated two so-far unknown pre-40S particles. However, it appears that a further improved purification strategy (e.g tandem affinity purification) combined with sucrose gradient centrifugation, and importantly followed by SDS-PAGE/Coomassie staining of the derived pre-ribosomal particles, would be crucial for such a method.

On the other hand, the data provided on PARN as a bona-fide human pre-40S maturation factor is interesting, but the mechanistic insights in the pre-40S pathway, especially regarding the role of Pno1 and Rrp12, are yet not substantial.
Thus, although there is a signifcant methodological advance for isolation of human pre-ribosomal particles, mechanistic details of the human pre-40S pathway remain still scarce.

Specific comments
1. The combination between the Western blot analyses of fig.1b and the immunofluorescence analyses in fig.1e is of help to connect the cellular compartment (cytosol, nuclear and nucleolar compartments) to the relative supernatants (SN1, SN2, SN3). It would be further helpful to show additional

immunofluorescence analyses of RRP12 (for this immunofluorescence in presence of LMB is present in fig.5a), PES1, and TBL3 in order to increase the reader confidence respect to the compartment identity of SN1-SN2-SN3.
2. In fig.1e, caption and Methods section the number of analysed cells is not indicated.
3. Is it possible to show the Coomassie-staining of the SN3 sucrose gradients in fig.2a? Perhaps the protein content is below the detection limit? Along this line, is it possible to scale this up by using more starting material?
4. It could be informative to show a Northern of the SN2 sucrose gradient, where 18S-E pre-RNA is not stable as an additional panel to fig. 2b.
5. Why not showing in 2b the Northern blot of SN2?
6. 2d: it would be better to use a TAP purification, e.g.: flag-TEV-GFP, since the one-step purification is full of contaminants.
7. Is 2e a co-immunoprecipitation?
8. LMB block: redistribution of Noc4 and Rrp12 in SN2 and SN3 (2e lanes 11-12 and 15-16, very faint bands). LMB does not alter the particle composition in 2d, so CRM1 does not have effects in ribosome biogenesis.
9. 5a: PNO1 siRNA causes a slight accumulation of ENP1 and Rrp12 in the cytoplasm, visible in the pictures but not in the quantification.
10. fig. 7c-d: authors claim a slight accumulation of ENP1 in nucleoplasm. This is not evident from the picture in 7c and statistical analysis in 7d.

Comments
1. The knockdown experiments in general could be consolidated by showing the decrease of protein expression by western or qPCR.
2. Due to degradation problems of pre-ribosomes described in the m/s, especially in SN2, the integrity of those should be demonstrated.
3. The lack of Pno1 in all performed purifications is mentioned in the text due to quality of antibodies, in contraddiction to supplementary data explaining the loss of Pno1 via purification.
4. Figure 3b: the localization of GFP-tagged ribosomal proteins should be analyzed with more more cells.
5. Suppl Figure 3a and b: the GFP-tagged ribosomal factors Enp1 and Rrp12 (supplementary fig. 3a, c) show a clear shift into fraction SN1 compared to the untagged protein. GFP-Rrp12 additionally shows accumulation of 26S rRNA (fig. 3k). This different behavior should be explained.
6. The paragraph describing the function of Pno1 in pre-40S maturation is very unlcear.
7. The depletion of essential ribosomal protein Rrp12 can lead to pleiotropic defects, which should clearly mentioned in the text.

Reviewer #2 (Remarks to the Author):

Reviewer’s comments, manuscript Nat Communications, Nieto et al

In this manuscript Nieto et al present a method to isolate and analyze pre-ribosomal complexes from human cells using subcellular fractionation techniques coupled with GFP-TRAP affinity purification. Using this approach, the authors separate complexes from different nucleolar compartments and the nucleoplasm to analyze and identify pathway intermediates of the 40S subunit biogenesis pathway. Moreover, the authors identify PARN as a bona fide ribosome maturation factor; show that CRM1 is not involved in intranucleolar translocation of pre-ribosomes, and identify a new regulatory step involving PARN and hRRP12.

While the paper is very interesting and the method potentially useful, the data requires convincing

controls and quantification as no quantitative data, statistics or markers for each of the fractionated compartment has been presented. For publication in Nature Communications, additional experiments are necessary to show that the effects the authors describe are distinct and not artefacts of the subcellular fractionation, which are classically leaky.

Major points:

1) PSE method. While the method, based on classical subcellular fractionation, is indeed very useful and may provide additional insight into intermediates of the human ribosome biogenesis pathway, the authors should acknowledge and mention the fact that it is also a leaky method that needs to be controlled rigorously and carefully, especially when it is used to describe protein changes from one to the other fraction under certain conditions. Markers should be used for each of the compartments as loading controls in the western blots. Moreover, statistical analyses should be provided for each of the experiments.
There should be a brief passage in the main text describing what the PSE method uses, i.e. a combination of optimized cell sub-fractionation protocols etc., and a statement of how this allows to isolate things differently, or different things.
The authors explain that generally human pre-ribosomes suffer from poor solubilization and hence making it difficult to isolate them. The authors provide only one example of isolation other than PSE and that is using RIPA buffer in Fig1. RIPA buffer is a very harsh buffer that will also cause dissociation of proteins from complexes. It is therefore not an ideal comparison the authors provide. Milder buffer condition and other lysis methods have been successfully used to isolate and affinity purify human pre-ribosomes (see, for example, Scott et al., NAR 2017).

2) Page 8/Fig2b: the authors state that they cannot efficiently purify 18SE pre-rRNA or RRP12 in the SN2 fraction and claim: "This reflects the intrinsic properties of the pool of particles solubilized in that fraction rather than a technical problem, as inferred from the efficient detection of those two components in ≈40S pre-ribosomes in the subsequent extraction step (SN3) of the PSE method (Fig. 2a)."
However, upon looking at Fig 1b, lane 5, RRP12 seems to be efficiently purified in SN2, more efficiently than in SN3. That points rather to a technical problem. I would suggest experiments to rule this out (see point 1). Have the authors tried to increase the amount of RNAse and protease inhibitors in the protocol? Can the authors demonstrate that these are in fact degraded?
The authors then point towards 'intrinsic properties' as the reason (also on page 7: "We will show below that this is a consequence of the specific degradation of those preribosomal particles due to their intrinsic properties.")
What do the authors define as 'intrinsic properties'? Nowhere are these 'intrinsic properties' defined, or why an RNA that is highly structured and bound by many proteins, and thus protected, be degraded. There are some inconsistencies that need to be resolves.
On page 10, the authors then state "particles that tend to lose RRP12 and have the 18S-E pre-rRNA degraded during the extraction procedure" - which would indicate that it is due to a technical issue – this needs to be clarified.
The authors also decide to not show 18SE in figure 2b – yet the entire subsequent section of experiments is based on the data surrounding the complexes containing 18SE presented in Figs 2a, b. This data should be included.

3) Mass spectrometry data:
Page 10: Given the amount of Rio2K detected by Western blot it doesn't stand to reason why it should not be detected by MS. Did the authors investigate why Riok2 may not have been detected – i.e. number of trypsin cleavage sites, charge of expected peptides?

Generally, the authors should describe the method used for their MS analysis in the methods section in somewhat more detail, including peptide cut off, error and FDR used. There is no raw data or full data set included in the data and this should be provided, especially given the many more bands

visible in the pullout compared to control lanes. What are these proteins?

4) On page 12, the authors state that 'validation studies in SFig3 show that all GFP-tagged proteins are functional. The authors should state whether equal amounts were loaded in these western blots as ENP1 amounts look altered in the different fraction between non-tagged and GFP-tagged version (SFig3a); similarly, untagged RRP12 profiles look different in panel (c); it should also be noted that here, RRP12 is readily detectable in SN2 in both panels a and c, which does not fit with the authors' hypothesis of RRp12 degradation in SN2.
In panels e-h, localization is shown for tagged proteins and then tagged proteins upon ActD treatment – why? This is not a validation of the correct localization of the tagged protein compared to the untagged version.
5) Figure 2 a and b: Why are the arrows indicating 40S/60S different in these panels and how was that determined? Ribosomal protein loading controls should be added.

6) Page 13-14, Fig 4b,c: What do the authors mean by 'slightly loss of ENP1'? Are the large complexes or free small complexes/free proteins being referred to? This is not apparent from the figure panel and, moreover, would need to be quantified to establish that this is not a technical issue. A loading control should be added as well (ribosomal protein?)

7) GFP-Trap method (Page 29/30): The authors describe that prior to incubation with the GFP-resin, the fractions are incubated with agarose beads for 1 hour to 'eliminate non-specific binding'. Agarose is a porous material - can the authors be certain that nothing of interest to them was bound by the agarose/diffused into the agarose matrix?

Minor Points:

- Page 4: what do the authors mean by 'viscoelastic nature'? This should be explained more thoroughly.
- Page 5: The authors should mention the RBF, not present in yeast and described here by name at this point in the text.
- Fig 1X: microscopy panels are too small to make out anything.
- Overall some figure panels are too small
- The manuscript requires thorough proofreading for syntactical, grammatical and typographical errors. Some sections are difficult to understand.
- On page 26, HeLa and HCT116 cells are mentioned – where HCT cells used for any experiments in this manuscript, and if so why and where? This should be clarified.

Reviewer #3 (Remarks to the Author):

The manuscript by Nieto, et al., describes a method to biochemically fractionate human cells, using a sequential extraction method. This protocol appears to work well in generating a cellular fraction enriched for nucleolar proteins, termed SN3, for three reasons. First, SN3 is clearly enriched in a subset known nucleolar proteins. Second, the observed enrichment is lost upon treatment with Actinomycin D, which is known to cause mislocalization of nucleolar proteins. Finally, previously identified early pre-rRNA species are enriched in SN3. Based on these observations, SN3 is convincingly a nucleolar-enriched fraction that is potentially useful in the study of early ribosome biogenesis events. As the authors point out, this regulation of this process, especially in metazoans, remains largely unknown despite its importance.

Despite the extensive characterization of the fractionation protocol, the data are somewhat less convincing when using it to identify novel early pre-40S complexes. To purify these complexes, the

authors tagged the pre-40S biogenesis factor ENP1 (also called BSYL), which is associated with biogenesis particles ranging from 90S to 40S. One complex, termed pre40S-No1, was purified from the SN3 fraction, and contained RRP12 and NOP4L. A second complex, pre40S-No2, was purified from the SN2 fraction and contained "later" factors TSR1, LTV1, and NOB1. The authors posit that both complexes "must be localized in physically-different nucleolar compartments," but don't show conclusively that both particles are nucleolar. The observed localization of LTV1-GFP to the cytoplasm and nucleus, as well as the annotated localization of TSR1 and NOB1 throughout the cell, underscores this issue (Figure 3a, supplementary figure 1). While both complexes may indeed be nucleolar, it is not shown by the data in this manuscript, nor is there clear evidence for distinct nucleolar compartments.

In addition, the authors claim that these fractions represent distinct maturation stages that have not been "separated previously in human or in yeast cells." However, pre-40S particles recently have been isolated and found to have similar protein components (Ameismeier, et al, Nature 2018). In addition, the authors chose a protein, ENP1, that is bound to a range of complexes (figure 2A), but do not analyze single particles isolated from either the SN3 and SN2 fractions. The statement that these fractions are distinct from previously identified pre-ribosomal states rests on the assumption that all associated factors are present within a single complex, rather than being associated with a subset of complexes associated with ENP1, and is not supported by the data presented here. Especially in the discussion section, in which the authors equate their fractions with "downstream particles," the argument feels forced.

When the approach is applied to the case of CRM1, the authors show nicely that inhibition of CRM1 causes relocalization of some later biogenesis factors from the nucleolus to the nucleus, but not the resident nucleolar protein Noc4L. However, the conclusion, that CRM1 is required for nuclear export, and that the accumulation of 26S pre-rRNA in these cells is a secondary consequence of 90 minutes of treatment with LMB, is not surprising. Similarly, while it is comforting to know that the conserved factor PNO1 has similar roles in yeast and humans, this result is also unsurprising. Given the rationale presented in the introduction, that this method has the potential to identify differences between yeast and human biogenesis pathways, the inclusion these experiments were somewhat puzzling.

In summary, the method described in this manuscript is likely to be a useful approach in the study of early ribosome biogenesis events. However, without the use of single particle approaches, the authors overstate their case for a new maturation stage, which could be in fact a mixture of previously identified stages. In addition, the idea that their biochemical fractionation differentiates between nucleolar compartments is a fascinating one, but remains speculative at this time. The over-interpretation clouds what is otherwise a well-developed methodology that, on its own, is a valuable addition to the arsenal of protocols required to understand ribosome biogenesis.

COMMENTS TO REFEREES
MANUSCRIPT NCOMMS-18-34215

REVIEWER #1

(A) GENERAL COMMENTS

(First part). *It is our opinion that the applied pre-ribosome sequencial extraction method (PES) could develop into a well-used technique that allows a more efficient isolation of pre-ribosomal particles from human cells. For this reason, we think that it is an important technical advance for this research field.*
After applying this sequential extraction method, the authors isolated two so-far unknown pre-40S particles. However, it appears that a further improved purification strategy (e.g tandem affinity purification) combined with sucrose gradient centrifugation, and importantly followed by SDS-PAGE/Coomassie staining of the derived pre-ribosomal particles, would be crucial for such a method.

Response: We thank the reviewer for the positive comments about the PSE method. S/he also makes some recommendations to improve preribosome purifications. To avoid text redundancies, we will address these suggestions in our response to his/her **Specific comment #6** (see below, page 4) and **Additional comment #2** (see below, page 6).

(Second part). *On the other hand, the data provided on PARN as a bona-fide human pre-40S maturation factor is interesting, but the mechanistic insights in the pre-40S pathway, especially regarding the role of Pno1 and Rrp12, are yet not substantial.*
Thus, although there is a significant methodological advance for isolation of human pre-ribosomal particles, mechanistic details of the human pre-40S pathway remain still scarce.

Response: We believe that the lack of the spatiotemporal picture of the formation of human pre-40S and pre-60S intermediates, equivalent to that established in yeast during the early 2000s, is what has been holding the field back. It is our hope that the method we introduce in this manuscript will help the human studies to progress more rapidly, particularly by facilitating the dissection of the nucleolar steps and the mechanistic roles of individual human ribosome assembly factors on the level that is presently possible only in lower eukaryotes. While in this manuscript we for obvious reasons emphasized methodological aspects, we believe that our results already provide substantial new information about human ribosome assembly: (i) Direct information on different nucleolar pre-40S complexes. (ii) the RBFs associated to them. (iii) The steps in which those factors play essential roles. Specifically, some of the main findings presented in this study include:

(1) The discovery and subsequent characterization of two distinct 40S subunit maturation stages that take place in separable regions of the nucleolus. These stages occur in the interval between the 90S particles and the late pre-40S particles. To our knowledge, these stages have not been ever separated before in yeast or in humans.

(2) The identification of a new metazoan-specific step that regulates the formation of the pre-40S complexes that are ready to be exported from the nucleoplasm to the cytoplasm. This step requires the concurrent action of RRP12 and PARN, a deadenylase that works as a maturation factor for a variety of ncRNAs such as snoRNAs, telomerase RNA, and a specific subset of miRNAs. Furthermore, we demonstrate that the vast majority of PARN in the cell is involved in the maturation of intermediate pre-40S particles in the nucleolus. This discovery might have broader implications, given the association of *PARN* mutations with a number of rare human diseases [Dhanraj L et al (2015) *J. Med. Genet.* 52:539; Stuart D et al (2015) *Nat. Genet.* 47:512; Tummala H et al (2015) *J. Clin. Invest.* 125:2151].

(3) The clarification of the function of some phylogenetically-conserved RBFs (CRM1, PNO1, RRP12) whose function in human ribosome synthesis was still unsettled. In addition, our work also rules out other previously-proposed roles for those factors.

(B) SPECIFIC COMMENTS

Specific comment #1. *The combination between the Western blot analyses of fig.1b and the immunofluorescence analyses in fig.1e is of help to connect the cellular compartment (cytosol, nuclear and nucleolar compartments) to the relative supernatants (SN1, SN2, SN3). It would be further helpful to show additional immunofluorescence analyses of RRP12 (for this immunofluorescence in presence of LMB is present in fig.5a), PES1, and TBL3 in order to increase the reader confidence respect to the compartment identity of SN1-SN2-SN3.*

Response: Agree. We now include additional immunofluorescence analyses for both RRP12 and TBL3. In addition, we include a microscopy image of cells expressing PES1-GFP (see **new Supplem. Fig. 2a**). Consistent with previously-published data, these three proteins are mostly nucleolar in our cells.

The information and references regarding the subcellular localizations of these proteins (and other RBFs studied in the paper) were already summarized in **Supplementary Figure 1b**. However, given the concerns of the reviewer, we now have stated this information more clearly in the new version of our manuscript to avoid that the readers have the same problem (see **new Results section page 6**). The information provided in **Supplementary Figure 1b** is also mentioned in the legend for the **new Supplementary Figure 2**.

Specific comment #2. *In fig.1e, caption and Methods section the number of analysed cells is not indicated.*

Response: Agree. The cells analyzed in each immunofluorescence experiment were between 40 and 60. We have modified the **Methods** (page 34) section to indicate this piece of information.

Specific comment #3. *Is it possible to show the Coomassie-staining of the SN3 sucrose gradients in fig.2a? Perhaps the protein content is below the detection limit? Along this line, is it possible to scale this up by using more starting material?*

Response: This type of staining would not be informative in this case because the analyzed supernatant does not contain exclusively preribosomal particles. It contains all protein complexes present in the total-cell lysate that are extracted with the SN3 buffer. As the reviewer rightly infers, the amount of the proteins of interest is simply too low to be discernible on the background of protein complexes that co-fractionate with them in the sucrose gradients. For those reasons, in the experiments shown in **Figures 2a** and **2b**, an aliquot of each gradient fraction (1/4th) was taken to analyze pre-rRNAs by Northern blot, and the rest of the fraction was used to precipitate all proteins with TCA and detect individual RBFs by Western blot.

We comment on the analysis of purified preribosomes (not total lysates) by gradient sedimentation and Coomassie staining in our response to additional comment #2 (see below, page 6).

Specific comment #4. *It could be informative to show a Northern of the SN2 sucrose gradient, where 18S-E pre-RNA is not stable as an additional panel to fig. 2b.*

Specific comment #5. *Why not showing in 2b the Northern blot of SN2?*

Response to specific comments #4 and #5: Agree. The amount of the 18S-E species in the SN2 supernatant is relatively low. Due to this, we could not recover it efficiently from the gradient fractions in our initial experiments. However, this species can be properly detected if the experiment is performed with the maximum amount of SN2 material that can be loaded onto a single gradient (obtained from ~40 x 10^6 cells). We include this new piece of information in the **new Figure 2b**.

These new set of experiments reveals the presence of the 18S-E pre-rRNA in fractions 6-7 of the gradient. As expected, some ENP1 is also present in those two fractions. However, contrary to what happens in the case of the SN3 extracts (see **Fig. 2a**), most ENP1 is found in the upper fractions of the gradient that do not contain intact 18S-E pre-rRNA (see **Fig. 2b**, middle left panel, lanes 2 to 5). Consistent with these results, the amount of intact 18S-E that is co-precipitated with the ENP1 in the SN2 is much lower than that found in the ENP1 immunoprecipitations from the SN3 fraction (see **Fig. 2c**, compare lanes 11 and 12).

Likewise, RRP12 is also mostly detected in upper fractions of the gradient (see **Fig. 2b**, bottom left panels, lanes 3 to 5). These results indicate that the pre-40S complexes that are extracted in the SN2 fraction include a relatively large proportion of disrupted particles with the pre-rRNA degraded (there is no intact 18S-E pre-rRNA in fractions before #6). The same conclusion was reached in our original set of experiments that used more diluted lysate samples. In those experiments, the disruption of ENP1-containing complexes in the gradient analyses was less obvious than in this new set of experiments because we did not have the Northern blots for comparison. However, the presence of RRP12 in <40S complexes and the loss of 18S-E from ENP1-GFP purifications were already evident (see **first version of the manuscript, Figs. 2b** and **2c**). We also found similar results when using HeLa•ENP1-GFP cells (see **new Supplementary Fig. 5**) in experiments in which lysate preparations, gradient analyses and Northern blots were performed in parallel for SN2 and SN3. More information about this new Figure will be included in our rebuttal to the additional comment #2 of this reviewer (see below, page 6).

It is worth noting that one distinctive feature of the pre40S-No2 pool is the presence of PARN, an exoribonuclease that progressively trims the 3'-end of the 18S-E pre-rRNA. This might explain the lack of stability of these particles, since the 3'-end of the this pre-rRNA species is likely unprotected and, therefore, more prone to degradation by PARN itself and/or other nucleases during the extraction procedure. This possibility, which was already suggested in the original version of our work, is now more explicitly stated in the revised version of our manuscript (**new Results section pages 19-20**, **new Discussion section page 24**). We will discuss this issue in more detail in our response to the **additional comment #2** (see below, page 6).

The new version of the manuscript now includes the results presented in the **new Figure 2b**. We also explain our findings in more detail to make these data clearer to the readers than before. The inclusion of this figure does not alter the interpretation of the rest of experiments presented in this study or the main conclusions of our work

Specific comment #6. *2d: it would be better to use a TAP purification, e.g.: flag-TEV-GFP, since the one-step purification is full of contaminants.*

Response: The reason for using a one-step purification with GFP-TRAP, and not a TAP scheme, is the low content of No1 and No2 preribosomes in the SN3 and SN2 fractions. The high-binding efficiency of the GFP-TRAP maximizes the yield of these transient particles and, as confirmed by results of subsequent co-immunoprecipitation and microscopy analyses, allows a reliable identification of core RBFs associated to them.

We do agree, however, that some of our preparations displayed a significant number of contaminating bands. To solve this problem, we have made a modification of our initial GFP-Trap purification protocol (a two-fold dilution of the PSE fraction with the corresponding buffer). As shown in the **new Figure 2d**, this modification allows the purification of much cleaner ENP1-GFP complexes than before. Importantly, this change in the protocol does not alter the spectrum of RBFs. Furthermore, the proteins that co-purify with ENP1 are detected in similar amounts, as expected for stable components of the same particle or family of particles.

Interestingly, we have found that HEATR1 is an additional component of pre40S-No1 complexes (purified from the SN3 extract) in this new set of experiments. HEATR1 was already detected in our previous purifications, although the amount obtained was highly variable among different experiments. Supporting these new data, we have found that HEATR1 is bound to ENP-GFP in pre40S-No1 intermediates purified from ~40S gradient fractions (see **new Supplementary Fig. 5**).

In addition to the purification of ENP1-containing particles shown in **Figure 2d**, we now include improved purifications of RRP12-containing complexes in the **new Figure 6c**. These new set of data show again that RRP8 and PARN are the major co-purifying partners of RRP12.

Specific comment #7. *Is 2e a co-immunoprecipitation?*

Response: Yes, it is. This information has been already included in the original Results section (**page 10**) of our **original submission**.The text has been slightly changed to make it more clear (**new Results** page 11)

Specific comment #8. *LMB block: redistribution of Noc4 and Rrp12 in SN2 and SN3 (2e lanes 11-12 and 15-16, very faint bands). LMB does not alter the particle composition in 2d, so CRM1 does not have effects in ribosome biogenesis.*

Response: Agree. The very same conclusion is stated at the end of the first paragraph of the Results section (**page 11** of the **first version of** our **manuscript**): "*inhibition of CRM1 does not elicit any apparent intranucleolar pre-40S maturation defects"* (new Results section, page 13). We do not find any part of the description of the results that contradicts this conclusion.

Specific comment #9. *5a: PNO1 siRNA causes a slight accumulation of ENP1 and Rrp12 in the cytoplasm, visible in the pictures but not in the quantification.*

Response: Our results do show a slight accumulation of ENP1 and RRP12 in the nucleoplasm, not in the cytoplasm, of si-PNO1 cells. The reviewer points to an accumulation in the cytoplasm, but we assume that it must be a typing mistake.

We do agree that the data might appear as inconsistent and, therefore, it needs further clarification. The *PNO1* siRNA causes a slight accumulation of ENP1-GFP in both the nucleolus and nucleoplasm (see **Fig. 5a**, compare panels **f** and **n**). GFP-RRP12 is also found slightly accumulated in those two subcellular compartments (see **Fig. 5a**, compare panels **g** and **o**). The accumulation of both ENP1 and RRP12 in the two compartments is well detected in our experiments because the cell preparations are processed in parallel. Likewise, the images are also captured in parallel and processed using the very same microscopy settings. Due to this, an increase of the fluorescence/pixel in the images of si-PNO1 cells indicates an increase in the absolute content of the protein when compared to si-ctrl cells. Such a difference is not revealed

by the quantification of the epifluorescence signals from individual cells (as shown in **Fig. 5b**) because, within a cell, the relative ratios of protein in the nucleolus and nucleoplasm are not very different between the si-PNO1 and si-ctrl samples.

We can conclude therefore from those experiments that there is a slight accumulation of ENP1 and RRP12 in both the nucleolus and the nucleoplasm. However, these data also indicate that there is no differential accumulation in one of the two compartments unlike the case, for example, of the accumulation of LTV1 in both si-PNO1 and si-RRP12 cells or the accumulation of other RBFs in si-CRM1 cells (see **Fig. 5b**).

We have made changes in the new version of the manuscript (**new Results section pages 15-16**) that refers to these results to make this issue clearer to the readers. The changes in **Figure 5** and main text do not affect any of the conclusions of the work.

Specific comment #10. *fig. 7c-d: authors claim a slight accumulation of ENP1 in nucleoplasm. This is not evident from the picture in 7c and statistical analysis in 7d.*

Response: The slight accumulation of ENP1 is seen in the microscopy images shown in **Figure 7c**. It has also been quantified in **Figure 7d** (middle panel). Please, note that ENP1 forms diffuse accumulations in some regions of the nucleoplasm in **panel j**. By contrast, in **panel f**, this protein becomes uniformly distributed in cells.

In the revised version of our manuscript, we have changed **Figure 7d** in order to show the quantitations made using a larger number of cells. This new set of data demonstrates that the increase in nucleoplasm-localized ENP1-GFP is statistically significant.

(C) ADDITIONAL COMMENTS

Additional comment #1. *The knockdown experiments in general could be consolidated by showing the decrease of protein expression by western or qPCR.*

Response: Agree. We now include the qRT-PCR data for all the siRNAs employed in the study (see **new Supplementary Fig. 6a**).

Additional comment #2. *Due to degradation problems of pre-ribosomes described in the m/s, especially in SN2, the integrity of those should be demonstrated.*

Response: We addressed the issue of preribosome integrity by combining the data of the sedimentation profiles and the information about the interactions of RBFs with the 18S-E pre-rRNA. The reviewer proposes, in his/her general comment, to check integrity by Coomassie

staining of purified complexes after fractionation in sucrose gradients. Such analyses have been previously applied to purifications of major intermediates in yeast (not reported for purifications of complexes from human cells). In our case, we have found that the yield of purified complexes is not large enough to do those experiments. We have performed several trials using a new HeLa cell line, generated during this revision, that endogenously expresses ENP1-TEV-GFP. The recovery of purified complexes is too low to analyze their sedimentation behaviour. In this regard, it should be noted that the contents of the No1 and No2 intermediates in the SN2 and SN3 fractions are relatively low and the lysates cannot be concentrated without compromising the efficiency of the PSE method. As an example, in the experiment of new Supplem. Fig. 5 we had to combine the fractions from five gradients to get enough material for the Northern blots, Western blots, and GFP-Trap purifications.

Although it was not possible to analyze the sedimentation behavior of purified complexes, we provide data (summarized below) that demonstrates the integrity of the pre40S-No1 particles, the integrity of a minor pool of pre40S-No2 complexes, and the tendency to disruption of a major pool of pre-40S No2 particles. As already mentioned in the response to specific comment #4, one possible explanation for the disruption of the No2 intermediates is an intrinsic instability associated to PARN-mediated maturation of the 18S-E pre-rRNA 3'-end.

Our assessments on the integrity of preribosome preparations are the following:

(a) ***Integrity of preribosomes extracted in the SN3 fraction (pre40S-No1particles)***

(a.1) Preribosome integrity in the SN3 lysates is demonstrated by data from sucrose-gradient sedimentation analysis (**Fig. 2a**):

- All major nucleolar pre-rRNA species are readily detected in discrete and separated fractions of the gradient, indicating that the SN3 fraction contains the primary and early preribosomal complexes.
- The complexes that contain each pre-rRNA exhibit the expected molecular weights: the 47-45S pre-rRNAs are present in large (~90-100S) particles, the 30S-containing complexes undergo progressive maturation into smaller (~70S) particles, and the 21S/21SC-containing complexes render the ~40S preribosomes that contain the 18S-E pre-RNA.
- ENP1 and RRP12 are mostly concentrated in the very same ~40S fractions that contain the 18S-E pre-rRNA (Fig. 2a) and not in the first fractions of the gradient, as it would be expected if the proteins were in disrupted complexes.

(a.2) The integrity of the ENP1-GFP-containing preribosomes purified from the SN3 of HeLa•BYSL-GFP cells is demonstrated by data from the protein/RNA co-immunoprecipitation analyses (**Fig. 2c**), and the compositional analysis (**Fig. 2d**), combined with new sucrose gradient experiments (**new Supplem. Fig. 5**):

- ENP1-GFP-purified complexes mostly contain the 18S-E pre-rRNA (Fig. 2c, lane 12), consistent with a purification of the ENP1-containing preribosomes detected in the sucrose gradients (Fig. 2a and new Supplem. Fig. 5 upper left panels). ENP1 in the gradients mostly co-sediments with the 18S-E pre-rRNA.

- There are four RBFs predominantly enriched in the ENP1-GFP purifications (Fig. 2d). The amounts of the four RBFs are close to stoichiometry, as expected for common components of a homogenous set of complexes.

- RRP12 and NOC4L largely co-sediment with ENP1 and with the 18S-E pre-rRNA (Fig. 2a and new Supplem. Fig. 5 upper left panels), indicating that they are major constituents of ENP1-containing pre-40S particles.

- The interaction of ENP1 with RRP12, NOC4L and HEATR1 takes place in ~40S like complexes (new Supplem. Fig. 5), indicating that the ENP1-GFP complexes purified in our experiments are pre-40S particles that contain the 18S-E pre-rRNA and the five identified RBFs. In the experiment in Supplem. Figure 5, the interactions are detected as very weak signals, but this is probably due to a diminished yield of purified complexes caused by the lengthy manipulation and additional steps involved in the procedure. The fractions from five gradients were combined and collected in the same experiment, and the pooled fractions were subject to freezing/thawing before the binding to GFP-Trap.

(b) ***Disruption of preribosomes extracted in the SN2 fraction (pre40S-No2 particles).*** As already described in the original manuscript, a significant proportion of the pre-40S particles solubilized in the SN2 fraction are recovered as partially-disrupted complexes. These results have been further confirmed and extended with the experiment shown in **new Supplem. Figure 5**. Because the integrity of upstream and downstream preribosomes is not altered during their extraction in the SN3 and SN1 steps, the breakage of the No2 particles cannot be attributed to a general degradation problem of the PSE method. Furthermore, we have found that a large proportion of those particles contain RRP12 and PARN (**Fig. 6c**; see also in **Fig. 6d** that both proteins are mostly enriched in the SN2 fraction). This indicates that these are the intermediates that undergo PARN-mediated trimming of the 18S-E pre-rRNA and, therefore, it is likely that the 3'-end is unprotected and vulnerable to digestion during the extraction. Based on this, we pose that the lack of integrity of the No2 particles is primarily due to intrinsic properties and not to degradation caused by the PSE method.

Other pieces of circumstantial evidence also support the idea that the 3'-end of the pre-rRNA and the head region of the pre40S-No2 particles are unstable:

(b.1) Most preribosomes in the SN2 fraction do not contain 18S-E pre-rRNA molecules with intact 3'-ends (**Fig. 2b**, left panels; **Fig. 2c**, compare lanes 11 and 12; **Fig. 6a**, compare lanes 9 and 10, **new Supplem. Fig. 5**, compare intensities of 18S-E signal in the SN3 and SN2 gradients). The amounts of 18S-E pre-rRNA detected with the 3'-end probe in the

fractions with high molecular-weight complexes are minimal (**Fig. 2b**, left panels, lane 5; **new Supplem. Fig. 5**, right panels, lane 6). This is consistent with a preferential tendency to degrade the pre-rRNA through 3'-5' digestion.

(b.2) There are two abundant types of disrupted subcomplexes in the SN2 supernatant: one that contains ENP1, LTV1 and/or TSR1, and one that contains RRP12, PARN and/or RRP8 (**Fig. 2d**; **Fig. 6c**; **new Supplem. Fig. 5**, right panels and bottom panels). This indicates a tendency to separate two different sets of RBFs bound to the 18S-E 3'-end (ENP1, LTV1 and RRP12 all interact with the 3'-minor domain of the pre-rRNA). By contrast, in the upstream No1 complexes, RRP12 is stably bound and shows strong association with ENP1.

We have introduced changes in the text to improve and extend the descriptions of particle integrity. The **new Results section** now includes more-detailed descriptions of the data in Figs. 2a, 2b and 2c (**pages 8-12**) and new Supplem. Fig. 5 (**page 11**). In addition, the **new Discussion section** includes a statement about the instability of pre40S-No2 particles and its possible relationship with the presence of PARN (**page 24**).

Additional comment #3. *The lack of Pno1 in all performed purifications is mentioned in the text due to quality of antibodies, in contraddiction to supplementary data explaining the loss of Pno1 via purification.*

Response: We do agree that the text is confusing, although both explanations are correct. On one hand, the legend of former Supplem. Fig. 6 (now **Supplem. Fig. 8**) mentioned that PNO1 was not identified as a major component of the pre-40S complexes characterized in this study. This refers to the fact that PNO1 is not detected as one of the predominant bands in the silver-stained gels of purified ENP1 or RRP12 complexes (**Figs. 2d** and **6c**). On the other hand, when describing the coimmunoprecipitation/Western blot analysis of proteins associated with ENP1 in the Results section, it is pointed that the possible association with PNO1 could not be assessed because we do not have a good PNO1 antibody. Such an antibody could potentially reveal the presence of a minor pool of ENP1 complexes that contained PNO1.

Sentences have been rephrased to improve clarity [**new Results section**, **page 11**; **legend of Supplem. Fig. 8** (former Supplem. Fig. 6)].

Additional comment #4. *Figure 3b: the localization of GFP-tagged ribosomal proteins should be analyzed with more more cells.*

Response: We have increased the number of analyzed cells to 30 (results in **new Fig. 3b**). These are cell lines with endogenous GFP-tagged proteins and, therefore, the subcellular distributions are highly similar in all cells (SD values are very low). It is much more uniform than the staining with antibodies or the visualization of ectopically-expressed fluorescent proteins. The new quantifications with more cells are very similar to the previous ones and, therefore, do not affect

the conclusions of the results in Figure 3.

Similar quantifications in **Figures 5b** and **7c** have been done with more cells. Results did not change.

Additional comment #5. *5. Suppl Figure 3a and b: the GFP-tagged ribosomal factors Enp1 and Rrp12 (supplementary fig. 3a, c) show a clear shift into fraction SN1 compared to the untagged protein. GFP-Rrp12 additionally shows accumulation of 26S rRNA (fig. 3k). This different behavior should be explained.*

Response: The differential features of the ENP1-GFP and GFP-RRP12 cell lines pointed by the reviewer are now mentioned in the main text (Results section) and in legend of **Supplem. Fig. 4** (previous Supplem. Fig. 3). The reasons for the enrichment in the SN1 fraction and the difference in the pre-rRNA profile (in the case of the GFP-RRP12 cells) are uncertain, but they might be related to changes in the turnover/recycling rates of the proteins in the cytoplasm and/or a delayed import in the nucleus. These differences do not have a major impact on the 40S pathway, as indicated by the normal growth of the cell lines. Furthermore, the efficient incorporation of the GFP-fused proteins into preribosomal particles was confirmed in subsequent analysis that showed their interactions with RBFs and pre-rRNAs (Figs. 2c-2f, 6a and 6b). In the case of *HeLa•ENP1-GFP* cells, we also confirmed that the abundance and sedimentation profiles of the pre-rRNAs are normal (compare Northern blot panels of Figs. 2a and 2b with those in new Supplem. Fig. 5), indicating that the formation of preribosomes is not significantly altered. The ENP1-GFP protein, itself, shows a normal sedimentation behavior: in the SN3 it co-sediments with the 18S-E pre-rRNA and, in the SN2 is extended along the upper region of the gradient (compare ENP1 profiles in Figs. 2a and 2b with those of ENP1-GFP in new Supplem. Fig. 5).
The features of ENP1-GFP and GFP-RRP12 are now mentioned in the text of the **new Results section** (**page 10**) and **legend to Supplementary Figure 4** (previous Supplementary Fig. 3).

Additional comment #6. *The paragraph describing the function of Pno1 in pre-40S maturation is very unclear.*

Response: Agree. The paragraph has been changed to improve clarity in the new version of the manuscript (see **new Results section**, **page 14**).

Additional comment #7. *The depletion of essential ribosomal protein Rrp12 can lead to pleiotropic defects, which should clearly mentioned in the text.*

Response: Agree. We now mention this possibility in the new version of the manuscript (see **Results page 16**).

REVIEWER #2

(A) GENERAL COMMENTS

While the paper is very interesting and the method potentially useful, the data requires convincing controls and quantification as no quantitative data, statistics or markers for each of the fractionated compartment has been presented. For publication in Nature Communications, additional experiments are necessary to show that the effects the authors describe are distinct and not artefacts of the subcellular fractionation, which are classically leaky.

Response: We thank the Reviewer for the positive comments regarding our paper and the suggestions on how to improve the presentation of the results. We have now incorporated additional data that further support our conclusions.
We provide additional information regarding the reproducibility of the PSE technique in our response to the Reviewer's Major Point #1. We would also like to note that the results of PSE fractionations are fully consistent with additional data obtained by different methods (gradient sedimentation, compositional analyses of purified complexes and microscopy data). Regarding the issue of quantification, we have now also included, in the revised manuscript, the quantitation of the Western blot data derived from the PSE fractionation experiments. This issue is further addressed in our response to Major Points #1 (see below, this page) and #6 (see below, page 17).

(B) MAJOR POINTS

Major point #1. *PSE method. While the method, based on classical subcellular fractionation, is indeed very useful and may provide additional insight into intermediates of the human ribosome biogenesis pathway, the authors should acknowledge and mention the fact that it is also a leaky method that needs to be controlled rigorously and carefully, especially when it is used to describe protein changes from one to the other fraction under certain conditions. Markers should be used for each of the compartments as loading controls in the western blots. Moreover, statistical analyses should be provided for each of the experiments.*
There should be a brief passage in the main text describing what the PSE method uses, i.e. a combination of optimized cell sub-fractionation protocols etc., and a statement of how this allows to isolate things differently, or different things.
The authors explain that generally human pre-ribosomes suffer from poor solubilization and hence making it difficult to isolate them. The authors provide only one example of isolation other than PSE and that is using RIPA buffer in Fig1. RIPA buffer is a very harsh buffer that will also cause dissociation of proteins from complexes. It is therefore not an ideal comparison the authors provide. Milder buffer condition and other lysis methods have been successfully used to isolate and affinity purify human pre-ribosomes (see, for example, Scott et al., NAR 2017).

Response: It is important to point out that the PSE method is not based on classic subcellular fractionation techniques. It has been developed after our own tests and empirical observations indicating that the nucleolar preribosomes could be differentially released by modifying the concentration of both salts and Mg^{2+} after the removal of the heterochromatin layer that surrounds the nucleolus. The basis of this new method and its comparison with previous techniques have been discussed in the second paragraph of the **Discussion** both in the former (page 19) and **new (page 22)** version of our manuscript. We have also included in the new version an additional sentence in the **new Results section** (**page 6**), where we describe the main features of the procedure.

Regarding the RIPA buffer issue, this procedure was included just to show the difficulty of solubilizing human preribosomes when employing a commonly used and quite harsh cell disruption buffer. The experiment was not aimed at performing a comparative analysis of the PSE protocol with other methods previously published, like the one pointed by the Reviewer, that might possibly serve to purify nucleolar preribosomes but remain to be validated. We would also like to point out that the publication indicated by the Reviewer does not show in fact any purifications of well-defined and distinct preribosomal intermediates (that is, containing specific pre-rRNA species).

The PSE method is highly reproducible in our hands, as demonstrated by the consistency of the fractionation patterns of RBFs obtained in the control samples of all the experiments carried out throughout the paper. In addition, the use of internal control (e.g., proteins are always detected in the same SN fraction) has allowed us to demonstrate that the changes of one particular RBF from one fraction to another are specific and not due to either experimental variations among samples or to technical artifacts. Another indication of the technical soundness and consistency of our experiments is that the change in a fractionation pattern of a given protein usually occurs without the net loss of the protein. Perhaps more importantly, we would like to emphasize that we have been rather rigorous when assessing the significance of our fractionation results. For example, a change in the fractionation pattern of a given RBF has only been taken into consideration if supported by independent techniques such as co-immunoprecipitation and microscopy analyses.

Regarding the statistical analyses pointed out by the Reviewer, we have now incorporated the quantitation of all the Western blots conducted in the fractionation experiments of our manuscript (see **new Supplementary Figs. 2b** and **2d**). This quantitation has not modified the main conclusions of our work.

Major point #2. *Page 8/Fig2b: the authors state that they cannot efficiently purify 18SE pre-rRNA or RRP12 in the SN2 fraction and claim: "This reflects the intrinsic properties of the pool of particles solubilized in that fraction rather than a technical problem, as inferred from the efficient detection of those two components in ≈40S pre-ribosomes in the subsequent extraction step (SN3) of the PSE method (Fig. 2a)."*
However, upon looking at Fig 1b, lane 5, RRP12 seems to be efficiently purified in SN2, more efficiently than in SN3. That points rather to a technical problem. I would suggest experiments to rule this out (see point 1). Have the authors tried to increase the amount of RNAse and protease inhibitors in the protocol? Can the authors demonstrate that these are in fact degraded?

The authors then point towards 'intrinsic properties' as the reason (also on page 7: "We will show below that this is a consequence of the specific degradation of those preribosomal particles due to their intrinsic properties.")
What do the authors define as 'intrinsic properties'? Nowhere are these 'intrinsic properties' defined, or why an RNA that is highly structured and bound by many proteins, and thus protected, be degraded. There are some inconsistencies that need to be resolves.
On page 10, the authors then state "particles that tend to lose RRP12 and have the 18S-E pre-rRNA degraded during the extraction procedure" - which would indicate that it is due to a technical issue – this needs to be clarified.
The authors also decide to not show 18SE in figure 2b – yet the entire subsequent section of experiments is based on the data surrounding the complexes containing 18SE presented in Figs 2a, b. This data should be included.

Response: Agree. The issues regarding the **Northern blot** shown in **Figure 2b** and the degradation of the pre40S-No2 particle have been already pointed by **Referee #1**. Due to this, please check our responses to these issues given to **Referee #1's Specific Comments #4 and #5** (see above, page 3) and **Additional Comment #2** (see above, page 6).

The Reviewer also points that RRP12 is not degraded because it is well detected in the SN2 fraction. However, we never said that RRP12 becomes degraded. On the contrary, we indicated that the ENP1-containing complexes tend to lose RRP12. Furthermore, we showed that the majority of RRP12 is in subcomplexes that do not contain ENP1 or the intact pre-rRNA.

We have included in the new version of the manuscript the description of the results presented in the **new Figure 2b**. We have also devoted more time to explain in more detail these data to make the message more informative to the readers. Collectively, all these data give further support to the main conclusions of our work.

Major point #3. *Mass spectrometry data:*

Page 10: Given the amount of Rio2K detected by Western blot it doesn't stand to reason why it should not be detected by MS. Did the authors investigate why Riok2 may not have been detected – i.e. number of trypsin cleavage sites, charge of expected peptides?
Generally, the authors should describe the method used for their MS analysis in the methods section in somewhat more detail, including peptide cut off, error and FDR used. There is no raw data or full data set included in the data and this should be provided, especially given the many more bands visible in the pullout compared to control lanes. What are these proteins?

Response: Mass spectrometry was used just to identify major interactors of the GFP-fusion proteins (seen upon the silver staining of the gels). Therefore, we did not perform bulk identifications of all proteins bound to the GFP-Trap beads without electrophoretic fractionation. As expected for the presence of two preribosome intermediates (pre40S-No1 or pre40S-No2), the gels show an enriched set of bands that, upon identification, were confirmed as core components of those particles. To improve clarity, we now state in the text that mass spectrometry was used

just for the identification of the proteins detected as major bands in the silver-stained gels (see **new Methods** section [**page 34**] and legends to **Figs. 2d** and **6c**]).

Regarding the issue of RIO2, we tried to point out in the manuscript that this protein was not one of the major bands detected in the silver-stained gels derived from the analyses of ENP1-GFP-containing complexes. Due to this, it was not one of the proteins subjected to mass spectrometry characterization. However, the co-immunoprecipitation analyses did reveal that a small pool of RIO2 is forming complexes with the ENP1-GFP bait. The sentence has been rephrased to better explain the results and to avoid confusion to the readers (see **new Results** section, page 11). We apologize for this confusion.

Major point #4. *On page 12, the authors state that 'validation studies in SFig3 show that all GFP-tagged proteins are functional. The authors should state whether equal amounts were loaded in these western blots as ENP1 amounts look altered in the different fraction between non-tagged and GFP-tagged version (SFig3a); similarly, untagged RRP12 profiles look different in panel (c); it should also be noted that here, RRP12 is readily detectable in SN2 in both panels a and c, which does not fit with the authors' hypothesis of RRp12 degradation in SN2.*

In panels e-h, localization is shown for tagged proteins and then tagged proteins upon ActD treatment – why? This is not a validation of the correct localization of the tagged protein compared to the untagged version.

Response: There are similar amounts of SN1, SN2 and SN3 material in the two fractionated sets shown in new **Supplementary Figs. 4a** and **4c** (**former Supplementary Figs. 3a** and **3c**). This is inferred from the virtually-identical fractionation profiles obtained with both RRP12 (see **new Supplementary Fig. 4a**) and ENP1 (see **new Supplementary Fig. 4c**).

The enrichment found for both ENP1-GFP and GFP-RRP12 in the SN1 fraction is now described both in the **new Results section** (**page 10**) and in the legend to the **new Supplementary Fig. 4**. As indicated in our comments to **Referee #1** (see above, **Additional Comment #5**, page 10), the reason for these differences remains uncertain. However, we speculate that they could be attributed to changes in the turnover rates of the proteins within the cytoplasm and/or to delayed import rates into the nucleus. In any case, it is worth pointing out that these differences do not have a major impact on the 40S pathway since these cells show normal growth rates. In line with this, we have also shown that the GFP-fusion proteins can properly assemble onto the preribosomal particles (see **Figs. 2c-2f**, **6a** and **6b**). In the case of HeLa•ENP1-GFP cells, we have also confirmed that the abundance and sedimentation profiles of the pre-rRNAs are normal when compared to the untagged cell counterparts (for example, compare the Northern blot panels shown in **Figs. 2a** and **2b** with those displayed in the **new Supplementary Fig. 5**). Likewise, the ENP1-GFP protein itself shows a normal sedimentation behavior. Thus, in the SN3 fraction, it co-sediments with the 18S-E pre-rRNA. By contrast, in the SN2 fraction, it is found extended along the upper region of the gradient (compare the ENP1 profiles in **Figs. 2a** and **2b** with those for ENP1-GFP in the **new Supplementary Fig. 5**). Collectively, these data indicate that the formation of preribosomes is not significantly altered in any of those cells.

As stated in our response to this **Referee's Major Point #2** (see above, page 14), RRP12 is not degraded when present in the SN2 fraction. Further comments on this issue can be found in that point as well.

Regarding the ActD issue, this drug is commonly used to analyze the presence of a given protein in specific subcompartments of the nucleolus (see, for example, Shav-Tal Y et al. (2005) *Mol Biol Cell* 16: 2395, Turner AJ et al. (2012) *RNA Biol* 9: 175, and further references included in the latter work). It is known that a treatment with ActD at low concentrations in cell cultures leads to the rapid reorganization of the nucleolar architecture. These changes include the formation of caps at the nucleolar surface that contain the rDNA and proteins of both the fibrillar center (FC) and dense fibrillar component (DFC). By contrast, the proteins belonging to the granular component (GC) remain retained in the nucleolar interior under those conditions. Given that ENP1, NOC4L and RRP12 form part of early/intermediate pre-40S particles, we expected to find them in the GC in ActD-treated cells. This was, in fact, the result obtained in our experiments (see Supplementary Fig. 4e-g). We have included a new sentence about this behavior in the legend to the **new Supplementary Figure 4**.

Major point #5. *Figure 2 a and b: Why are the arrows indicating 40S/60S different in these panels and how was that determined? Ribosomal protein loading controls should be added.*

Response: After reading the comments of this Referee, we have realized that the 40S and 60S indications are quite confusing in this figure. We used the arrows to point the regions where the 40S and 60S complexes were expected to sediment in the sucrose gradients. Those are the fractions where, according to our previously published studies (not shown in the present work), the small and large subunits sediment in our polysome analyses of whole-cell lysates. The one-fraction shift of the 40S position seen in **Figure 2b** is expected because the volume of the sample loaded on that gradient was larger than 0.5 ml. In the case of gradients with whole-cell lysates, the 40S/60S positions are usually assigned according to the correspondence with the 18S and 28S peaks detected in the A254 chart or by the detection of an RPS and RPL using Western blot analyses. However, those two references cannot be used in **Figures 2a** and **2b** given that our experiments analyze cellular subfractions (SN3 and SN2) that do not contain mature ribosomes. Due to this, there is no ribosomal protein loading control that can be applied in this case. Any early-binding ribosomal protein will have a widespread distribution along the SN3 gradient given its presence in all preribosome intermediates. Having said this, we believe that there is no need to reveal the sedimentation positions of preribosomes by Western blot because they are already nicely shown by the distribution of the pre-rRNAs obtained in the Northern blot analyses of the gradient fractions.

To avoid the confusion indicated by this **Referee**, we have removed the 40S and 60S indications in **Figs. 2a** and **2b** for the reasons indicated above. They have been replaced by indications of the region that contains pre-40S (fractions with 18S-E pre-rRNA). This minor modification does not alter the results or interpretation of the experiments.

Major point #6. *Page 13-14, Fig 4b,c: What do the authors mean by 'slightly loss of ENP1'? Are the large complexes or free small complexes/free proteins being referred to? This is not*

apparent from the figure panel and, moreover, would need to be quantified to establish that this is not a technical issue. A loading control should be added as well (ribosomal protein?)

Response: We now have included the quantitation of the PSE fractionation results shown in Figure 4b to address this issue (see **new Supplementary Fig. 2d**). These data do confirm that a small amount of ENP1 is lost from the SN2 and SN1 fractions in the si-PNO1 but not in si-RRP12 cells. We also show the Western blot results for TBL3 in the samples in **new Figure 4c**. This immunoblot serves as a control to rule out that the changes observed for ENP1 could be due to variable contents of the extracts analyzed in the different experimental points. It is also important to note that the information obtained from those Western blots were validated and further extended using subsequent co-immunoprecipitation and microscopy experiments. For additional comments on the reproducibility of our experiments, see our response to **Major Comment #1** of this Referee (page 12).

Major point #7. *GFP-Trap method (Page 29/30): The authors describe that prior to incubation with the GFP-resin, the fractions are incubated with agarose beads for 1 hour to 'eliminate non-specific binding'. Agarose is a porous material - can the authors be certain that nothing of interest to them was bound by the agarose/diffused into the agarose matrix?*

Response: The preincubation was used to eliminate proteins that bind non-specifically to the matrix. With this step, we tried to avoid the pull-down of nonspecific proteins that were detected when using HeLa cells lacking any GFP-tagged protein. Our results indicate that the yield of the proteins that form specific complexes with the GFP-tagged baits used in our study is not significantly affected by this preincubation step. We just get the same bands, but free from undesired contaminants. This point was already indicated in the **Methods** section in the original version of our manuscript (now in the new version in page 33).

(C) MINOR POINTS

Minor point #1. *Page 4: what do the authors mean by 'viscoelastic nature'? This should be explained more thoroughly.*

Response: "viscoelastic nature of the internal compartments of the nucleolus" refers to the biophysical properties of the DFC as determined by Feric et al. (2016) *Cell* 165: 1686. The study describes that the DFC has viscoelastic properties and that, by comparison, the GC is more fluid. The term "viscoelastic" is also in the Merriam-Webster dictionary ("having appreciable and conjoint viscous and elastic properties"). In any case, to avoid this problem, we decided to rephrase this sentence in the new version of our manuscript (page 4). It now reads as "…a problem probably caused by both the <u>high viscosity</u> of the internal nucleolar subcompartments …".

Minor point #2. *Page 5: The authors should mention the RBF, not present in yeast and described here by name at this point in the text.*

Response: As part of the changes made to improve the writing of the manuscript, we have modified the last paragraph of the Introduction and eliminated the sentence mentioned by the Reviewer to avoid redundancies with the text in the Abstract.

Minor points #3 and #4. *Fig 1X: microscopy panels are too small to make out anything.*

- Overall some figure panels are too small

Response: Agree. We have increased the size of microscopy panels. In order to make room for these changes, we have moved the original **Figure 1e** to the **new Supplementary Figure 2a** in the new version of the manuscript.

Minor point #5. *The manuscript requires thorough proofreading for syntactical, grammatical and typographical errors. Some sections are difficult to understand.*

Response: We are sorry if that has been an issue. We have carefully rechecked the manuscript and have made corrections to improve style and clarity as best as we can. However, if the Referee stills feels that the text requires more clarity, we would be happy to submit the final version of the manuscript to a proofreading professional.

Minor point #6. *On page 26, HeLa and HCT116 cells are mentioned – where HCT cells used for any experiments in this manuscript, and if so why and where? This should be clarified.*

Response: We have used HCT116 cells in the experiments where PSE fractions were further fractionated into small and large complexes by ultracentrifugation (**Fig. 4c**). We optimized this technique in those cells because they allowed to monitor the accumulation of free RPL5/RPL11 complexes upon the induction of the nucleolar stress response with the ActD treatments. These experiments could not be done in HeLa cells because, as they are p53-deficient, they do not display an effective nuclear stress response.

The use of HCT116 in the ultracentrifugation experiments was already mentioned in the description of the PSE method in the first version of our manuscript (**Methods**, page 28). However, after seeing the Reviewer's comments, we considered that it would be informative to include a figure showing the fractionation profiles of RBFs in both HCT116 and HeLa cells using the PSE method. These data are now shown in the **new Supplementary Figure 2c** and referred to in the **new Results section (page 8)**. Our results indicate that the profiles obtained are quite similar in both cell lines, thus providing further experimental evidence in favor of both the reproducibility and efficiency of the PSE method when using different cell lines.

REVIEWER #3

Point #1. *The manuscript by Nieto, et al., describes a method to biochemically fractionate human cells, using a sequential extraction method. This protocol appears to work well in generating a cellular fraction enriched for nucleolar proteins, termed SN3, for three reasons. First, SN3 is clearly enriched in a subset known nucleolar proteins. Second, the observed enrichment is lost upon treatment with Actinomycin D, which is known to cause mislocalization of nucleolar proteins. Finally, previously identified early pre-rRNA species are enriched in SN3. Based on these observations, SN3 is convincingly a nucleolar-enriched fraction that is potentially useful in the study of early ribosome biogenesis events. As the authors point out, this regulation of this process, especially in metazoans, remains largely unknown despite its importance.*

Response: We thank the Reviewer for the positive comments about the PSE fractionation method.

Point #2. *Despite the extensive characterization of the fractionation protocol, the data are somewhat less convincing when using it to identify novel early pre-40S complexes. To purify these complexes, the authors tagged the pre-40S biogenesis factor ENP1 (also called BSYL), which is associated with biogenesis particles ranging from 90S to 40S. One complex, termed pre40S-No1, was purified from the SN3 fraction, and contained RRP12 and NOP4L. A second complex, pre40S-No2, was purified from the SN2 fraction and contained "later" factors TSR1, LTV1, and NOB1. The authors posit that both complexes "must be localized in physically-different nucleolar compartments," but don't show conclusively that both particles are nucleolar. The observed localization of LTV1-GFP to the cytoplasm and nucleus, as well as the annotated localization of TSR1 and NOB1 throughout the cell, underscores this issue (Figure 3a, supplementary figure 1). While both complexes may indeed be nucleolar, it is not shown by the data in this manuscript, nor is there clear evidence for distinct nucleolar compartments.*

Response: We respectfully disagree. The pre40S-No1 and pre40S-No2 complexes are purified from two PSE lysate fractions (SN3 and SN2) that contain nucleolar material. These fractions are obtained after the DNase- and heparin-mediated removal of the heterochromatin layer that surrounds the nucleolus. More importantly, these complexes contain RBFs that are either mostly or totally nucleolar. We apologize if this point was not made sufficiently clear in the original version of the manuscript. In the revised version, we extended the description of the results to improve clarity on this issue (**new Results section page 7**). We also make this point stronger by the quantifications of the PSE fractionation profiles obtained for the main RBFs interrogated in this study (see **new Supplementary Fig. 2**). The evidence supporting that the foregoing particles are nucleolar is the following:

(a) Both NOP14 and NOC4L are nucleolar proteins (see **Fig. 5** and Wyler et al. (2011), *RNA* 17:189). We show that NOC4L fractionates in the SN3 supernatant (**Figs. 4b** and **Supplementary Fig. 4**) and that both NOP14 and NOC4L are specific components of the

ENP1-complexes obtained from the SN3 fraction (pre40S-No1). The rest of components of pre40S-No1 particles are also nucleolar. Based on this, we conclude that the pre40S-No1 complexes must be localized in this cellular compartment.

(b) ENP1 is mostly (~85%) nucleolar (see both **Fig. 3b** and references included in **Supplementary Fig. 1b**). The proportion of ENP1 that fractionates in the SN1 supernatant (~22%) (see **Fig. 1b** and quantitation in **new Supplementary Figure 2b**) is similar to the amount of non-nucleolar ENP1. The rest of ENP1 fractionates in the SN2 (~33%) and SN3 (~44%) supernatants obtained with the PSE method. This indicates that the vast majority of ENP1 in the SN3 and SN2 fractions is nucleolar. In line with this, some of the components of ENP1-containing complexes in the SN3 (pre40S-No1 particles) and in the SN2 (pre40S-No2 particles) fractions such as NOC4L and RRP12 are also localized in the nucleolus.

(c) Similarly to ENP1, RRP12 shows a preferential (~82%) nucleolar localization (see **Fig. 3b** and references in **Supplementary Fig. 1b**). Consistent with this, only a small fraction (10%) of RRP12 fractionates in the SN1 supernatant (see **Fig. 1b** and quantitation included in the **new Supplementary Fig. 2b**). The rest of RRP12 is found in the SN2 (~56%) and SN3 (34%) fractions (**Fig. 1b** and **new Supplementary Fig. 2b**). This indicates again that the vast majority of the RRP12-containing complexes solubilized in the SN2 (subcomplexes of pre40S-No2 particles) are nucleolar. Further supporting this, we found that: **(i)** RRP12 associates with PARN, another nucleolar protein (see references in **Supplementary Fig. 1b**). **(ii)** PARN, as expected, is also detected in the SN2 fraction (**Fig. 6d**).

(d) As controls, we show two non-nucleolar proteins (tubulin and PCNA) that are preferentially enriched in the SN1 fraction of the PSE method (see **Fig. 1b**).

Point #2. *In addition, the authors claim that these fractions represent distinct maturation stages that have not been "separated previously in human or in yeast cells." However, pre-40S particles recently have been isolated and found to have similar protein components (Ameismeier, et al, Nature 2018). In addition, the authors chose a protein, ENP1, that is bound to a range of complexes (figure 2A), but do not analyze single particles isolated from either the SN3 and SN2 fractions. The statement that these fractions are distinct from previously identified pre-ribosomal states rests on the assumption that all associated factors are present within a single complex, rather than being associated with a subset of complexes associated with ENP1, and is not supported by the data presented here. Especially in the discussion section, in which the authors equate their fractions with "downstream particles," the argument feels forced.*

Response: We respectfully disagree. Against the Reviewer's statement, the pre-40S complexes characterized by Ameismeier et al. do not include any particles containing NOP14, NOC4L, HEATR1 or PARN. Therefore, the preribosomal complexes identified here are distinct from those reported in that paper. To our knowledge, our intermediates had not been ever separated or described in any other previous study.

We do agree that ENP1 can bind to different complexes. However, we were fortunate in finding that the PSE method can separate two pools of nucleolar ENP1-containing complexes that are composed of different RBF subsets. This indicates that there must be different preribosomal

intermediates. In our view, there is no need to perform single-particle analyses to reach this conclusion. In fact, the experimental strategy followed by us is quite similar to those utilized in the seminal studies that established the association/dissociation patterns of RBFs onto the main ribosome precursors in yeast (for a review on this topic, see References 1-5 in our new version of the manuscript). This approach combines the use of the protein content of purified particles, gradient sedimentation analyses of RBFs, and the microscopy inspection of the subcellular localization of specific RBFs. The dissection of the pre-rRNA species present in the interrogated particles, the pattern of overlapping components between them, and the subcellular localization of the associated RBFs also provides quite useful information regarding the directionality of the pathway followed by the particles under investigation. Using this strategy, we can conclude that the SN3, SN2 and SN1 fractions contain early nucleolar particles, nucleolar intermediate particles and nucleoplasmic/cytoplasmic late particles, respectively. Therefore, in our view, it is correct to refer to the particles solubilized in the SN1 and SN2 fractions as "downstream particles" relative to those found in the SN3 fraction. As inferred from their components, it is clear that most of the intermediate stages characterized by Ameismeier et al. have to be located downstream of our pre40S-No2 particles. Taken together, we believe therefore that our study has opened the possibility to unveil, in the near future, the structural details associated with the maturation of human pre-40S particles in the nucleolus through single-particle analyses of the pre40S-No1 and pre40S-No2 complexes identified here. However, tackling this issue is clearly beyond the scope of the current manuscript.

Point #3. *When the approach is applied to the case of CRM1, the authors show nicely that inhibition of CRM1 causes relocalization of some later biogenesis factors from the nucleolus to the nucleus, but not the resident nucleolar protein Noc4L. However, the conclusion, that CRM1 is required for nuclear export, and that the accumulation of 26S pre-rRNA in these cells is a secondary consequence of 90 minutes of treatment with LMB, is not surprising. Similarly, while it is comforting to know that the conserved factor PNO1 has similar roles in yeast and humans, this result is also unsurprising. Given the rationale presented in the introduction, that this method has the potential to identify differences between yeast and human biogenesis pathways, the inclusion these experiments were somewhat puzzling.*

Response: Our tools allowed us to analyze intermediate-nucleolar (pre40S-No2) preribosomes separately from both upstream early-nucleolar (pre40S-No1) and downstream nucleoplasmic/cytoplasmic ones. This gave us the opportunity to define which factors are involved in the formation and transport of the pre-40S particles from the nucleolus to the cytoplasm. Addressing this issue was not possible before our work. It is worth noting that those biosynthetic steps still remain ill-defined in yeast. Furthermore, the correlation of RBF functions between humans and yeast has remained unclear because there is no straightforward parallelism between the steps of the two pathways. For example, it is known that human cells have more pre-rRNA processing steps than yeast (e.g., the exonucleolytic processing of the 21S-C and 18S-E pre-rRNAs). They also exhibit different maturation kinetics (e.g., the steps between the emergence of pre-40S particles in the nucleolus and the export to the cytoplasm take place much faster in the case of yeast). In line with this, previous data obtained upon either the chemical

inhibition or protein depletion of CRM1, PNO1 and RRP12 indicated that these proteins might have non-conserved roles in human ribosome synthesis. For example, CRM1 was proposed to play a role in the maturation of the pre-40S particles that contain the 26S pre-rRNA (Rouquette J. et al. (2005) *EMBO J* 24: 2862). By contrast, we show here that this protein is primarily related with the nuclear export of ribosomal particles. Likewise, PNO1 was proposed to participate in the maturation of the 26S-containing particles. Our work, however, has demonstrated that this protein is in fact essential for the formation of pre40S-No1 particles (a step downstream of 26S processing). Finally, we have also identified a RRP12-dependent step that participates in the maturation of the 40S subunit in human but not yeast cells. These results, in our view, are relevant to clarify the steps of the human pathway as well as to identify points of phylogenetic conservation and divergence of this route across species.

Point #4. *In summary, the method described in this manuscript is likely to be a useful approach in the study of early ribosome biogenesis events. However, without the use of single particle approaches, the authors overstate their case for a new maturation stage, which could be in fact a mixture of previously identified stages. In addition, the idea that their biochemical fractionation differentiates between nucleolar compartments is a fascinating one, but remains speculative at this time. The over-interpretation clouds what is otherwise a well-developed methodology that, on its own, is a valuable addition to the arsenal of protocols required to understand ribosome biogenesis.*

Response: We thank again the Reviewer for his/her kind comments on the interest and usefulness of the PSE method described in our manuscript. However, we disagree that single particle analyses are needed to confirm the pre-40S maturation stages unveiled by our work. The reasons for this have already been stated in our response to the Referee's **Point #2** (see above, page 23).

Regarding the issue of the nucleolar compartmentalization, the PSE fractionation data are consistent with the idea that the granular component of the nucleolus contains different subcompartments that are characterized by specific solubilization properties. As an example, please note that RRP12 and PES1 become solubilized in different supernatants of our PSE method (SN2 and SN3, respectively) despite the fact that they are both localized in the granular component of the nucleolus (see **new Supplementary Fig. 2a**). This supports the idea that specific preribosomal particles are physically segregated in distinct nucleolar subregions. We do agree with the Reviewer that the existence of such subcompartments remains to be formally demonstrated. This was, in fact, the reason for keeping this idea mainly confined to the **Discussion** rather than to other sections of our manuscript.

Given that the Reviewer finds that this issue was somewhat overstated in our manuscript, we have decided to modify several parts of the text to make more explicit that this is still in a hypothetical stage. Thus, the sentence indicating the existence of "physically-different nucleolar subcompartments" has now been changed to "our data reveal that there are two distinctive and biochemically-separable pools of ~40S precursors" (see **new Results section, page 11**). In the new Discussion section, we also state now that "An additional idea that emerges from our results is that the No1 and No2 intermediates might be physically segregated in different

subcompartments of the nucleolus given their distinctive solubilization in the SN2 and SN3 extracts of the PSE method” (see **new Discussion section, page 24**).

MANUSCRIPT NCOMMS-18-34215
SUMMARY OF CHANGES MADE FROM ORIGINAL SUBMISSION

(1) Text

1.1 Relevant change in Results section (page 9). Paragraph has been changed to describe the results of the sedimentation analyses in new Fig. 2b. It addresses specific comment #4 and comment #2 of Reviewers 1 and 2, respectively.

1.2 Relevant change in Results section (pages 8-12) and Discussion (page 24). Text has been extended to comment on the integrity of the pre-40S particles. It also includes the description of data from co-immunoprecipitation analysis of ENP1-GFP complexes purified from pools of sucrose gradient fractions (new Supplementary Fig. 5). It addresses, together with experiments shown in Figs. 2a, 2b and 2c, additional comment #2 of Reviewer 1.

1.3 Changes in Results and Discussion sections (pages 11 and 24). Text has been rephrased to make it clear that the possible presence of physically distinct subcompartments in the GC of the nucleolus is a speculative idea. Addresses comment #4 of Reviewer 3.

1.4 Minor change in Results section (page 6). Additional sentence to mention the main features of the PSE method. Related to major point #1 of Reviewer 2.

1.5 Changes in Results section (pages 6-7). Sentence extended to mention that Supplementary Fig. 1b compiles the known information about the RBFs analyzed in the initial PSE fractionation experiments and additional statement about the subcellular localization of RRP12. Related to specific comment #1 and point #2 of Reviewers 1 and 3, respectively.

1.6 Addition in Results section (page 8). Sentence to mention the information in new Supplementary Fig. 2c. Complements the answer to minor point #6 of Reviewer 2.

1.7 Change in Results section (page 10) and legend of Supplementary Figure 4. New sentences to mention differences in the PSE fractionation profile and pre-rRNA processing pattern in the ENP1-GFP and GFP-RRP12 edited cell lines, as recommended by Reviewer #1 (additional comment #5).

1.8 Minor change in Results section (page 11) and legend to Supplementary Figure 8. Sentences about the lack of detection of PNO1 has been rephrased to improve clarity. It addresses additional comment #3 of Reviewer 1.

1.9 Minor changes in Results section (page 11) and in legend of Supplementary Fig. 8 (former Supplementary Fig. 6). Sentences about the lack of detection of RIO2 have been rephrased to improve clarity. It addresses major comment #3 of Reviewer 2.

1.10 Minor change in Results section (page 14). Improved description of PNO1 function, as suggested by Reviewer #1 (additional comment #6).

1.11 Minor change in Results section (page 15-16). Changes in ENP1 and RRP12 localization in PNO1-knockdown cells are described in a more-precise manner (Fig. 5a). It addresses specific comment #9 of Reviewer 1.

1.12 Minor change in Results section (page 16). The possibility of pleiotropic defects upon RRP12 depletion is mentioned, as suggested in comment #7 of Reviewer 1.

1.13 Minor change in the Methods section (page 34). The number of cells analyzed in immunofluorescence analyses is indicated. It addresses specific comment #2 of Reviewer 1.

1.14 Addition in the Results section (pages 19-20) and Discussion section (page 24). Statement about the instability of pre40S-No2 particles and its possible relationship with the presence of PARN. Related to additional comment #2 of Reviewer 1 and major point #2 of Reviewer 2.

1.15 Addition in Methods section (pages 32-33). The section describing sucrose gradient analyses includes now the procedure followed for the experiment in Supplementary Fig. 5. Related to additional comment #2 of Reviewer 1.

1.16 Minor change in Methods section (page 33) and legends to Figs. 2d and 6c. It is indicated that major bands in gel were sliced and identified by mass spectrometry. Related to major point #3 of Reviewer 2.

1.17 Minor change in title page. The information about author's affiliations was modified to make it more precise and non-redundant.

1.18 Minor change in nomenclature. The name RIO2 has been adopted, both in the text and figures, to refer to the protein kinase RIO2. This nomenclature is the one recommended in two major databases (NCBI, PIR) and have been used in most publications about this protein. In the previous version of the manuscript the name of the gene (RIOK2) was used to refer to the protein.

1.19 Minor change in one sentence of Introduction (page 4). The term "viscoelastic" (considered unclear by Reviewer 2, minor point #1) was changed, and used "high viscosity" instead.

1.20 General corrections in the text of all sections. Made to improve style and clarity. Related to minor point #5 of Reviewer 2.

(2) Figures

2.1 Change in original Fig. 1 and new panels in Supplementary Fig. 2. Previous Fig. 1e has been moved to Supplementary Fig. 2a. We added, in new Supplementary Fig. 2a, new panels that show the subcellular localization of TBL3, RRP12 and PES1. It addresses specific comment #1 of Reviewer 1.

2.2 New Figure 2b. It shows sedimentation profiles of preribosome components (including the 18S-E pre-rRNA) in the SN2 lysates. It addresses specific comments #4 and major point #2 of Reviewers 1 and 2, respectively.

2.3 New Figure 2d. It shows stained gels of improved ENP1-GFP complex purifications. It addresses specific comment #6 of Reviewer 1.

2.4 New Figure 6c. It shows stained gel of improved GFP-RRP12 complex purifications. It addresses specific comment #6 of Reviewer 1.

2.5 Modifications in Figs. 3b, 5b and 7d. Graphs correspond to quantifications made with more cells than the previous ones. This addresses additional comment #4 of Reviewer 1.

2.6 New Supplementary Figs. 2b and 2d. Quantifications of Western blots of the RBF fractionation profiles obtained with the PSE method under different cellular conditions. It addresses major point #1 and #6 of Reviewer 2.

2.7 New Supplementary Figure 2c. It shows the fractionation patterns of several RBFs in HeLa and HCT116 cells. This figure was included to show the reproducibility and efficiency of the PSE method in two different cell lines. The figure was not directly requested by any of the Reviewers, but it is helpful information that complements the answer to minor point #6 of Reviewer 2.

2.8 New Supplementary Figure 5. Shows co-immunoprecipitation analysis of ENP1-GFP complexes purified from pools of sucrose gradient fractions. It addresses additional comment #2 of Reviewer 1.

2.9 New Supplementary Figure 6a. qPCR data showing the effects of siRNAs on gene expression. Suggested in additional comment #1 of Reviewer 1.

2.10 Addition of new panels in Figure 4c. It shows that the amounts of material were similar in all samples. It addresses major point #6 of Reviewer 2.

2.11 Minor changes in Figs. 2a and 2b. Fractions enriched in 18S-E pre-rRNA are indicated as pre-40S. Addresses major point #5 of Reviewer 2.

2.12 Minor change in figures: Substitution of the name RIOK2 by RIO2 in the Western blot panels of the kinase RIO2.

(3) Figure legends

3.1 Supplementary Fig. 4 (former Supplementary Fig. 3). Legend mentions differential features of the BYSL-GFP and GFP-RRP12 cell lines and the distribution of proteins upon ActD treatment (addresses comment #5 of Reviewer 1 and major point #4 of Reviewer 2). The legend also includes a new sentence mentioning the change in subcellular localization of the proteins in ActD-treated cells (related to major point #4 of Reviewer 2).

3.2 Supplementary Fig. 6 (former Supplementary Fig. 4). Legend includes the description of new Fig. 6a.

3.3 Legends to new Figures. Legends corresponding to the new Supplementary Fig. 2 and new Supplementary Fig. 5.

Reviewers' comments:

Reviewer #1 (Remarks to the Author):

We are happy with the revision and support publicstion of this manuscript in Nature Communications

Reviewer #2 (Remarks to the Author):

Reviewer’s comments, revised manuscript Nat Communications, Nieto et al.

In this revised version of the manuscript by Nieto et al., the authors have addresses some of the reviewers’ comments, however, there are several that were left unanswered or were simply not addressed.

One issue is still the overinterpretation of the data; firstly, in the sense that it is not fully conclusive that the particles considered by the authors as nucleolar are actually such. Secondly, in terms of the authors stating to use the method to especially address differences between yeast and human pre-ribosomes that previous methods were not able to address, while most of the results seem to be the same as in yeast (and have been partially shown by others already). And, thirdly, and most importantly, the authors keep stating that their approach extracts intact pre-ribosomes (as opposed to other techniques). This is a point that is highly contentious and unproven in this manuscript – please see my detailed comments on that issue below.

Considering the fact that the method as such is not novel per se (it has been used in other organisms before to separate pre-ribosomal complexes), that some results are not conclusive in terms of extraction of ‘nucleolar’ particles, and that it appears to potentially cause degradation of complexes during extraction, I believe the data is still not convincing enough for publication in Nature Communications.

1)Pre-ribosome degradation during extraction.

The question of degradation was raised in the first review already. On page 13 of the rebuttal, the authors state that while this reviewer “<i>also points that RRP12 is not degraded because it is well detected in the SN2 fraction. However, we never said that RRP12 becomes degraded. On the contrary, we indicated that the ENP1-containing complexes tend to lose RRP12.</i>”

I would like to draw the authors’ attention to page 8 of the original manuscript, where they wrote the following: “<i>These data indicate that the 18S- E pre-rRNA tends to be degraded and RRP12 is also degraded or released from the ≈40S pre-ribosomes obtained in the SN2 step.</i>”

Throughout the revised manuscript, the authors repeatedly suggest- on the one hand - that the pre-40S No2 complex is a ‘in vivo’ occurring particle, yet the data is not supportive of that.
Is the pre-rRNA in this particle polyadenylated, i.e. targeted by TRAMP for exosome degradation?

On page 9, it is noted that these ‘degraded particles’ make up the majority of isolated complexes – which is worrying. Why should they make up a majority? Especially, since on page 8 of the authors’ rebuttal it is said that “this indicates that these are the intermediates that undergo PARN-mediated trimming of the 18S-E pre-rRNA and, therefore, it is likely that the 3’-end is unprotected and vulnerable to digestion during the extraction.” – suggesting, on the other hand, that these particle are not ‘in vivo’ once but extraction artefacts.

And lastly, that authors themselves state on page 11-12, the authors state that:

“<i>Collectively, our data reveal that there are two distinctive and biochemically-separable pools of ~40S precursors: (i) An earlier set of intermediates (referred to hereafter as pre40S-No1), which includes the initial complexes formed upon generation of the 18S-E pre-rRNA, contains ENP1, HEATR1, RRP12, NOP14, NOC4L, and intact 18S-E pre-rRNA species. However, it lacks cytoplasmic-maturation RBFs. (ii) A later set of intermediates (referred to as pre40S-No2), which includes complexes harboring ENP1, cytoplasmic-maturation RBFs, and unstable 18S-E pre-rRNAs. These intermediate particles tend to generate ENP1- and RRP12- containing subparticles during the extraction procedure.</i>”

Intrinsic properties. This was not addressed by the authors in the revised manuscript or rebuttal. As previously mentioned, the authors point towards ‘intrinsic properties’ as the reason for the degradation of complex No2 – but what are these? The term is also mentioned in the rebuttal but not explained.

“<i>We will show below that this is a consequence of the specific degradation of those pre-ribosomal particles due to their intrinsic properties.</i>”

What are the ‘different solubilization and stability properties’ (rev. MS page 9)?

Why is an RNA that is highly structured and bound by many proteins, and thus protected, degraded at this point in vivo?

In the original manuscript the author stated on page 8: “<i>This reflects the intrinsic properties of the pool of particles solubilized in that fraction rather than a technical problem, as inferred from the efficient detection of those two components in ≈40S pre-ribosomes in the subsequent extraction step (SN3) of the PSE method (Fig. 2a).</i>”

In the revised version, the authors refer to “intrinsic structural features” (page 9) instead – which ones? Where does Rrp12 sit on the pre-rRNA that the supposed loss of it would cause 18S-E pre-rRNA to be degraded? The reasoning here is rather vague.

The authors have also not addressed the fact that the loss of complexes in samples lysed with RIPA buffer is due to dissociation of proteins from complexes, as RIPA is a harsh buffer, and not the inferiority of the method per se or insolubility of nucleolar complexes.

2)Mass spectrometry data. The fact that selected bands were excised from the gel and analyzed by MS should be mentioned in the text as well as the figure legend, not only the methods section as it is confusing to the reader and misleading.
Page 11 – with regards to the Enp1-GFP particles, the authors state that “<i>they also confirm that the complexes extracted in the SN3 supernatant are intact pre-ribosomes whereas those present in the SN2 supernatant include a large proportion of partially-disrupted particles.</i>”

How so – only selected components were analyzed. A full proteomic analysis of both Enp1-GFP associated SN2 and SN3is necessary.

It should also be noted that a full proteomic analysis of the particles may have helped a more thorough conclusion of whether the extracted particles are indeed all nucleolar.

Minor Points:

- Page 4: '<i>in our species</i>' – which ones 'humans' (plural?); this should be changed back to 'human cells' – particularly since the study is carried out in tissue culture cells.
- The manuscript still requires some proofreading for syntactical and grammatical (i.e., first sentence of result section).

MANUSCRIPT NCOMMS-18-34215A

SECOND REVISION STAGE

COMMENTS TO REFEREE #2

(A) GENERAL COMMENTS

In this revised version of the manuscript by Nieto et al., the authors have addresses some of the reviewers' comments, however, there are several that were left unanswered or were simply not addressed.

One issue is still the overinterpretation of the data; firstly, in the sense that it is not fully conclusive that the particles considered by the authors as nucleolar are actually such. Secondly, in terms of the authors stating to use the method to especially address differences between yeast and human pre-ribosomes that previous methods were not able to address, while most of the results seem to be the same as in yeast (and have been partially shown by others already). And, thirdly, and most importantly, the authors keep stating that their approach extracts intact pre-ribosomes (as opposed to other techniques). This is a point that is highly contentious and unproven in this manuscript – please see my detailed comments on that issue below.

Considering the fact that the method as such is not novel per se (it has been used in other organisms before to separate pre-ribosomal complexes), that some results are not conclusive in terms of extraction of 'nucleolar' particles, and that it appears to potentially cause degradation of complexes during extraction, I believe the data is still not convincing enough for publication in Nature Communications.

Response:

In the first round of reviews, this Referee found the paper interesting but raised two main points of concern: **(1)** The need to include appropriate controls and quantifications to validate the PSE method. **(2)** The need to clarify the nature of the preribosome degradation in the SN2 fraction. In addition, s/he also raised additional (11) minor points that could be easily addressed by simply modifying some parts of the main text.

As stated in much detail in our previous rebuttal letter, the second version of our manuscript (NCOMMS-18-34215A) addressed all those points with the incorporation of new experiments (for the two major points) and with modifications in the text or figures (for the rest of points). We are happy that the reviewer did not find any problem in the way we have addressed the majority of his/her concerns. However, the reviewer still considers that the second major point has not been fully answered and, based on that, she/he concludes that the PSE method does not extract intact preribosomes. As we will discuss below, we respectfully disagree with the Referee's view on this issue.

In addition, s/he now raises new issues that had not been considered in the first round of review. These concerns were related to the information and data already contained in the first (not in the second) round of review. Specifically, she/he now points out that: **(1)** It is unclear that the purified preribosomes are nucleolar. **(2)** The method does not seem useful to uncover differences between yeast and human. **(3)** The method is not novel as we have claimed. We also disagree with all these statements for the reasons indicated below.

1. *It is not fully conclusive that the particles are nucleolar.*

Response: We respectfully disagree. As already indicated in our response to Referee #3 in our former rebuttal letter, the experimental data provided in the paper fully supports the nucleolar localization of the particles described. In this context, it is worth mentioning that this aspect of the study has not been questioned by Referee #1 upon reviewing the resubmitted version of our manuscript. We summarize again the information that, in our opinion, does support the nucleolar localization of the identified particles:

Firstly, as indicated in our previous rebuttal letter, the complexes found by us in the SN2 and SN3 fractions of our method contain RBFs that are either totally or mostly nucleolar. Evidence supporting this idea includes:

(a) The fact that both NOP14 and NOC4L are exclusively nucleolar proteins (see **Fig. 5** and Wyler et al. (2011), *RNA* 17:189). Furthermore, we show that NOC4L fractionates in the SN3 supernatant (**Figs. 4b** and submitted **Supplementary Fig. 4**) and that both NOP14 and NOC4L are specific components of the ENP1-complexes obtained in the SN3 fraction (pre40S-No1). The rest of components of pre40S-No1 particles are also nucleolar. Based on this, we conclude that the pre40S-No1 complexes are localized in this cellular compartment.

(b) The fact that ENP1 is mostly (~85%) nucleolar (see both **Fig. 3b** and references included in **Supplementary Fig. 1b**). In fact, the small proportion of ENP1 that fractionates in the SN1 supernatant (~22%) (see **Fig. 1b** and quantitation in the submitted **Supplementary Figure 2b**) is similar to the amount of non-nucleolar ENP1. **This indicates that the vast majority of ENP1 in the SN2 and SN3 fractions is nucleolar**. In line with this, it is worth noting that some of the components of ENP1-containing complexes in the SN3 (pre40S-No1 particles) and in the SN2 (pre40S-No2 particles) fractions such as NOC4L and RRP12 are also localized in the nucleolus.

(c) As in the case of ENP1, we have also shown that RRP12 shows a preferential nucleolar localization (~82%; see **Fig. 3b** and references in **Supplementary Fig. 1b**). Consistent with this, only a small fraction (10%) of RRP12 fractionates in the SN1 supernatant (see **Fig. 1b** and quantitation included in the **new Supplementary Fig. 2b** already included in the NCOMMS-18-34215A manuscript). By contrast, the rest of RRP12 is found in the SN2 (~56%) and SN3 (34%) fractions (**Fig. 1b** and **new Supplementary Fig. 2b**). **This indicates that the vast majority of the RRP12-containing complexes solubilized in the SN2 (subcomplexes of pre40S-No2 particles) are nucleolar**. Further supporting this idea, we found that: **(i)** RRP12 associates with PARN, another nucleolar protein (see references in **Supplementary Fig. 1b**). **(ii)** PARN, as expected, is also detected in the SN2 fraction (**Fig. 6d**).

(d) As controls, we have shown that two non-nucleolar proteins (tubulin and PCNA) are preferentially detected in the SN1 fraction of the PSE method in the second version (NCOMMS-18-34215A) of our work (see **Fig. 1b**).

2. *The method was not proven useful to address differences between yeast and humans, and that some information had already been shown by others.*

Response: We respectfully disagree. We believe that the manuscript does provide novel findings of significant value for the field. Those include:

(a) The discovery and subsequent characterization of two distinct intermediate 40S subunit maturation stages. These stages occur in the interval between the 90S particles and the late pre-40S particles. To our knowledge, **these stages have not been ever separated before in yeast or in humans**.

(b) The **identification of a new metazoan-specific step that regulates the formation of the pre-40S complexes that are ready to be exported from the nucleoplasm to the cytoplasm**. This step requires the concurrent action of RRP12 and PARN, a deadenylase that works as a maturation factor for a variety of ncRNAs such as snoRNAs, telomerase RNA, and a specific subset of miRNAs. Furthermore, we demonstrate that the vast majority of PARN in the cell is involved in the maturation of intermediate pre-40S particles in the nucleolus. This discovery might have broader implications, given the association of *PARN* mutations with a number of rare human diseases [Dhanraj L et al (2015) *J. Med. Genet.* 52:539; Stuart D et al (2015) *Nat. Genet.* 47:512; Tummala H et al (2015) *J. Clin. Invest.* 125:2151]. Additional, hitherto unknown data contained in this section include: **(i)** The functional relationship between PARN and RRP12. **(ii)** The implication of RRP12 and PARN in the final maturation of pre-40S particles. **(iii)** The fact that this new regulatory step is not present in yeast.

(c) The **clarification of the function of some phylogenetically-conserved RBFs** (CRM1, PNO1, RRP12) **whose function in human ribosome synthesis was still unsettled**. In addition, **our work also rules out other previously-proposed roles for those factors**. The functions of CRM1 and PNO1 appear to be conserved in yeast and humans, but this issue was not clear. **RRP12 was shown to have a role in nuclear export, not in maturation, of pre-40S particles in yeast**. Here we show that, in humans, RRP12 is essential for the stability and maturation (together with PARN) of nucleoplasmic pre-40S particles.

3. *It is not proven that the method extracts intact pre-ribosomes as opposed to other methods*.

Response: We respectfully disagree. We have shown extensive data demonstrating that the PSE method does allow the purification of a nucleolar intermediate (pre40S-No1 particle) that had not previously been identified using any other extraction method. The integrity of the pre40S-No1 particles in our preparations has also been demonstrated (see below). This part of the study specifically proves that the PSE method can indeed extract intact preribosomes. Data in favor of this conclusion includes:

(a) The demonstration of the integrity of preribosomes extracted in the SN3 fraction (pre40S-No1 particles). This point has been already discussed in our previous rebuttal letter (see our comments to **Additional Point #2** and **Major Point #2** of Reviewers 1 and 2, respectively). In any case, here is again the main experimental evidence supporting our conclusions:

(a.1) Sucrose-gradient sedimentation analysis demonstrating the integrity of preribosomes

obtained in SN3 lysates (**Fig. 2a**). This evidence includes:

- The demonstration that all the major nucleolar pre-rRNA species can be readily detected in discrete and separated fractions of the gradients (thus indicating that the SN3 fraction does contain the primary and early preribosomal complexes).
- The finding that the complexes that contain each pre-rRNA exhibit the expected molecular weights. Thus, the 47-45S pre-rRNAs are present in large (~90-100S) particles, the 30S-containing complexes undergo progressive maturation into smaller (~70S) particles, and the 21S/21SC-containing complexes are matured to form the ~40S preribosomes that contain the 18S-E pre-RNA.
- The data showing that the ENP1 and RRP12 are mostly concentrated in the very same ~40S fractions of the gradients that contain the 18S-E pre-rRNA (**Fig. 2a**). By contrast, they are not present in the first fractions of the gradient as it would be expected if the proteins were present in disrupted complexes.

(a.2) Protein/RNA co-immunoprecipitation experiments (**Fig. 2c**), compositional analyses (**Fig. 2d**), and sucrose gradient experiments (provided in the **new Supplem. Fig. 5** included in NCOMMS-18-34215A) demonstrating the integrity of the ENP1-GFP-containing preribosomes purified from the SN3 of HeLa•BYSL-GFP cells. This evidence indicates that:

- The ENP1-GFP-purified complexes mostly contain the 18S-E pre-rRNA species (**Fig. 2c**, lane 12), a result consistent with the purification of the ENP1-containing preribosomes detected in the sucrose gradients (**Fig. 2a**; **new Supplem. Fig. 5** of NCOMMS-18-34215A, upper left panels). Furthermore, we show that ENP1-GFP mostly co-sediments with the 18S-E pre-rRNA species in those gradient experiments.
- The detection of four RBFs predominantly enriched in the ENP1-GFP purifications according to our MS analyses (**Fig. 2d**). The amounts of these RBFs are close to stoichiometry, as expected for common components of a homogenous set of complexes.
- The observation that RRP12 and NOC4L largely co-sediment with both ENP1 and with the 18S-E pre-rRNA, indicating that they are major constituents of the ENP1-containing pre-40S particles (**Fig. 2a** and the **new Supplem. Fig. 5**, upper left panels, included in NCOMMS-18-34215A).
- The observation that the interaction of ENP1 with RRP12, NOC4L and HEATR1 takes place in ~40S like complexes (see the **new Supplem. Fig. 5** included in NCOMMS-18-34215A). This finding indicates that the ENP1-GFP complexes that have been purified in our experiments are indeed pre-40S particles that contain the 18S-E pre-rRNA species and the five identified RBFs.

(b) In addition to the pre40S-No1 intermediates, we have demonstrated that the PSE method can effectively fractionate an additional pool of preribosomes (pre40S-No2 particles) that includes both intact particles and subparticles. Several lines of evidence indicate that some of the assembly intermediates present in this pool are relatively unstable during extraction (see our answer to

Specific Point #1 further below). Importantly, it should be mentioned that this part of the study does not question the capacity of the PSE method to extract intact preribosomes (that has been well demonstrated by the analyses carried out with the pre40S-No1 particles).

4. *On the issue that the method is not novel per se and that it has been used in other organisms before to separate pre-ribosomal complexes*.

Response: We respectfully disagree. We would appreciate it if the Referee could be more specific about the purported use of our method (or any kind of sequential extraction of different ribosome assembly intermediates) in previous studies.

On our side, we are not aware of any other published study that has used our method to fractionate and purify preribosomes in humans or any other organism. In fact, we have devoted a part of the Discussion of the manuscript to provide a historic perspective on the approaches, so far unfruitful, that have been attempted in the past to overcome problems associated with the inefficient purification of human preribosomes (page 21).

(B) SPECIFIC POINTS

Specific Point #1. *Pre-ribosome degradation during extraction. The question of degradation was raised in the first review already. On page 13 of the rebuttal, the authors state that while this reviewer "also points that RRP12 is not degraded because it is well detected in the SN2 fraction. However, we never said that RRP12 becomes degraded. On the contrary, we indicated that the ENP1-containing complexes tend to lose RRP12."*

I would like to draw the authors' attention to page 8 of the original manuscript, where they wrote the following: "These data indicate that the 18S- E pre-rRNA ***tends to be degraded and RRP12 is also degraded*** *or released from the ≈40S pre-ribosomes obtained in the SN2 step."*

Response: Agree. We apologize for this oversight. This was certainly a mistake. In fact, that statement was already modified in the NCOMMS-18-34215A version of our manuscript.

Specific Point #1 (continued). *Throughout the revised manuscript, the authors repeatedly suggest- on the one hand - that the pre-40S No2 complex is a 'in vivo' occurring particle, yet the data is not supportive of that. Is the pre-rRNA in this particle polyadenylated, i.e. targeted by TRAMP for exosome degradation?*

On page 9, it is noted that these 'degraded particles' make up the majority of isolated complexes – which is worrying. Why should they make up a majority? Especially, since on page 8 of the authors' rebuttal it is said that "this indicates that these are the intermediates that undergo PARN-mediated trimming of the 18S-E pre-rRNA and, therefore, it is likely that the 3'-end is unprotected and ***vulnerable to digestion during the extraction.****" – suggesting, on the other hand, that these particle are not 'in vivo' once but extraction artefacts.*

And lastly, that authors themselves state on page 11-12, the authors state that: "Collectively, our data reveal that there are two distinctive and biochemically-separable pools of ~40S precursors: (i) An earlier set of intermediates (referred to hereafter as pre40S-No1), which

includes the initial complexes formed upon generation of the 18S-E pre-rRNA, contains ENP1, HEATR1, RRP12, NOP14, NOC4L, and intact 18S-E pre-rRNA species. However, it lacks cytoplasmic-maturation RBFs. (ii) A later set of intermediates (referred to as pre40S-No2), which includes complexes harboring ENP1, cytoplasmic-maturation RBFs, and unstable 18S-E pre-rRNAs. ***These intermediate particles tend to generate ENP1- and RRP12- containing subparticles during the extraction procedure****."*

Response: **We respectfully disagree.** As already described in the original manuscript, a significant proportion of the pre-40S particles solubilized in the SN2 fraction is recovered as partially-disassembled complexes. These results have been further confirmed and extended with the experiments included in the second version (NCOMMS-18-34215A) of the manuscript (**new Supplem. Figure 5**).

The presence of smaller-sized preribosome subparticles in a preparation can be due to three possible causes: **(1)** Disruption of some full-size preribosomes during experimental manipulation. **(2)** Existence of pre-assembly subcomplexes inside the cells that serve as building blocks for their incorporation onto preribosomes. **(3)** Existence of post-assembly subcomplexes in the cells generated either during normal release from preribosomes or during disassembly of defective preribosomes induced by the quality-control machinery. Regardless of the specific cause involved, all three of them imply that the detected semi-stable preribosome fraction reflects an aspect of preribosome assembly in the cell and not an extraction artefact.

Currently, we favor the cause #1 to explain the detection of subparticles in the pre40S-No2 preparation. Given that pre40S-No1 particles remain intact during their extraction with the PSE method, we reason that there must be some "intrinsic" (we could alternatively call it "specific") properties of the pre40S-No2 particles that make them particularly unstable during the extraction steps. We speculate that the pre40S-No2 particles are those undergoing the PARN-mediated maturation step (in which the pre-rRNA 3'-end must be unprotected and thereby subjected to potential nuclease attack), but we feel the mechanistic basis for their vulnerability is a separate interesting issue that is beyond the scope of this study where we primarily aim to introduce and validate the PSE method.

As already indicated in our first rebuttal letter, we believe that these pre40S-No2 particles represent actual steps in the ribogenesis because:

(a) **Our method preserves the integrity of the preribosomes located upstream and downstream of the pre40S-No2 particles during their extraction in the SN3 and SN1 steps.** Due to this, the breakage of the No2 particles cannot be attributed to a general degradation problem of the PSE method.

(b) We have found that a large proportion of these semi-stable particles contain RRP12 and PARN (**Fig. 6c**; see also that both proteins are mostly enriched in the SN2 fraction in **Fig. 6d**). **These data indicate that these particles are the biosynthetic intermediates that undergo PARN-mediated trimming of the 18S-E pre-rRNA** (and that, therefore, where the 3'-end of this pre-rRNA is most likely unprotected and vulnerable during the extraction step). Other pieces of circumstantial evidence also support the idea that the 3'-end of the pre-rRNA and the head region of the pre40S-No2 particles are unstable:

- Most preribosomes in the SN2 fraction do not contain 18S-E pre-rRNA molecules with

intact 3'-ends (**Fig. 2b**, left panels; **Fig. 2c**, compare lanes 11 and 12; **Fig. 6a**, compare lanes 9 and 10, **Supplem. Fig. 5**, compare intensities of 18S-E signal in the SN3 and SN2 gradients). Furthermore, the amounts of 18S-E pre-rRNA detected with the 3'-end probe in the fractions with high molecular-weight complexes are minimal (**Fig. 2b**, left panels, lane 5; **Supplem. Fig. 5**, right panels, lane 6). This is consistent with a preferential tendency to degrade the pre-rRNA through 3'-5' digestion.

- There are two abundant types of disrupted subcomplexes in the SN2 supernatant. One of them contains ENP1, LTV1 and/or TSR1. The other one harbors RRP12, PARN and/or RRP8 (**Fig. 2d**; **Fig. 6c**; **Supplem. Fig. 5**, right panels and bottom panels). This indicates a tendency to separate two different sets of RBFs bound to the 18S-E 3'-end (ENP1, LTV1 and RRP12 all interact with the 3'-minor domain of the pre-rRNA). By contrast, in the upstream No1 complexes, RRP12 is stably bound and shows strong association with ENP1.

Specific Point #1 (continued). *Is the pre-rRNA in this particle polyadenylated, i.e. targeted by TRAMP for exosome degradation?*

Response: As shown in **Fig. 2c** and **Supplem. Fig. 5**, the amount of 18S-E pre-rRNA (detected with the 5'-ITS probe) co-precipitating and co-sedimenting with ENP1-containing complexes in the SN2 extract is very low. This indicates that most of those complexes do not have polyadenylated 18S-E pre-rRNA.

In addition, our MS data have not detected any TRAMP component associated with ENP1-containing complexes, as it would be expected if such complexes were undergoing active, TRAMP-mediated degradation.

Specific Point #1 (continued). *Intrinsic properties. This was not addressed by the authors in the revised manuscript or rebuttal. As previously mentioned, the authors point towards 'intrinsic properties' as the reason for the degradation of complex No2 – but what are these? The term is also mentioned in the rebuttal but not explained.*

"We will show below that this is a consequence of the specific degradation of those pre-ribosomal particles due to their intrinsic properties."

What are the 'different solubilization and stability properties' (rev. MS page 9)?

Why is an RNA that is highly structured and bound by many proteins, and thus protected, degraded at this point in vivo?

In the original manuscript the author stated on page 8: "This reflects the intrinsic properties of the pool of particles solubilized in that fraction rather than a technical problem, as inferred from the efficient detection of those two components in ≈40S pre-ribosomes in the subsequent extraction step (SN3) of the PSE method (Fig. 2a)."

In the revised version, the authors refer to "intrinsic structural features" (page 9) instead – which ones? Where does Rrp12 sit on the pre-rRNA that the supposed loss of it would cause 18S-E pre-rRNA to be degraded? The reasoning here is rather vague.

Response: "Intrinsic properties". As stated in the response to the previous comments above, our findings are consistent with the pre40S-No2 particles having less stability during extraction than the pre40S-No1 particles. As indicated above, we posit that this property may be due to the pre-rRNA's 3'-end being unprotected.

With "Different solubility properties", we wish to indicate the fact that the pre40S-No1 and No2 particles are extracted with different buffers (different fractions of the PSE method).

In any case, and given that this Reviewer finds these terms unclear and/or confusing, we have decided to change some parts of the text to improve clarity and avoid similar confusion by the readers. The sentences that alluded to "intrinsic properties" or pre-rRNA degradation as possible reasons for pre40S-No2 instability (pages 9, 10 and 19) have been removed from the Results section. These ideas are now only discussed in the Discussion section (page 23). This change does not affect any of the conclusions of the work.

Specific Point #1 (continued). *The authors have also not addressed the fact that the loss of complexes in samples lysed with RIPA buffer is due to dissociation of proteins from complexes, as RIPA is a harsh buffer, and not the inferiority of the method per se or insolubility of nucleolar complexes.*

Response: We respectfully disagree. We believe that the Reviewer is using a quite incoherent argument here: if RIPA is a harsh buffer that causes dissociation of proteins from complexes, that implies that it is clearly inferior (not to say useless) to carry out the type of studies that have been done in our work.

In any case, we believe that the Referee is also wrong in his/her inference. The dissociation of complexes by the RIPA buffer **SHOULD NOT PREVENT** the detection of the released proteins in the total cellular lysates obtained. However, such proteins are not detected in our immunoblot analysis (**Figure 1b**, compare for example the amounts of TBL3 or FBL in lane 1 with those in lane 6). This indicates that the complexes cannot in fact be solubilized with the RIPA buffer, or that some of the proteins extracted under these harsh conditions aggregate once they are stripped from the RNP complexes (our unpublished data), effectively preventing their detection in the soluble fraction.

As stated above in this rebuttal letter, we reiterate again that there are no other methods, to our knowledge, that allow the type of analyses carried out in this work in human cells. Serious problems and caveats associated with previous methods have been underscored in our Discussion section (page 21).

Specific Point #2. *Mass spectrometry data. The fact that selected bands were excised from the gel and analyzed by MS should be mentioned in the text as well as the figure legend, not only the methods section as it is confusing to the reader and misleading.*

Page 11 – with regards to the Enp1-GFP particles, the authors state that "they also confirm that the complexes extracted in the SN3 supernatant are intact pre-ribosomes whereas those present in the SN2 supernatant include a large proportion of partially-disrupted particles."

How so – only selected components were analyzed. A full proteomic analysis of both Enp1-GFP associated SN2 and SN3 is necessary.

It should also be noted that a full proteomic analysis of the particles may have helped a more thorough conclusion of whether the extracted particles are indeed all nucleolar.

Response: We respectfully disagree. We believe that **the data clearly show that each of the sets of ENP1-containing complexes purified in our study are enriched in specific maturation intermediates**. Furthermore, **our analyses have identified the main core components of these particles**. For example, in the case of the ENP1-containing complexes purified form the SN3 fraction, four nucleolar RBFs have been found predominantly enriched in them: HEATR1, RRP12, NOP14 and NOC4L (**Fig. 2d**, left panel). Notably, the relative amounts of those four proteins are close to stoichiometry, the expected behavior for common components of a homogenous set of complexes.

It is also worth mentioning that we have done a significant amount of work to confirm these proteomics results using independent experiments (sucrose gradient sedimentation analyses, co-IPs). A similar approach was followed to identify the components of the ENP1-containing complexes purified from the SN2 fraction.

We believe that the full proteomic analyses suggested by the Reviewer will not add much to the main take-home message of our work. Certainly, it will only contribute to re-discover again the core particle components described above and, perhaps, to identify other peripheral proteins that are present in minor amounts in the purified complexes. Because they are not expected to provide an incremental amount of knowledge, these new experiments would cause an unnecessary delay in the publication of this work given the time required to execute and replicate them.

It is also important to note that the identification of major co-purifying proteins revealed by PAGE gel staining has been used before in many seminal studies to identify the composition of major preribosome precursors in yeast [see examples in Grandi et al. (2002) *Mol Cell* 10:105 and Schaffer et al. (2003) *EMBO J* 22:1370].

Minor Point #1. *Page 4: 'in our species' – which ones 'humans' (plural?); this should be changed back to 'human cells' – particularly since the study is carried out in tissue culture cells.*

Response: Agree

Thank you, we have modified the text to "human cells" instead of "our species".

Minor Point #2. *The manuscript still requires some proofreading for syntactical and grammatical (i.e., first sentence of result section).*

Response: Agree. We have modified the sentence pointed out by the Reviewer. We have done a thorough proofreading of the entire manuscript and corrected a few mistakes and typos. Changes made in the new version of the manuscript can be seen in the attached text-comparison file.
